# The Use of Bridging Ligand Substituents to Bias the Population of Localized and Delocalized Mixed‐Valence Conformers in Solution

**DOI:** 10.1002/chem.202200926

**Published:** 2022-06-23

**Authors:** Parvin Safari, Simon Gückel, Josef B. G. Gluyas, Stephen A. Moggach, Martin Kaupp, Paul J. Low

**Affiliations:** ^1^ School of Molecular Sciences University of Western Australia 35 Stirling Highway Crawley, WA 6009 Australia; ^2^ Institut für Chemie Theoretische Chemie/Quantenchemie Sekr. C7 Technische Universität Berlin Straße des 17. Juni 135 10623 Berlin Germany

**Keywords:** conformation analysis, density functional calculations, mixed-valent compounds, ruthenium

## Abstract

The electronic structure and associated spectroscopic properties of ligand‐bridged, bimetallic ‘mixed‐valence’ complexes of the general form {M}(μ‐B){M^+^} are dictated by the electronic couplings, and hence orbital overlaps, between the metal centers mediated by the bridge. In the case of complexes such as [{Cp*(dppe)Ru}(μ‐C≡CC_6_H_4_C≡C){Ru(dppe)Cp*}]^+^, the low barrier to rotation of the half‐sandwich metal fragments and the arylene bridge around the acetylene moieties results in population of many energy minima across the conformational energy landscape. Since orbital overlap is also sensitive to the particular mutual orientations of the metal fragment(s) and arylene bridge through a Karplus‐like relationship, the different members of the population range exemplify electronic structures ranging from strongly localized (weakly coupled Robin‐Day Class II) to completely delocalized (Robin‐Day Class III). Here, we use electronic structure calculations with the hybrid density functional BLYP35‐D3 and a continuum solvent model in combination with UV‐vis‐NIR and IR spectroelectrochemical studies to show that the conformational population in complexes [{Cp*(dppe)Ru}(μ‐C≡CArC≡C){Ru(dppe)Cp*]^+^, and hence the dominant electronic structure, can be biased through the steric and electronic properties of the diethynylarylene (Ar) moiety (Ar=1,4‐C_6_H_4_, 1,4‐C_6_F_4_, 1,4‐C_6_H_2_‐2,5‐Me_2_, 1,4‐C_6_H_2_‐2,5‐(CF_3_)_2_, 1,4‐C_6_H_2_‐2,5‐^i^Pr_2_).

## Graphical abstract

**Mixed‐valence complexes** [{Cp*(dppe)Ru}(μ‐C≡CArC≡C){Ru(dppe)Cp*]^+^ are known to exist as a mixture of conformations, with electronic character varying between the localized and delocalized limits in response to the particular mutual orientations of the metal and bridge fragments. Electronic structure calculations using the hybrid density functional BLYP35‐D3 and a continuum solvent model in combination with spectroelectrochemical studies have demonstrated that the conformational population, and hence the dominant electronic structure, can be biased through the steric and electronic properties of the diethynylarylene (Ar) moiety.

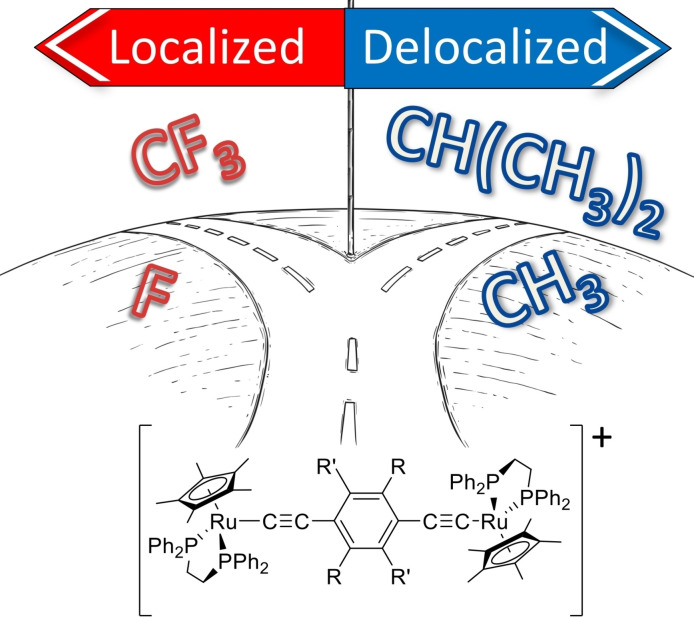

## Introduction

**Figure 1:**
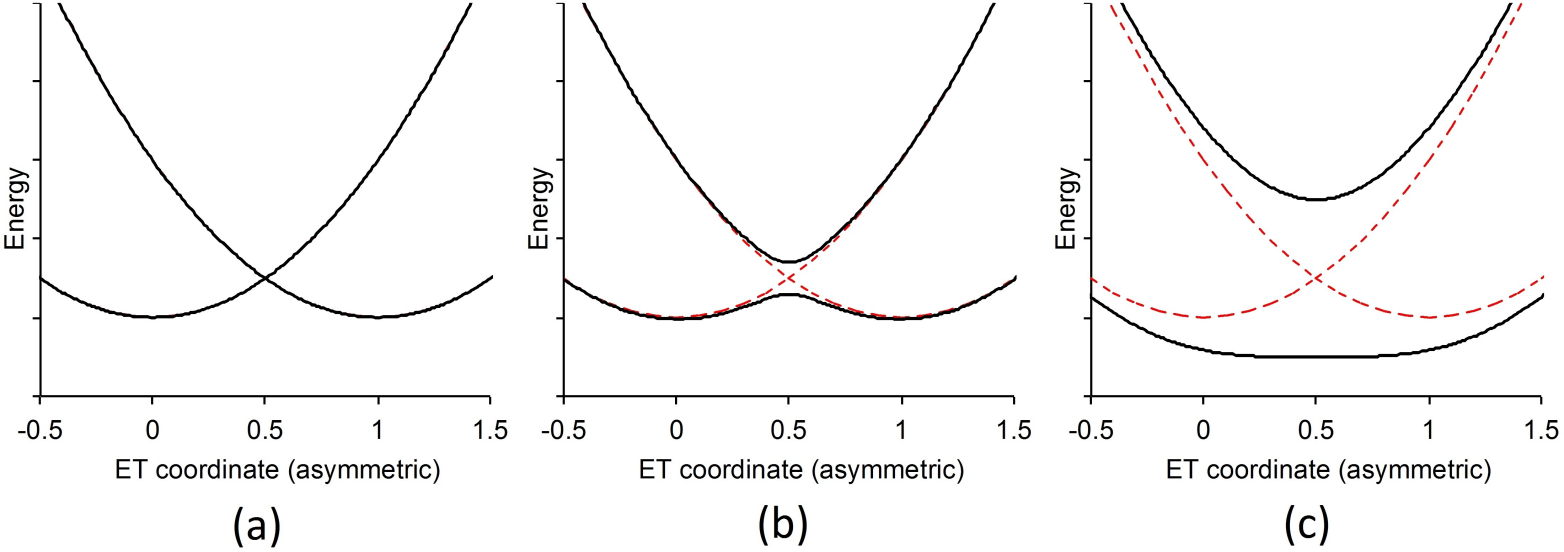
Illustrative plots of the adiabatic free energy surfaces of a degenerate mixed‐valence complex illustrating the cases of Robin‐Day a) Class I, b) Class II, and c) Class III. The diabatic free energy curve is shown as a red dashed trace for reference.

Mixed‐valence complexes {M}(μ‐B){M^+^} in which two redox sites, {M} and {M^+^}, identical in every respect bar oxidation state, are linked through some bridging moiety, B, have long served as archetypal model systems through which to study intramolecular electron transfer processes.^[1]^ This is due in no small part to the ability to design and prepare examples of {M}(μ‐B){M^+^} systems in which the electronic structure varies between the extremes of being strongly localized to fully delocalized. Given these variations in electronic structure, the Robin‐Day classification scheme which describes mixed‐valence complexes in terms of the degree of electronic coupling between the redox sites {M} and {M^+^}, has been widely adopted (Figure 1).^[2]^ In this scheme, Class I contains compounds in which the redox sites are valence trapped with no electronic coupling between them. Class II contains compounds that are valence trapped but with a degree of electronic coupling between the redox sites (*H*_AB_) that permits ground‐state electron exchange over a thermal energy barrier, Δ*G*_th_*. Optical electron transfer can occur following vibrational relaxation of the Franck‐Condon state formed by optical excitation at an energy λ, giving rise to an intervalence charge transfer (IVCT) transition, which often is observed as a weak band in the NIR region of the electronic spectrum. For Class II systems with small electronic coupling terms such that 0 < *H*_AB_ < λ/2, the thermal (*E*_th_) and optical (*E*_op_) barriers to electron transfer are simply related as Equation 1

(1) $$\lambda =4\;\Delta G{}_{\mathrm{th}}{}^{*}$$

As electronic coupling increases the thermal barrier to electron transfer decreases, and at the point where Δ*G*_th_*=0, the system moves into Class III, which contains valence de‐trapped complexes with delocalized ground states, and (Eq. 2)

(2) $$\lambda =2H{}_{\mathrm{AB}}$$

Whilst the IVCT bands of more weakly coupled systems are typically Gaussian in shape, as the electronic coupling increases, the IVCT band becomes more intense and progressively cut off on the low energy side.^[1d]^ As the complex becomes better described as a fully delocalized system the IVCT band‐shape evolves to a more symmetric absorption band and is perhaps more accurately termed a charge resonance or simply π‐π* transition.^[3]^ Molecular vibrations along the charge transfer coordinate are also important in refining the IVCT band‐shape.^[4]^

Although the analysis of the electronic and optical properties of mixed‐valence (MV) complexes in terms of such a ‘two‐state’ model (Figure 1) has been a powerful tool, and the assignment of MV complexes to one of the Robin‐Day classes has provided a convenient language to advance discussions around these concepts, an increasing number of studies have identified examples of MV compounds and complexes that adopt multiple low‐lying conformations.^[5]^ Since one factor in determining the electronic coupling between the redox sites is the orbital overlap integral along the {M}(μ‐B){M^+^} chain, the population of different structural minima within a sample can lead to the simultaneous observation of the spectroscopic features ranging across the characteristics from strongly localized to delocalized systems.[^5b^, ^6^] Obviously, complexes that exist as such populations with varying electronic structure cannot be accurately assigned to a single Robin‐Day class. The overlap and significantly different molar extinction of the various distinct IVCT and/or charge resonance bands arising from the weakly and more strongly coupled (or delocalized) members of the population, together with the presence of additional electronic transitions of similar energy arising from local dd and/or MLCT/LMCT transitions make analysis of individual members of these populations within the Marcus‐Hush ‘two‐state’ model fraught with difficulty.^[7]^

**Figure 2:**
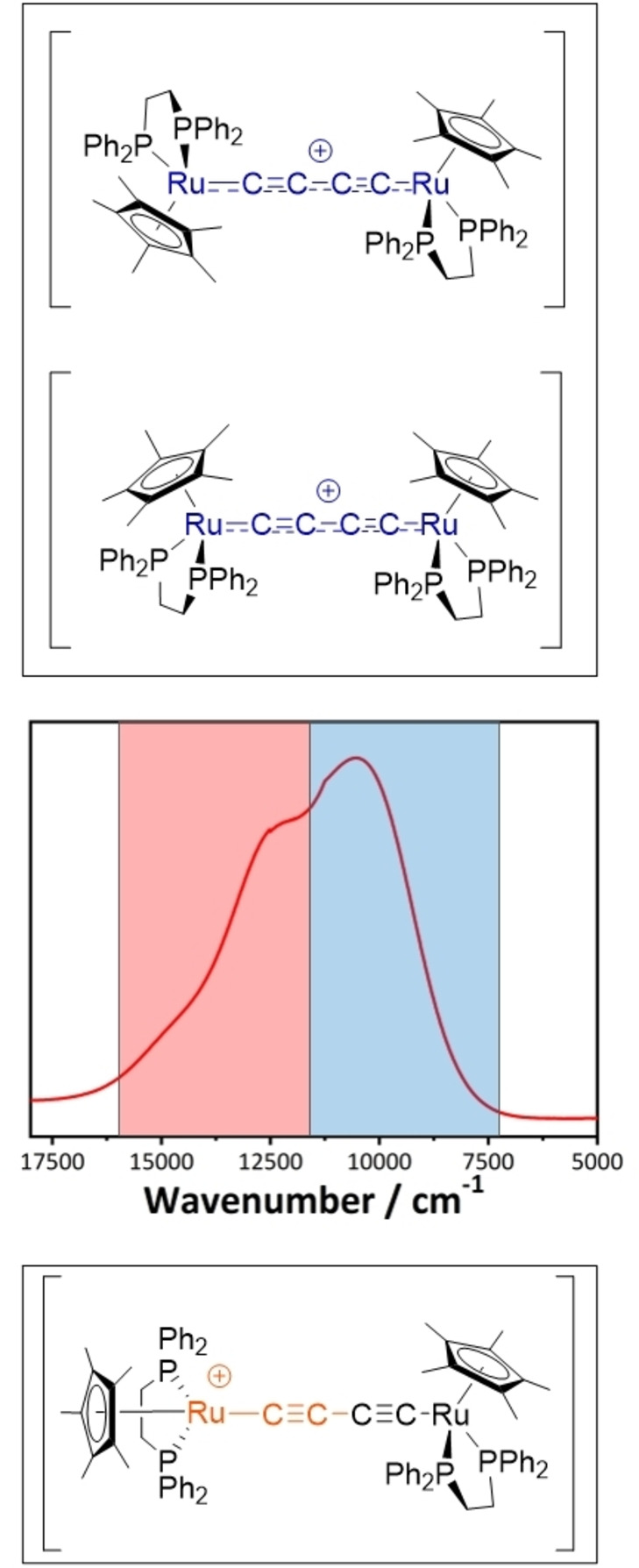
Plots of the NIR spectra of [{Ru(dppe)Cp*}_2_(μ‐C≡CC≡C)]^+^ illustrating the influence of the distribution of molecular conformations on the spectral envelope. The majority of transitions in the red shaded region arise from *perpendicular* conformers whilst the more *cis* and *trans*‐like conformers give rise to a greater proportion of the transitions that fall predominantly in the blue shaded region.

As an illustration of these ideas, the electronic properties of linearly bridged ruthenium‐based radical cations of the form {[Ru(pp)Cp’]_2_(μ‐C≡C−C≡C)}^+^ (pp=(PPh_3_)_2_, dppe, μ‐{PPh_2_(CH_2_)_5_PPh_2_}_2_, Cp’=Cp, Cp*) can be purposely tuned by using the ancillary ligands to control the accessible range of molecular conformations (Figure 2).^[6d]^ In comparison with the {Ru(PPh_3_)_2_Cp} supported complex, in which the metal fragments rotate relatively freely around the long molecular Ru−C≡C−C≡C−Ru axis, the use of 1,2‐bis(diphenylphosphino)ethane and pentamethylcyclopentadiene ancillary ligands in {Ru(dppe)Cp*} results in an increase in non‐covalent interactions between the metal fragments that stabilize the ‘perpendicular’ conformers with more localized electronic structures. This biasing of the conformational distribution is reflected in the appearance of the NIR spectra (Figure 2). Restrictions to the degrees of conformational freedom by linking the two metal centers through bridging PPh_2_(CH_2_)_5_PPh_2_ ligands has a similar effect, but in this case stabilizes the more delocalized *cis*‐like conformer.^[6f]^

**Figure 3:**
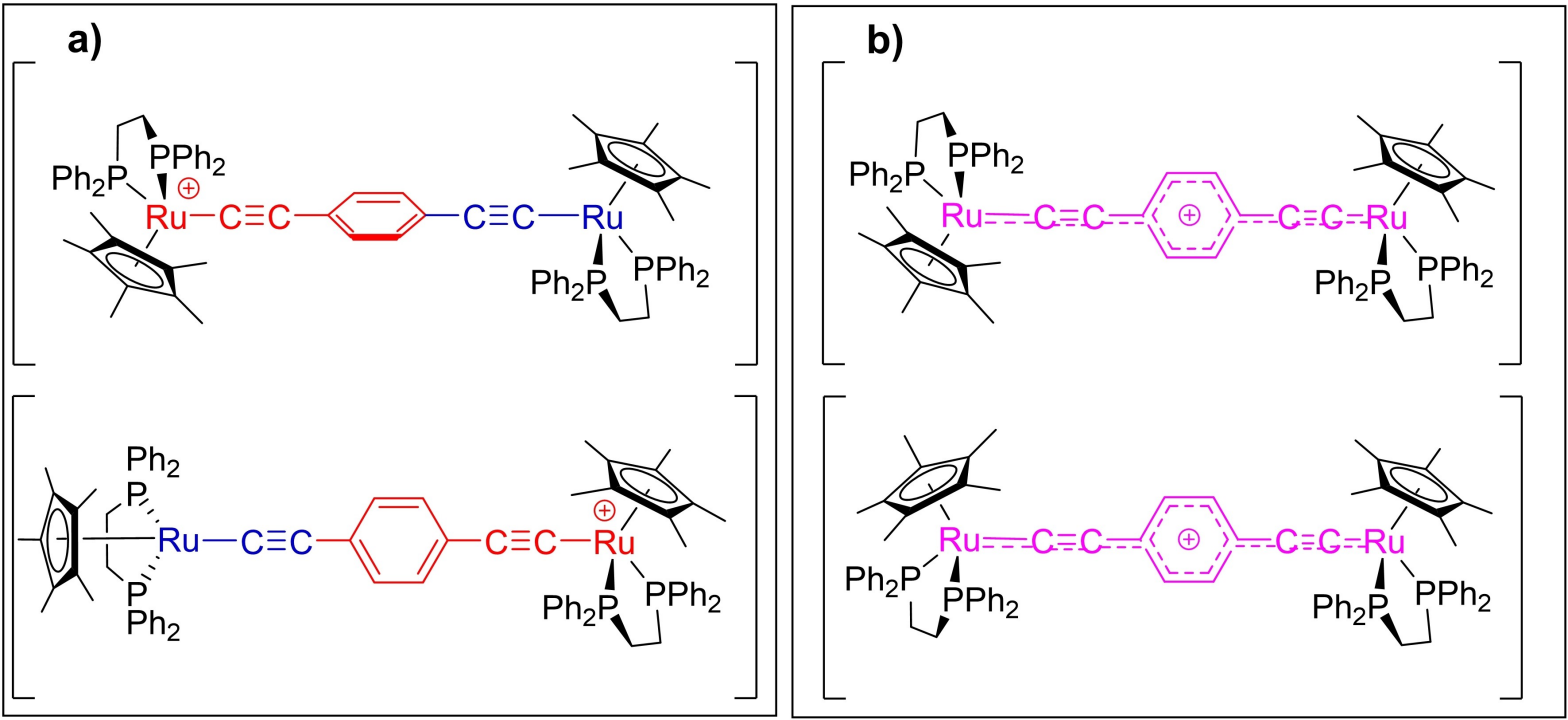
Sketches illustrating the classical descriptions of [**1**]^+^ as: a) localized (Class II); and b) delocalized (Class III) valence isomers.

In the case of mixed‐valence complexes featuring bridging ligands without cylindrical symmetry about the long molecular axis, such as 1,4‐diethynyl benzene in [{Ru(dppe)Cp*}_2_(μ‐C≡CC_6_H_4_C≡C)]^+^ ([**1**]^+^), the conformational space, and hence the underlying electronic structures and mixed‐valence characteristics, is further complicated by the relative conformation of the metal centers not only with respect to each other, but also with respect to the plane of the central phenylene ring (Figure 3).^[6e]^

**Scheme 1:**
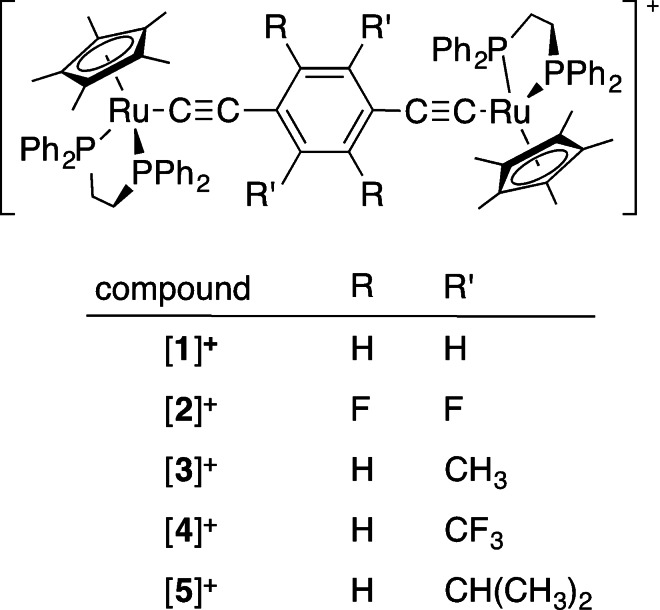
The monocationic, bimetallic half‐sandwich ruthenium mixed‐valence complexes used in this work.

In seeking to extend our understanding of the role of conformation on mixed‐valence systems, and to explore methods to bias these in order to exert a degree of control over the underlying electronic structures, we have designed a series of diethynylbenzene‐bridged bimetallic ruthenium complexes featuring substituents on the central phenylene ring with different steric and electronic properties (Scheme 1). An extensive series of DFT calculations is used to explore the low‐lying structures and associated electronic potential energy surface of the mixed‐valence complexes generated by one‐electron oxidation of these compounds, and the results are confirmed by spectroscopic analysis using samples generated by spectroelectrochemical methods.

## Results and Discussion

The parent compound [{Ru(dppe)Cp*}_2_(μ‐C≡CC_6_H_4_C≡C)]^+^ ([**1**]^+^) provides a convenient reference point from which to begin considerations of the structural and electronic changes introduced by substituents to the central phenylene ring.^[8]^ Whilst the distribution of conformational minima and the associated electronic structures at the BLYP35/def2‐SVP/COSMO(CH_2_Cl_2_) level of theory have been described in detail elsewhere,^[6e]^ this work has introduced additional dispersion corrections (BLYP35‐D3/def2‐SVP/COSMO(CH_2_Cl_2_)). It is useful to the present discussion to briefly recap and refine the general features of [**1**]^+^ at this level of theory.

**Figure 4:**
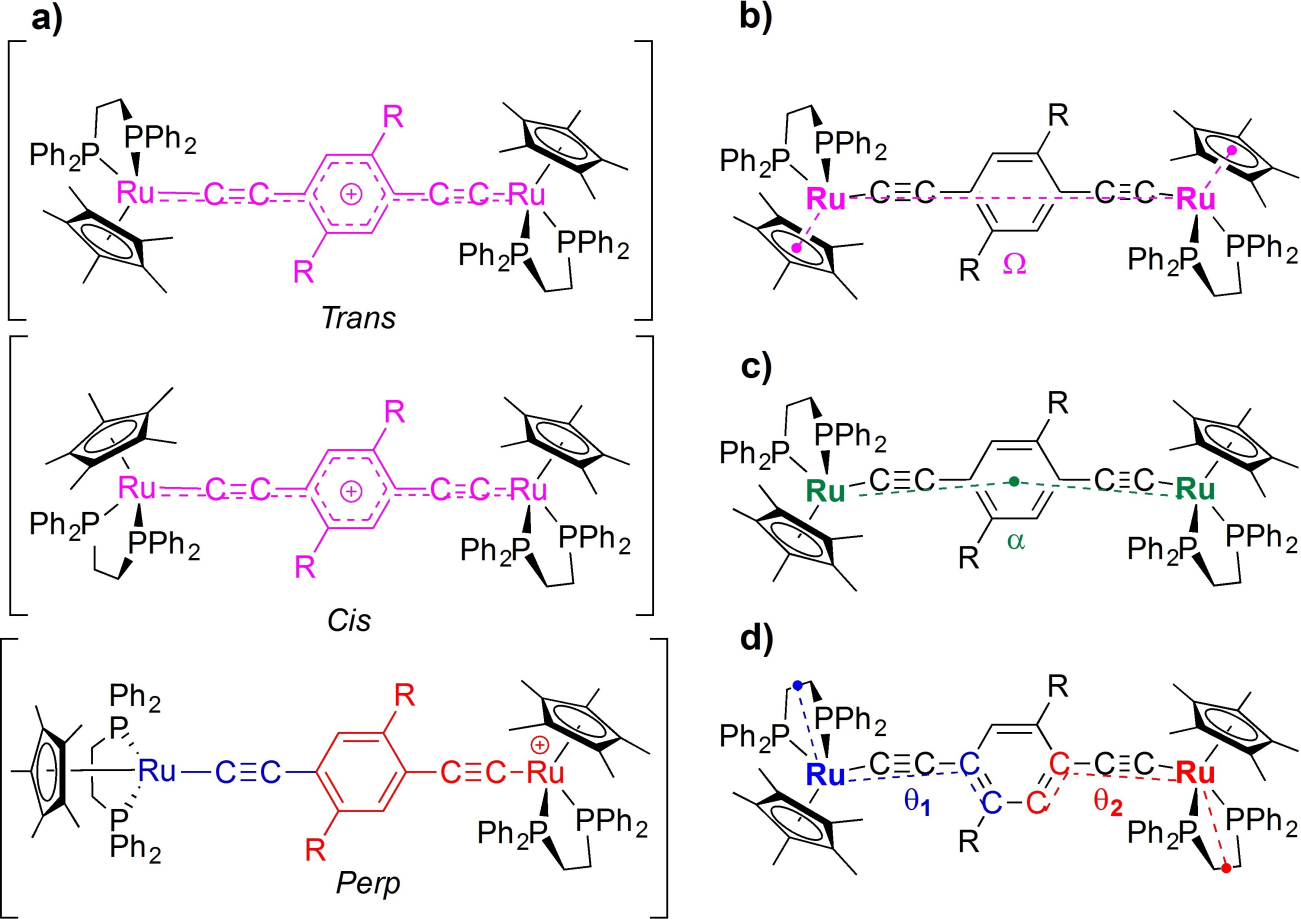
Sketches indicating a) the principal distinctions in the molecular conformers of [**1**–**5**]^+^ and defining the angles b) Ω, c) α, d) θ_1_ and θ_2_.

The ground state potential energy surface of [**1**]^+^ was sampled at the BLYP35‐D3/def2‐SVP/COSMO(CH_2_Cl_2_) level using optimized structures obtained from starting points chosen to model various relative orientations of the half‐sandwich metal fragments and the plane of the phenylene ring in the bridging ligand (Table S1).^[6e]^ The lowest energy (fully optimized) structure of [**1**]^+^ has the central phenylene ring oriented in a ‘vertical’ position (θ_1,2_ near 0°) so that the plane of the ring almost bisects the P−Ru‐P angle of the half‐sandwich fragments (Figure 4, Table 1). This lowest energy conformation has a *cis*‐like arrangement of the half‐sandwich fragments (Ω=2.8°) and is stabilized by dispersion interactions between the central phenylene ring and the aryl moieties of the ancillary dppe ligands. Dispersion interactions have often proven important in stabilizing the conformers explored here, many of which are not identified in calculations that do not allow for such interactions. The spin‐density in this lowest‐energy, *cis*‐like conformer of [**1**]^+^ is distributed almost equally over both the metal centers (Ru1 23 %, Ru2 23 %) and the bridging ligand (50 %), and the system is well described as being ‘fully delocalized’ (Table 1). The most *trans*‐like isomer lies less than 10 kJ/mol higher in energy, also has a ‘vertically’ oriented phenylene ring (Ω=179.3°; θ_1_=0.6°; θ_2_=−0.6°) and is also fully delocalized (Ru1 23 %, Ru2 23 %, bridge 49 %, Table 1).

**Table 1:** Summary of the spin density distributions, relative energies, and computed IR bands over low energy conformers of [1–5]^+^.^[a]^

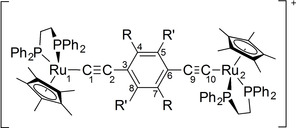																
					spin density distribution									computed IR bands		
	Ω^[b]^	θ_1_^[b]^	θ_2_^[b]^	rel E	Ru1	C1	C2	Ar	R	C9	C10	Ru2	ω^[c]^	ν(C≡C)_s_^[d]^ (rel. int.)	ν(C≡C)_a_^[e]^ (rel. int.)	ν(CC)_ar_^[d]^ (rel. int.)
				kJ/mol										cm^−1^	cm^−1^	cm^−1^
[**1**]^+^	2.8	−1.3	3.9	0.0	23	6	9	19		9	7	23	1.0	2013 (0)	1978 (100)	
	29.0	2.8	−31.9	1.8	28	5	12	19		6	9	17	1.6	2012 (0)	1975 (100)	1580 (1), 1507 (2)
	38.2	13.6	21.8	0.5	23	6	10	21		7	8	21	1.1	2011 (5)	1966 (100)	
	92.3	−0.5	−87.3	13.6	42	−2	24	16		−1	12	6	7.0	1966 (59)	2040 (100)	1556 (75)
	143.0	3.3	23.6	6.1	25	6	10	20		7	8	19	1.3	2014 (0)	1974 (100)	1587 (1)
	153.8	7.7	−43.9	6.6	33	2	15	21		2	12	12	2.7	1989 (100)	1993 (60)	1570 (33)
	155.1	9.8	−44.6	6.6	34	1	15	21		2	12	12	2.9	1989 (39)	1998 (100)	1568 (35)
	164.3	−11.1	−12.7	3.9	25	6	10	19		7	7	21	1.2	2013 (0)	1976 (100)	1586 (1)
	168.5	1.8	1.9	4.8	22	7	8	20		8	7	22	1.0	2016 (0)	1977 (100)	
	179.3	0.6	−0.6	8.1	23	7	8	19		8	7	23	1.0	2016 (0)	1980 (100)	
[**2**]^+^	25.5	11.9	−52.4	5.4	44	−4	24	10	2	1	9	10	4.4	1938 (100)	2024 (88)	1600 (11), 1594 (47)
	36.0	17.4	15.5	0.0	36	−1	16	15	1	7	5	16	2.2	1969 (100)	2007 (25)	1614 (29)
	138.2	−18.7	−38.9	1.1	53	−6	25	9	1	0	8	7	7.3	1934 (100)	2039 (54)	1591 (54)
	169.7	−5.8	−12.8	5.5	28	2	13	15	1	10	3	25	1.1	2007 (7)	2013 (100)	1626 (1)
	170.8	3.5	−0.4	8.3	27	2	12	15	1	11	3	25	1.1	2011 (10)	2015 (100)	1625 (0)
	172.1	1.1	1.0	8.1	27	2	12	15	1	11	2	26	1.0	2011 (0)	2016 (100)	1625 (0)
	172.2	−3.4	−17.9	4.3	28	1	13	15	1	9	4	24	1.2	2003 (3)	2007 (100)	1626 (2)
[**3**]^+^	−173.9	29.6	−28.8	9.7	28	5	11	21	0	5	10	17	1.6	2016 (0)	1947 (100)	
	−159.5	−20.0	30.6	9.8	22	8	8	22	0	7	9	20	1.1	2021 (0)	1967 (100)	
	−158.6	6.4	9.1	3.9	21	8	7	22	0	7	8	21	1.0	2021 (0)	1973 (100)	
	−115.1	9.9	38.8	9.0	24	6	11	22	0	4	11	16	1.5	2013 (7)	1970 (100)	1595 (3)
	−96.5	38.0	37.9	10.4	21	9	7	23	0	7	9	20	1.0	2022 (0)	1964 (100)	
	−32.8	13.6	−44.6	4.1	28	5	12	21	0	4	12	13	2.1	2013 (2)	1981 (100)	1590 (7)
	41.7	13.0	24.6	0.0	23	6	10	23	0	5	10	18	1.2	2014 (4)	1964 (100)	1596 (1)
	62.6	17.7	41.7	7.2	27	5	12	21	0	4	11	15	1.8	2015 (2)	1978 (100)	1590 (5)
	119.4	12.9	−81.2	13.9	37	0	20	21	0	−1	14	7	5.5	1981 (23)	2014 (100)	1574 (35)
	125.9	−24.0	−41.2	14.9	33	2	15	22	0	1	12	12	2.9	1996 (2), 1969 (100)^[e]^		1574 (20)
	146.2	−24.5	−17.7	2.1	20	10	6	23	0	7	10	19	1.1	2025 (0)	1964 (100)	
	155.2	12.0	−47.8	8.3	29	3	14	22	0	2	13	12	2.3	2004 (26)	1982 (100)	1588 (10)
	160.2	2.1	−28.7	5.7	32	3	13	20	0	3	11	14	2.3	2003 (0)	1986 (100)	1586 (9), 1581 (2)
[**4**]^+^	−169.7	1.6	1.8	6.2	27	2	12	14	0	12	2	26	1.1	1997 (3)	2011 (100)	
	−119.2	10.8	34.5	0.0	36	0	20	13	0	5	7	16	2.3	1963 (74)	2016 (100)	1591 (28)
	−64.0	−17.3	−47.8	5.9	46	−4	24	10	0	3	8	10	4.4	1929 (100)	2026 (53)	1574 (41)
	−35.6	1.0	−35.0	1.2	35	−1	19	13	0	6	7	17	2.1	1974 (78)	2014 (100)	1594 (24)
	27.1	−3.5	29.4	1.9	31	0	16	14	0	8	5	20	1.6	1989 (55)	2011 (100)	1600 (10)
	33.2	4.8	32.5	1.1	34	0	20	13	0	5	8	16	2.1	1959 (50)	2014 (100)	1592 (20)
	99.2	−34.1	−34.3	4.2	25	4	12	14	0	12	4	25	1.0	2004 (5)	2008 (100)	
	122.7	−22.6	−42.0	7.1	54	−7	26	9	0	0	8	7	7.6	1922 (100)	2019 (39)	1575 (39)
	147.7	−4.0	−37.3	10.7	43	−3	24	11	0	2	9	11	3.8	1939 (100)	2022 (63)	1577 (35)
	164.6	−5.4	−19.3	9.1	36	−1	20	12	0	6	6	18	2.0	1962 (100)	2012 (69)	1592 (33)
[**5**]^+^	−165.4	5.4	5.5	0.2	20	9	7	23	0	7	9	20	1.0	2028 (0)	1971 (100)	
	−148.9	11.5	26.0	0.0	23	8	10	22	0	4	12	16	1.5	2024 (0)	1963 (100)	
	−132.6	15.0	41.1	1.8	28	5	13	22	0	2	12	13	2.1	2011 (2)	1973 (100)	1583 (3), 1579 (4)
	−68.9	−17.2	−46.9	6.0	27	6	13	22	0	3	13	13	2.1	2019 (4)	1982 (100)	1586 (6),
	−62.0	13.4	−76.8	16.5	37	0	19	21	0	−1	14	7	5.5	1989 (37)	2014 (100)	1567 (43)
	−14.9	15.7	−27.3	6.0	23	8	9	23	0	5	12	16	1.5	2025 (0)	1963 (100)	1591 (2)
	28.4	−15.0	41.5	6.1	25	7	11	22	0	3	12	15	1.7	2022 (3)	1975 (100)	1589 (4), 1581 (1)
	34.7	19.1	13.6	1.9	21	8	7	22	0	7	10	19	1.1	2025 (0)	1968 (100)	
	104.6	11.2	−89.9	12.4	40	−1	21	19	0	−1	13	6	6.7	1979 (44)	2024 (100)	1566 (49)
	165.5	10.7	−41.2	11.1	29	4	13	21	1	3	12	13	2.2	2010 (14)	1982 (100)	1584 (5), 1580 (3)

[a] Properties calculated at BLYP35‐D3/def2‐SVP/COSMO (CH_2_Cl_2_)‐level; [b] Ω and θ_1,2_ as defined in Figure 4; [c] ω is defined as the ratio of computed spin densities on the two metal centers; [d] coupled ν(C≡C) modes (a)=asymmetric acetylene stretch, (s)=symmetric acetylene stretch, (ar)=symmetric arene stretch; [e] independent oscillators.

In addition to these delocalized *cis*‐ and *trans*‐like structures with small θ_1_ and θ_2_ angles, other low energy structural minima can be identified on the ground state potential energy surface of [**1**]^+^ (Table 1). As the half‐sandwich metal fragments adopt more mutually perpendicular arrangements (defined by angles Ω near 90°) and/or the phenylene ring rotates out of conjugation with one or both metal centers (θ_1,2_>±25°), the spin density becomes more localized on one of the metal centers (Table 1).^[6e]^ However, it is important to note that although the metal spin‐density varies across the conformational population, the bridging 1,4‐diethynylbenzene ligand carries a substantial proportion of the overall spin density in all conformers of [**1**]^+^ (ca. 50 %, Table 1). This is entirely in keeping with the significant acetylide ligand involvement in the oxidation of electron‐rich ruthenium acetylide complexes.^[9]^

**Take home message 1**: *The spin density distribution in the low energy minima of [**1**]* ^*+*^ *is sensitive to the molecular conformation, varying from delocalized to strongly localized as a function of the relative orientation of the metal fragments (Ω) and plane of the phenylene bridge (θ_1_*, *θ_2_)*.

The role that substituents on the bridging ligand can play in tuning the electronic character of mixed‐valence complexes has attracted interest from several research groups.[^8b^, ^10^] In an effort to explore electronic effects as a method through which to bias the distribution of spin density within mixed‐valence complexes derived from the parent structure [**1**]^+^, the influence of perfluorination of the central phenylene ring on the physical and electronic structure was explored. A previous study of [{Ru(dppe)Cp*}(μ‐C≡CC_6_F_4_C≡C)]^+^ ([**2**]^+^) based on analysis of the NIR spectra and the DFT (B3LYP/3‐21G*) calculations on a single conformer that were possible at that time could not identify significant differences in the electronic structure and mixed‐valence characteristics compared with [**1**]^+^.^[8a]^ In the present context, the appreciation of the role that molecular conformers may play in the more precise description of mixed‐valence compounds,[^5b^, ^6c^, ^6d^, ^6e^, ^6f^] together with the availability of computational methods better suited to the analysis of compounds with localized electronic structures,^[11]^ prompts this re‐investigation.

The ground state potential energy surface of [{Ru(dppe)Cp*}_2_(μ‐C≡CC_6_F_4_C≡C)]^+^ ([**2**]^+^) was surveyed using the same sampling methods described for [**1**]^+^ (Table 1). The lowest energy conformer of [**2**]^+^ identified in this manner features a structure similar to the lowest energy structure of [**1**]^+^, albeit with the metal fragments twisted out of an idealized *cis* arrangement (Ω=36.0°), and the tetrafluorophenylene ring somewhat offset from bisecting the P−Ru−P angles of each metal fragment (θ_1_=17.4°; θ_2_=15.5°) (Table 1). In contrast to [**1**]^+^, this lowest energy *cis*‐like isomer of [**2**]^+^ features a distinctly asymmetric distribution of spin density over the two metal fragments (Ru1 36 %, Ru2 16 %) (Table 1).

The influence of molecular conformation on the electronic structure of the MV systems can be seen in the *trans*‐like conformers of [**2**]^+^ (169.7°<Ω<172.2°), with the tetrafluorophenylene ring approximately bisecting the metal P−Ru−P angles (0°<θ_1,2_<20°). These conformers lie less than 10 kJ/mol higher in energy than the Ω=36.0° conformer, but exhibit a more symmetric distribution of the spin density over the metal centers (Table 1). Unsurprisingly, low energy conformational minima that arise from rotation of one or both of the metal fragments and/or the tetrafluorophenylene ring out of conjugation also give rise to spin density distributions that are substantially localized towards one metal center (e. g. Ω=25.5°, θ_1_=11.9°, θ_2_=−52.4°, Ru1 10 %, Ru2 44 %; Ω=138.2°, θ_1_=−18.7°, θ_2_=−38.9°, Ru1 7 %, Ru2 53 %) (Table 1).

In contrast to [**1**]^+^, the lower energy conformers of [**2**]^+^ are those with the most localized electronic structures; however, each of the minima of [**2**]^+^ identified are found within in a relatively small range of energies above the lowest energy structure (<10 kJ/mol, Table 1). It is therefore likely that, as with [**1**]^+^, samples of [**2**]^+^ studied in fluid solution at ambient temperature will feature evidence of both the localized and delocalized conformers. Regardless of conformation or electronic structure, the distribution of spin density on the bridging ligand in conformers of [**2**]^+^ (ca. 40 %) is lower than in [**1**]^+^, likely a consequence of the decreased ability of the C_6_F_4_ ring to support the hole and unpaired electron formed on oxidation.

**Take home message 2**: *The perfluorinated phenylene ring in the bridging ligand of [**2**]* ^*+*^ *supports less spin density than the analogous C_6_H_4_ ring in [**1**]^+^, leading to stabilization of conformers with more metal ‘localized’ mixed‐valence character*.

Given the sensitivity of the electronic structure of [**1**]^+^ and [**2**]^+^ to molecular conformation, the influence of more sterically encumbering groups on the central arylene ring was next explored. The potential energy surface of the complex [{Ru(dppe)Cp*}_2_(μ‐C≡CC_6_H_2_‐2,5‐Me_2_‐C≡C)]^+^ ([**3**]^+^) features a large number of minima within 15 kJ/mol of the lowest energy structure (Table 1). As is the case for [**1**]^+^ and [**2**]^+^, these low energy structures of the diethynylxylyl‐bridged complex [**3**]^+^ encompass examples that exhibit electronic structures ranging from largely localized to more significantly delocalized systems. However, regardless of the conformation of the metal fragments (i. e. independent of Ω, θ_1,2_) the electron‐donating character of the methyl substituents results in the bridging ligand of [**3**]^+^ supporting 50–56 % of the spin density (Table 1).

The lowest energy minimum of [**3**]^+^ identified here (Ω=41.7°, θ_1_=13.0°, θ_2_=24.6°) features half‐sandwich fragments mutually positioned around the long molecular axis in a way that deviates from an idealized *cis*‐conformation (for which Ω=0°) and the phenylene ring is also rotated away from the structure which promotes the most favorable conjugation with the metal fragments, as indicated by θ_1,2_>0°. These structural distortions arise from the steric influence of the methyl groups, and the structures are stabilized by C−H.π dispersion forces between the methyl groups and the aryl rings of the dppe ligands. As a result of the relatively poor orbital overlap between the metal 4*d* and ligand π orbitals in this conformation, there is a small degree of asymmetry in the ground state spin distribution in this conformer (Ru1 18 %, Ru2 23 %; Table 1). A low‐lying minimum with a more *trans*‐like conformation in which the xylyl ring approximately bisects the P−Ru−P angles at each metal center offers a more symmetric electronic structure (Ω=−158.6°, θ_1_=6.4°, θ_2_=9.1°, Ru1 21 %, Ru2 21 %).

As the metal fragments in [**3**]^+^ rotate towards more perpendicular orientations (e. g. Ω=−115.1°, θ_1_=9.9°, θ_2_=38.8°), or the xylyl ring rotates out of conjugation with one of the metal centers (e. g. Ω=−32.8°, θ_1_=13.6°, θ_2_=−44.6°), or both scenarios play out in the same conformer (e. g. Ω=−119.4°, θ_1_=12.9°, θ_2_=−81.2°), the energy of the structures increases and the metal spin density asymmetry becomes more pronounced. An interesting minimum with the metal fragments lying almost perpendicular with respect to each other (Ω=−96.5°) yet presenting a rather symmetric distribution of metal spin density can also be identified. Due to the Karplus‐like *cosine* dependence of the orbital overlap along the Ru−C≡C−Ar−C≡C−Ru backbone, the particular orientation of the arylene ring in this conformer with respect to the metal fragments (θ_1_=37.9°; θ_2_=38.0°) permits a degree of electron delocalization between the metal centers.

**Take home message 3**: *The steric influence of the 2,5‐dimethyl groups in [**3**]* ^*+*^ *promotes more conformers with structures that de*via*te from the idealized cis‐ and trans‐like structures and in which the arylene fragment is rotated out of conjugation with the metal centers. However, as a consequence of the electron‐donating nature of the methyl groups (which leads to an increase in the spin‐density localized on the bridging ligand relative to [**1**]* ^*+*^ *and [**2**]^+^), and Karplus‐like orbital overlaps along the backbone of some of these structures (which leads to a degree of electron delocalization despite the twisted conformations of the metal and bridge moieties), there is only a modest asymmetry in the overall electronic structure*.

To combine the electronic and steric concepts introduced in [**2**]^+^ and [**3**]^+^, a complex featuring a 1,4‐diethynyl‐2,5‐bis(trifluoromethyl)benzene bridging ligand ([**4**]^+^) was also explored. For this complex, some 10 distinct conformers were identified, with the metal fragments oriented around the bridging ligand in positions ranging from *trans*‐like through some *perp*‐like minima to *cis*‐like structures. In all conformers of [**4**]^+^ the unpaired spin density residing on the arylene ring of the bridging ligand (40–46 %) is similar to that found in the tetrafluoro derivative [**2**]^+^ (37–43 %), and less than in conformers of [**1**]^+^ (49–51 %) or [**3**]^+^ (50–56 %) (Table 1). The majority of the low‐energy conformers identified offers an asymmetric spin density distribution which is most easily seen through comparison of the spin densities at Ru1 and Ru2 (Table 1). Whilst the majority of minima identified for [**4**]^+^ are considered to have ‘localized’ electronic character, those with the more *trans*‐like disposition of the half‐sandwich metal fragments and also with the bis(trifluoromethyl)benzene ring bisecting the P−Ru−P angles offer the more symmetric electronic environments (e. g. Ω=−169.7°, θ_1_=1.6°, θ_2_=1.8°, Ru1 27 %, Ru2 26 %) (Table 1). An additional minimum with a symmetric distribution of the spin density was also identified, with a similar perpendicular arrangement of the metal fragments (Ω=99.2°) and canted orientation of the arylene bridge (θ_1_=−34.1°; θ_2_=−34.3°) as observed from the protio‐derivative [**3**]^+^ (Table 1).

**Take home message 4**: *The sterically demanding and electron‐withdrawing bis(trifluromethyl) substituents on the phenylene ring of [**4**]* ^*+*^ *stabilize mixed‐valence conformers with more spin density on the metal centers and more localized electronic structures*.

To better define the steric vs. electronic influence of the substituents, complex [**5**]^+^ featuring the rather bulky and moderately electron donating 1,4‐diethynyl‐2,5‐bis(isopropyl)benzene bridging ligand was investigated. The lowest energy structure identified features a modestly asymmetric distribution of spin density at the metal centers, as a by now expected consequence of the poor orbital overlap arising from the relative conformation of the half‐sandwich metal fragments and the plane of the arylene ring (Ω=−148.9°, θ_1_=11.5°, θ_2_=26.0°, Ru1 23 %, Ru2 16 %) (Table 1). However, an almost isoenergetic (ΔE=0.2 kJ mol^−1^) *trans*‐like conformer with symmetric electronic structure is also identified (Ω=−165.4°, θ_1_=5.4°, θ_2_=5.5°, Ru1 20 %, Ru2 20 %). Overall, the steric influence of the isopropyl groups and dispersive and CH.π interactions similar to those observed in conformers of [**3**]^+^ result in the identification of more conformers that deviate from the idealized ‘delocalized’ structures with Ω=0° (*cis*) or 180° (*trans*) and θ_1,2_=0°, whilst the electronic character of the isopropyl groups results in substantial concentration of the spin density on the bridging ligand (51–57 %).

**Take home message 5**: *The sterically demanding and electron‐donating bis(isopropyl) substituents on the phenylene ring of [**5**]* ^*+*^ *bias the population towards structures with ‘perpendicular’ orientations of the half‐sandwich metal fragments and canted bridging phenylene rings. The majority of isomers feature a degree of polarization in their electronic structures with asymmetric spin density at the metal centers, but with a substantial degree of spin density stabilized on the bridging ligand*.

**Scheme 2:**
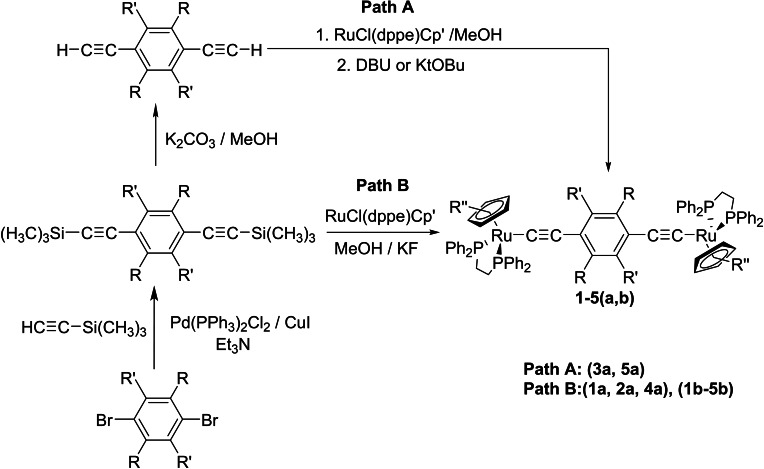
A schematic representation of the synthetic procedures used in the preparation of **1** **a,b**–**5** **a,b**.

To explore the ideas and conclusions drawn from the computational studies, the complexes **1** **a**–**5** **a** were prepared by reaction of RuCl(dppe)Cp* with half an equivalent of either the bis(trimethylsilylethynyl)‐arylene pro‐ligand and KF (**1** **a**, **2** **a**, **4** **a**) or 1,4‐diethynyl‐2,5‐di(alkyl)benzene and DBU (**3** **a**, **5** **a**) (Scheme 2). For further verification of the generality of the conclusions, the Cp analogues **1** **b**–**5** **b** were also prepared from RuCl(dppe)Cp via the KF mediated desilylation‐metallation route (Scheme 2; Supporting Information).

**Figure 5:**
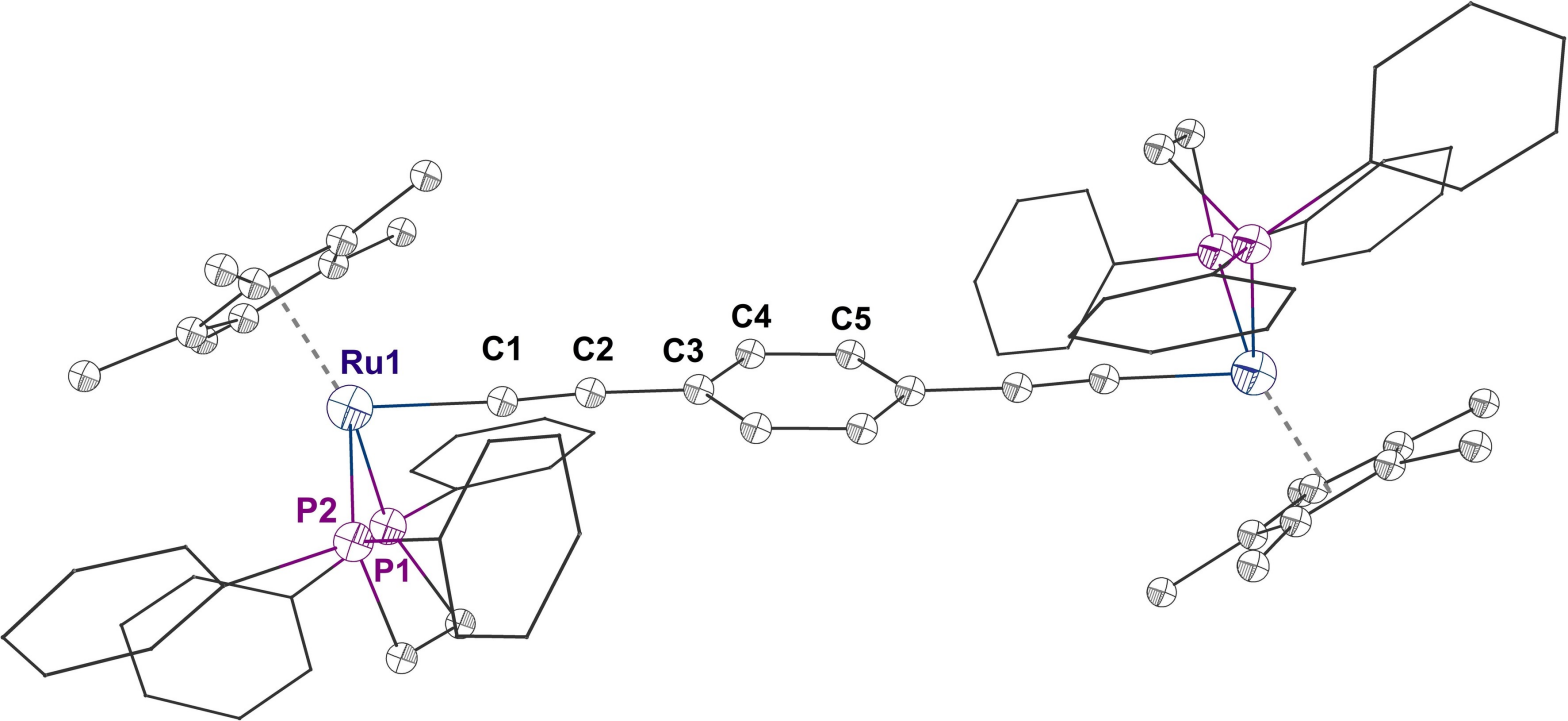
An ORTEP representation of a molecule of **1** **a**, showing the atom labelling scheme.

Single crystals of **1** **a**,**b** (Figure 5, Figure S1), **2** **b** (Figure S1), **3** **a** (Figure S1), **4** **a**,**b** (Figure S1) and **5** **b** (Figure S1) suitable for X‐ray crystallography were readily obtained upon recrystallization (Table 2, Table S2). Together with **2** **a**, the structure of which was reported earlier by Bruce and colleagues,^[8a]^ these compounds are rare examples of crystallographically characterized bimetallic ruthenium complexes of general form [{Cp'(PP)Ru}(μ‐C≡CArC≡C){Ru(PP)Cp'}].[^6a^, ^12^] In each case, the half‐sandwich fragments are disposed in a *trans*‐conformation across the bridging ligand, and related by a center of inversion that renders the two halves of the molecule identical, and imposes Ω=180° and α=180°. The {Ru(dppe)Cp*} and {Ru(dppe)Cp} fragments have the expected structures, whilst the Ru1‐P(1,2), Ru1‐C1, C1‐C2 and C2‐C3 separations are insensitive to the electronic and steric properties of the arylene moiety in the bridge. The arylene moiety sits in a pocket formed by the dppe phenyl rings, and canted away from the idealized ‘vertical’ ( θ=0°) orientation.

**Table 2:** Selected crystallographically determined bond lengths and angles for compounds **1** **a**–**4** **a**, **1** **b**, **2** **b**, **4** **b** and **5** **b**.

	Ru1‐P1	Ru1‐P2	Ru1‐C1	C1‐C2	C2‐C3	Ω^[a]^	θ^[a]^	α^[a]^
**1** **a**	2.253(2)	2.268(2)	2.023(6)	1.177(8)	1.444(8)	180.0	65.7(5)	179.978(5)
**1** **b**	2.2484(13)	2.2653(12)	2.016(5)	1.204(7)	1.436(7)	180.0	88.5(4)^[b]^	179.986(5)
**2** **a** ^[8a]^	2.2542(7)	2.2607(7)	1.996(2)	1.221(3)	1.427(3)	180.0	47.57(16)	180.0
**2** **b**	2.511(6)	2.2686(6)	2.017(2)	1.210(3)	1.428(3)	180.0	84.66(18)^[c]^	179.976(5)
**3** **a**	2.261(1)	2.264(1)	1.997(5)	1.213(7)	1.435(7)	180.0	40.6(5)^[d]^	180.000(5)
**4** **a**	2.260(1)	2.263(1)	2.004(4)	1.203(7)	1.428(7)	180.0	39.0(4)^[d]^	179.977(4)
**4** **b**	2.2568(13)	2.2598(12)	2.005(5)	1.207(7)	1.423(7)	180.0	40.3(5)^[d]^	180.0
**5** **b**	2.2480(8)	2.2561(8)	2.013(3)	1.211(4)	1.446(4)	180.0	69.1(2)^[d]^	179.981(4)

[a] Angles defined in Figure 4; [b] one component of a disordered model; other component 84.1(4)°; [c] one component of a disordered model; other component 77.45(18)°; [d] rotated syn‐clinal towards the substituent.

Each complex **1** **a**,**b**–**5** **a**,**b** exhibited two reversible one‐electron oxidation waves by cyclic voltammetry, the potentials of which were sensitive not only to the nature of the half‐sandwich fragment, but also to the electronic character of the bridging ligand substituents (Table 3). This is consistent with the significant involvement of the acetylide ligand in the oxidation of ruthenium acetylide complexes,^[13]^ and reflected in the spin‐density distributions calculated for the examples described here (Table 1). In all cases, the significant separation of these processes (0.25 V<$|E_{1}^{0}-E_{2}^{0}|$ <0.32 V) indicates the thermodynamic stability of the monocations [**1** **a,b**–**5** **a,b**]^+^ with respect to disproportionation under experimental conditions. Interestingly, both electron‐donating (Me (**3** **a**,**b**), ^i^Pr (**5** **a**,**b**)) and electron withdrawing (F (**2** **a**,**b**), CF_3_ (**4** **a**,**b**)) groups stabilize the monocations relative to the parent system [**1** **a**,**b**]^+^_._

**Table 3:** Summary of electrochemical data from complexes **1** **a**,**b**, **5** **a**,**b**.^[a]^

	E_1_	E_2_	ΔE
**1** **a**	−0.47	−0.22	0.25
**2** **a**	−0.35	−0.03	0.32
**3** **a**	−0.54	−0.23	0.31
**4** **a**	−0.34	−0.02	0.32
**5** **a**	−0.54	−0.22	0.32
**1** **b**	−0.30	−0.07	0.23
**2** **b**	−0.17	+0.10	0.28
**3** **b**	−0.39	−0.13	0.26
**4** **b**	−0.21	+0.08	0.29
**5** **b**	−0.39	−0.12	0.27

[a] Potentials at a platinum disc working electrode are reported vs. ferrocene/ferricenium (0.00 V) from voltammograms recorded in CH_2_Cl_2_ solutions containing 0.1 M NBu_4_PF_6_ as supporting electrolyte. Data are referenced against internal ferrocene, decamethyl ferrocene (E_Fc*/Fc*_^+^=−0.55 V) or acetyl ferrocene (E_FcAc/FcAc_^+^=+0.28 V) calibrants.

**Figure 6:**
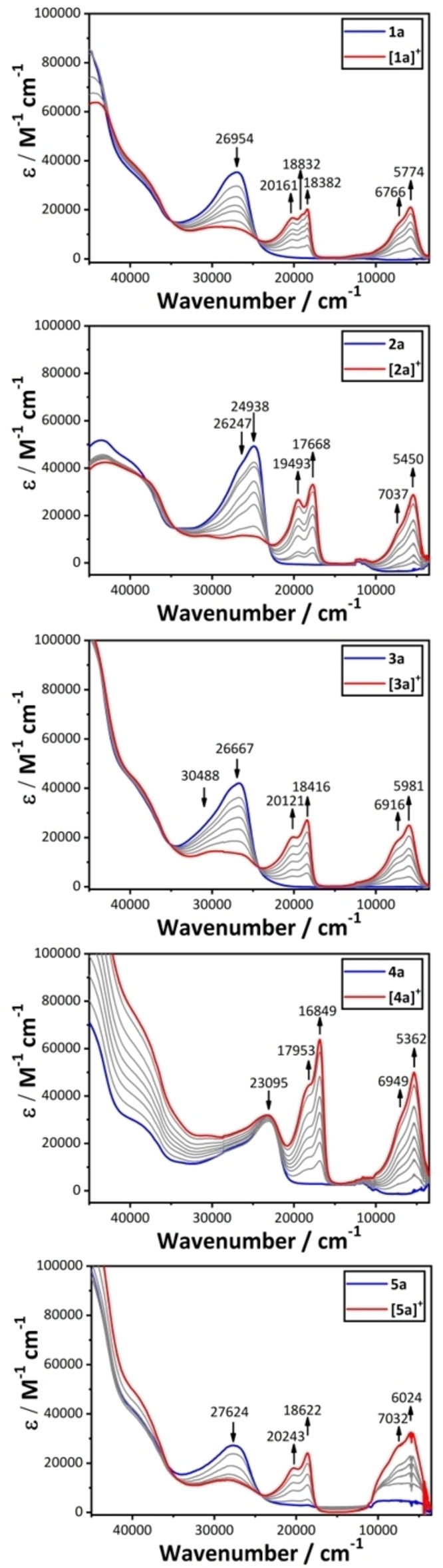
Plots of the UV‐vis‐NIR spectra of [**1** **a**–**5** **a**]^+^ from spectroelectrochemical data.

Selective electrolysis of ca. 1 mM solutions of each of **1** **a**,**b**–**5** **a**,**b** in CH_2_Cl_2_ containing 0.1 M NBu_4_PF_6_ supporting electrolyte in a spectroelectrochemical cell allowed the collection of the UV‐vis‐NIR and IR spectra of [**1** **a**,**b**–**5** **a**,**b**]^+^.^[14]^ The UV‐vis‐NIR spectra of the monocations are characterized by a unique set of structured absorption features between 20,000–16,000 cm^−1^ that resemble the π–π* transitions associated with arylene radicals (Figure 6, Figure S2).^[6a]^ When compared with the spectrum of [**1** **a**,**b**]^+^, this envelope is red‐shifted in the case of the fluoro‐ and trifluoromethyl‐substituted compounds [**2** **a**,**b**]^+^and [**4** **a**,**b**]^+^, reflecting the lower lying π* system. A broad NIR envelope, which spans a wide range of energies below ca. 15,000 cm^−1^ and exhibits several apparent maxima, is also observed. In complexes such as [**1** **a**]^+^ (Figure 6), this NIR absorption envelope has previously been attributed to the multiple IVCT and local dd or interconfigurational bands expected of a classical d^5^‐d^6^ mixed‐valence complex with pseudo‐octahedral metal fragments.[^6a^, ^7^] However, since the low energy electronic transitions in the more delocalized (Class III) structures have essentially π‐π* character, these are far more intense than the true IVCT and localized dd transitions that characterize the most weakly coupled structures. As a consequence, the electronic features of the localized conformers are often overlapped with, and obscured by, the more intense transitions arising from the delocalized structures.^[5b]^ The spectroscopic signatures of conformers with such localized electronic structures are readily overlooked in conventional analyses of NIR band shapes. This has, in turn, prompted suggestions for the greater use of vibrational spectroscopy in the analysis of mixed‐valence systems and assessment of electronic character.[^6b^, ^15^]

More recently, the low energy electronic transitions in a number of representative mixed‐valence complexes have been analyzed with the aid of TDDFT calculations carried out with suitably modified global and local hybrid functionals.[^11b^, ^16^] Here, TDDFT calculations at the BLYP35‐D3/def2‐SVP/COSMO(CH_2_Cl_2_) level of theory on the low‐energy minima described above (Table S3) have been used to explore the NIR band profiles observed experimentally. As similar profiles and trends are apparent in both the {Ru(dppe)Cp*} ([**1** **a**–**5** **a**]^+^) and {Ru(dppe)Cp} ([**1** **b**–**5** **b**]^+^) based series, only compounds [**1** **a**–**5** **a**]^+^ are discussed in detail here, with experimental data from [**1** **b**–**5** **b**]^+^ given in the Supporting Information (Figure S2).

Taking the spectrum [**1** **a**]^+^ as a point of reference,^[6e]^ the principal NIR absorption bands can be assigned by TDDFT calculations with the computational model [**1**]^+^ to the HOMO‐SOMO (or [β‐HOMO]‐[β‐LUMO]) transitions (calculated to fall between 6100–6800 cm^−1^) (Table S3). For the conformers with the most delocalized electronic structures, the HOMO‐SOMO transition has largely π‐π* character (Figure 6, Table S3, Table S4). However, given that the donor orbital (HOMO) in these delocalized conformers is somewhat more metal in composition, and the acceptor orbital (SOMO) features more bridging ligand character, the lowest energy transition might also be described as a metal‐to‐bridge charge transfer (MBCT) band. In contrast the HOMO‐SOMO band in the most localized conformers, which fall to the higher end of the distribution of transition energies, have greater metal‐to‐metal charge‐transfer (or IVCT) or localized dd character. Indeed, the five lowest energy excitations fit well to the Meyer model of multiple IVCT and dd transitions (Figure 6, Table S3, Table S4).^[7]^ In other words, the intense lower energy features in the NIR absorption band envelope of [**1** **a**]^+^ arise from transitions associated with the *cis*‐ and *trans*‐conformers with Class III (delocalized) electronic structures, that might be better termed as charge resonance or MBCT transitions than truly ‘inter‐valence’ transitions. The higher energy, lower intensity shoulders arise from the IVCT bands in the conformers with more classically Class II mixed‐valence electronic structures.

As noted above, the introduction of the electron‐withdrawing tetraflurophenylene in [**2** **a**]^+^ results in a red‐shift of the band envelope relative to [**1** **a**]^+^ (Figure 6), which is also observed in the TDDFT results (Table S3). In the case of [**2** **a**]^+^, the *trans*‐like conformer (Ω=172.1°; θ_1_=1.1°; θ_2_=1.0°) which offers the most delocalized electronic structure (Table 1), also features an intense π‐π* (or MBCT) transition, calculated to fall at 5850 cm^−1^. The two most *cis*‐like conformers (Ω=36.0°; Ω=25.5°) have more asymmetrically distributed spin densities and more localized metal valencies (Table 1). The HOMO‐SOMO transition (calculated at 6001 cm^−1^) in the lowest energy conformer (Ω=36.0°) can be broadly described as having IVCT character, albeit with significant contributions from the bridging ligand, and has appreciable calculated intensity (Table S3). The Ω=25.5° conformer sits slightly higher in energy (ΔE=+5.4 kJ mol^−1^) and has an even more pronounced localization of the spin density due the more significant canting of the tetrafluorophenylene ring (θ_1_=11.9°; θ_2_=−52.4°) (Table 1). The lowest energy transition of this localized conformer falls more clearly within the higher energy shoulder (6607 cm^−1^), and arises from the [HOMO‐3] to SOMO transition with more dd character and much lower calculated intensity (Table S3, Table S4). The HOMO‐SOMO transition (6973 cm^−1^) of this conformer has IVCT character and has higher calculated intensity.

Unsurprisingly, the conformer in which the mutual arrangement of the half‐sandwich metal fragments and phenylene ring is least suited to orbital overlap along the molecular backbone (Ω=138.2°; θ_1_=−18.7°; θ_2_=−38.9°) offers the most localized electronic structure of the minima discussed here (Table 1). The lowest energy transition at 5093 cm^−1^ ([HOMO‐3]‐SOMO) also has dd character, whilst the more genuinely IVCT band is calculated to fall at 7689 cm^−1^ (HOMO‐SOMO).

**Take home message 6**: *Even though the population of [**2*** ***a**]* ^*+*^ *may feature a greater proportion of conformers with localized electronic structures with a smaller degree of spin density on the bridging ligand than [**1*** ***a**]^+^, the involvement of the bridge in both the HOMO and SOMO leads to relatively intense absorptions in the NIR region for both the more localized and delocalized members of the population. This highlights the difficulties in analysis of conformational populations of mixed‐valence complexes by electronic absorption spectroscopy*.

Turning to [**3** **a**]^+^, which features the electron‐donating methyl substituents at the 2,5‐positions of the bridging phenylene ring, the NIR band envelope is modestly blue‐shifted in comparison with [**1** **a**]^+^ but offers a very similar overall profile (Figure 6). Although the spin‐density is not evenly distributed over both Ru centers in the lowest energy, *cis*‐like conformer (Ω=41.7°, θ_1_=13.0°, θ_2_=24.6°) the differences in spin density at the metal centers is small and >50 % of the total spin density rests on the bridging ligand (Table 1). The HOMO‐SOMO transition in this lowest energy conformer is calculated to give rise to an intense absorption at 6868 cm^−1^ with largely π‐π* (or MBCT) character (Table S3, Table S4). Absorptions arising from HOMO‐SOMO transitions falling between 6308–7251 cm^−1^ with similar π‐π* character are also predicted from the conformers with Ω=−159.5°, −158.6°, −158.6°, −96.5° and 146.2°. The HOMO‐SOMO transitions in the more localized conformers of [**3** **a**]^+^ (Ω=−173.9, −32.8°, 62.6°, 119.4°, 125.9° 155.2°, 160.2° and −115.1°) lie within the higher energy edge of the band envelope, and are calculated between 5766 and 7384 cm^−1^, respectively. These transitions may all be described as IVCT transitions, but the involvement of the bridge in supporting the spin density reduces the overall charge transfer character.

**Take home message 7**: *The electron donating methyl groups bias the conformational population away from idealized cis and trans structures, but increase the spin density population on the bridge; this latter effect limits the degree of metal character in the SOMO and decreases the overall IVCT character of the lowest energy transitions*.

As with the tetrafluorobenzene bridge in [**2** **a**]^+^, the electron‐withdrawing 2,5‐bis(trifluromethyl) groups in [**4** **a**]^+^ increase the proportion of low‐energy conformers with more localized electronic structures (Table 1, Table S3, Table S4). This collection of localized conformers of [**4** **a**]^+^, which includes the global minimum (Ω=−119.2°), all feature a HOMO‐SOMO transition in the range 5230–7373 cm^−1^ with significant IVCT character (Table S3, Table S4). Due to the significant involvement of the bridging ligand in both the HOMO and SOMO (Table S4) these low energy transitions gain appreciable intensity. In addition, in the case of the most localized structure (Ω=−64.0°), a lower energy, less intense band with significant dd character is also calculated at 5230 cm^−1^. The HOMO‐SOMO transitions arising from conformers with the most delocalized electronic structures (Ω=−169.7° and 99.2°) are more π‐π*/MBCT in character and, as expected, contribute to the low‐energy edge of the NIR band envelope, being calculated at 5881 and 5465 cm^−1^, respectively.

**Take home message 8**: *The electron‐withdrawing bis‐CF_3_ groups in [**4*** ***a**]* ^*+*^ *have a similar effect on the electronic structure of diethynyl benzene bridged bimetallic mixed valence complexes as the four fluorine substituents in [**2*** ***a**]^+^, biasing the population towards more localized systems, with relatively intense IVCT bands*.

TDDFT calculations carried out on the conformers of [**5** **a**]^+^ reinforce the general descriptions outlined above. Whilst the steric bulk of the isopropyl groups distorts the molecular structures away from idealized *cis* or *trans* conformations with low θ_1,2_ angles leading to a degree of localization or polarization in the electronic structure, the electron‐donating nature of the alkyl moieties results in more of the spin density accumulating on the bridge. In all but the most polarized structures (Ω=−62.0° and 104.6°), the lowest energy electron transitions, which fall in the range 6627–7457 cm^−1^, have significant calculated intensity and arise from the HOMO‐SOMO transition with MBCT character. The conformers with the most localized electronic structures feature θ_2_ >75° (Ω=−62.0°, θ_1_=13.4°, θ_2_=−76.8°; Ω=−104.6°, θ_1_=11.2°, θ_2_=−89.9°) and have low energy, low intensity dd bands ([HOMO‐3]‐SOMO) near 6900 cm^−1^, and intense bands with more distinct IVCT character on the high energy side of the NIR band envelope at 8701 and 8954 cm^−1^ (Table S3, Table S4).

**Take home message 9**: *The steric properties of the isopropyl groups bias the low energy conformations of [**5*** ***a**]* ^*+*^ *towards structures with a degree of polarization in the ground‐state electronic structures. However, the electron‐donating character of the substituents results in a significant degree of bridge oxidation leading to electronic transitions with MBCT character in all but the most polarized conformers*.

**Figure 7:**
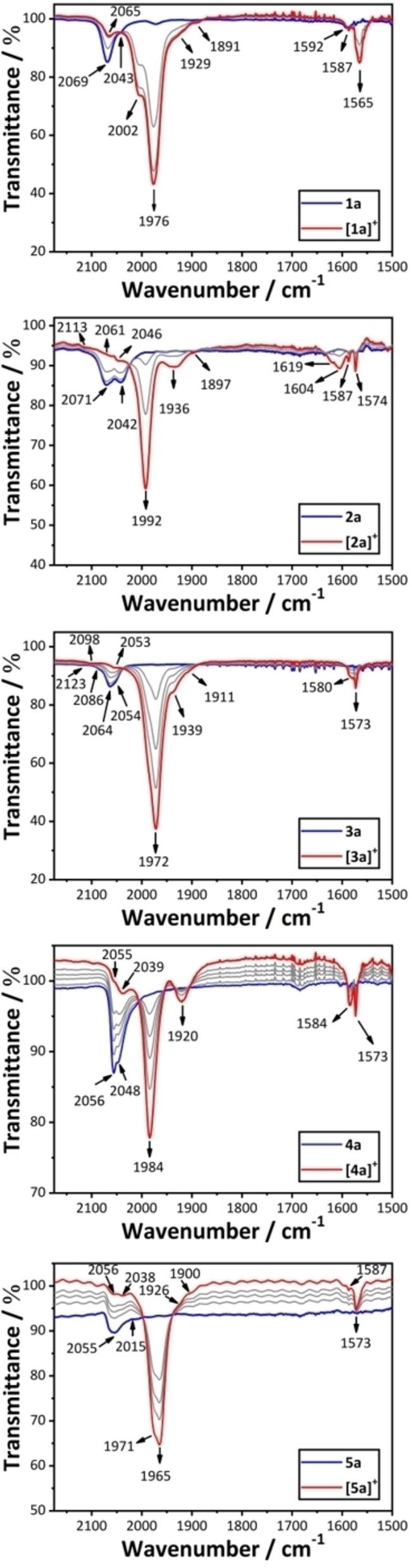
Plots of the spectroelectrochemically generated IR spectra of [**1** **a**–**5** **a**]^+^.

Infra‐red spectroscopy has been identified as an alternative tool through which to probe the electronic structure of mixed‐valence complexes, taking advantage of the unique combination of selection rules and timescales associated with this method.[^15a^, ^15b^, ^15c^, ^15d^, ^15g^, ^15h^, ^15i^, ^17^] For example, the IR spectrum of [**1** **a**]^+^ contains a rather intense band envelope with apparent maxima at 2002 and 1976 cm^−1^ (Figure 7, Table 1). The higher frequency feature is attributed to the symmetric and asymmetric bridge stretching modes of similar energy arising from modestly polarized structures (Ω=153.8°, θ_1_=7.7°, θ_2_=−43.9°; ν(C≡C)_asym_ 1993 and ν(C≡C)_sym_ 1989 cm^−1^; (Ω=155.1°, θ_1_=9.8°, θ_2_=−44.6°; ν(C≡C)_asym_ 1998 and ν(C≡C)_sym_ 1989 cm^−1^) (Table S5). The lower energy feature arises from the asymmetric ν(C≡C) stretches of the conformers with more delocalized electronic structures (Table 1).^[6e]^

The conformers of [**1** **a**]^+^ with the more localized electronic structure give rise to a distinct two band pattern of ν(C≡C) bands arising from both the asymmetric and symmetric stretching modes (e. g. [**1**]^+^ Ω=92.3°, θ_1_=−0.5°, θ_2_=−87.3°; ν(C≡C)_asym_ 2040 and ν(C≡C)_sym_ 1980 cm^−1^) and the aryl‐ring ν(C=C) mode (ca. 1560 cm^−1^). The symmetric ν(C≡C) and aryl ring bands gain intensity due to the electronic asymmetry of the localized conformers over the long molecular axis.

**Take home message 10**: *The electronic character of the conformers is reflected in the ν(*C≡C*) and ν(C=C) modes of the bridging ligand*.

To provide a further link between the electronic and steric effects of the aryl ring substituents on the electronic structure of the mixed‐valence complexes being explored here, the calculated vibrational frequencies from the low‐energy minima of [**2**–**5**]^+^ were compared with the mid‐IR spectra collected from the experimental systems [**2** **a**–**5** **a**]^+^ using spectroelectrochemical methods (Figure 7, Table S6).

In the case of the 1,4‐diethynyl tetrafluorophenyl‐bridged complex [**2**]^+^, the conformers with the most localized electronic structures (Ω=25.5°, 36.0°, 138.2°) are computed to give IR active symmetric (1934–1969 cm^−1^) and asymmetric (2007–2039 cm^−1^) ν(C≡C) bands (Table S5). These correspond to features in [**2** **a**]^+^ near 1936 and 2046 cm^−1^, respectively (Figure 7). The more *trans*‐like conformations of [**2**]^+^ (Ω=169.7°, 170.8°, 172.1°, 172.2°) have the most delocalized electronic structures, and the calculated IR spectra are each dominated by a single, asymmetric ν(C≡C) band, which collectively fall in a narrow range of frequencies (2007–2016 cm^−1^) (Table S5). This result neatly fits the observation of a relatively strong and sharp ν(C≡C) band in the experimental IR spectrum of [**2** **a**]^+^ at 1992 cm^−1^ (Figure 7).

A series of weaker stretching modes of the tetraflurophenylene ring are calculated in conformers of [**2**]^+^ between 1599–1626 and 1581–1596 cm^−1^. These bands, which only gain significant intensity in the more polarized conformers, correspond well to the weak, broad feature near 1600 cm^−1^ and the much sharper band at 1574 cm^−1^ in the spectrum of [**2** **a**]^+^. In turn, for each of these regions, the lower frequency bands gain greatest intensity in the conformers with the most localized electronic structures (i. e. Ω=25.5°, 138.2°).

The general features of the IR spectra described for the 1,4‐diethynyl‐tetrafluorobenzene‐bridged complex [**2**]^+^/[**2** **a**]^+^ are also found in both the computational and spectroelectrochemically generated spectra of the 1,4‐diethynyl‐2,5‐bis(trifluoromethyl)phenylene analogues [**4**]^+^ and [**4** **a**]^+^. The majority of conformers of [**4**]^+^ identified in this study exhibit relatively localized electronic structures, which give rise to symmetric (1922–1989 cm^−1^) and asymmetric (2008–2026 cm^−1^) ν(C≡C) bands (Table 1). The most polarized conformers (Ω=−64.0°, θ_1_=−17.3°, θ_2_=−47.8°; Ω=122.7°, θ_1_=−22.6°, θ_2_=−42.0°; Ω=147.7°, θ_1_=−4.0°, θ_2_=−37.3°) appear to account for the less intense bands observed at 1920 cm^‐1^ and 2039 cm^‐1^ in samples of [**4** **a**]^+^, while the somewhat less polarized conformers contribute mostly to the most intense band at 1984 cm^‐1^ (Figure 7).

The aryl ring of the bridging ligand in [**4**]^+^ gives rise to a series of ν(C=C) bands clustered around 1591–1600 cm^−1^, but falling to lower frequency (1574/1575 cm^−1^) in the three most strongly polarized conformers (Ω=−64.0°, θ_1=_−17.3°, θ_2=_−47.8°; Ω=122.7°, θ_1_=−22.6°, θ_2_=−42.0°; Ω=147.7°, θ_1_=−4.0°, θ_2_=−37.3°). Building on this observation, the intensities and frequencies of these aryl ring stretches have proven diagnostic of the differing concentration of the electron hole on the metal centers and the bridge in the delocalized and localized conformers (Table S4). The parameter ω, defined here as the ratio of computed spin densities on the two metal centers, encodes the polarization of a given conformer. For complexes [**1**]^+^, [**3**]^+^, and [**5**]^+^ the aryl stretches gain appreciable intensity in conformers where this polarization factor ω exceeds a value of 2.5; for [**2**]^+^, and [**4**]^+^ this threshold is 2.0.

For the more delocalized conformers, the greater proportion of positive charge on the bridge leads to a small contraction of the bonds within the aryl ring as a result of the significant aryl‐ring anti‐bonding character of the SOMO. The consequently stronger bonding character in the aryl ring leads to somewhat higher ring stretching frequencies in the less polarized conformers. Conversely, the more metal‐based oxidation processes that characterize the more polarized structures with more substantially localized electronic structures has less impact on the bonding in the arylene ring system which leads to relatively lower associated vibrational frequencies.

**Take home message 11**: *Substituents that induce a preference for more polarized conformers, like fluorine or CF_3_ groups, generate richer IR spectra of the MV radical cations, both* via *the ν(*C≡C*) and ν(C=C) modes of the bridging ligand. The most polarized structures with the most localized electronic structures are characterized by intense, relatively low frequency, aryl ν(C=C) bands*.

The conformers of the alkyl substituted model complexes [**3**]^+^ and [**5**]^+^ are generally less polarized than those of [**2**]^+^ and [**4**]^+^ (Table 1). In all but the most polarized structures, only the asymmetric ν(C≡C) band has appreciable calculated intensity, and it falls in a narrow range of frequencies ([**3**]^+^: 1964–1981 cm^−1^; [**5**]^+^ 1963–1981 cm^‐1^) (Table S5). For the conformers with the most polarized ground state electronic structures (Ω=−62.0°, θ_1=_13.4°, θ_2=_−76.8°; Ω=104.6°, θ_1=_11.2°, θ_2=_−89.9°) the symmetric ν(C≡C) band also gains intensity, giving rise to a two band pattern (2014/1989 cm^−1^ and 2024/1979 cm^−1^, respectively) together with an IR active stretch associated with the central phenylene ring at 1567 or 1566 cm^−1^ in each case which is at lower frequency than the weak bands from the other conformers near 1580–1590 cm^−1^.

**Take home message 12**: *The alkyl substituents in the 2,5‐positions of the bridging ligand favor delocalized structures with greater bridging ligand character supporting the unpaired electron, giving rise to simpler IR spectra with strong asymmetric ν(*C≡C*) bands and weaker ν(C=C) aryl ring breathing modes*.

The electronic and steric factors associated with substituents on the diethynylarylene bridge can be used to exert a degree of control over the electronic character of populations of mixed‐valence complexes. In a similar vein, Lapinte and colleagues have shown that the solid state IR spectra of samples of the mixed‐valence complex [Cp*(dppe)Fe}(μ‐py’)Fe(dppe)Cp*}]PF_6_ (py’=3,5‐diethynyl pyridine) were sensitive to the temperature at which the sample was precipitated.^[5a]^ Those differences were attributed to difference in the population of conformers of the pyridyl ring trapped in the solid state. We have adopted Lapinte's procedure and examined the IR spectra of solid samples of [**1** **a**]^+^ prepared by chemical oxidation (AgPF_6_) and precipitation at room temperature and −78 °C by way of example.

The gross appearance of the IR spectra of spectroelectrochemically generated [**1** **a**]^+^ in CH_2_Cl_2_/0.1 NBu_4_PF_6_ solution (Figure 7), and chemically prepared and isolated samples of [**1** **a**]PF_6_ in pure CH_2_Cl_2_ solution, and as solid samples precipitated at room temperature or −78 °C are similar with prominent ν(C≡C) features at ca. 2060, 1970 and the arene ν(C=C) breathing modes between 1600–1550 cm^−1^ (Figure S14). However, spectra of the solid samples feature generally sharper vibrational bands, with fewer pronounced shoulders, consistent with a decrease in the number of thermally populated conformers.

In the solid‐state sample precipitated at −78 °C, the band envelope associated with the asymmetric stretch of the more ‘delocalized’ conformers (1968 cm^−1^) has greater relative intensity than the symmetric stretch which is associated with the more localized conformers (ca. 2060 cm^−1^). Of equal interest are the ν(C=C) breathing bands of the bridging arene, which are dominated by the higher frequency modes associated with the more delocalized conformers in spectra from solid state samples precipitated at both room temperature and −78 °C, and which is particularly narrow in the case of the −78 °C sample. It therefore appears that precipitation of [**1** **a**]^+^ at either room temperature or −78 °C traps a smaller range of the conformers than are present in solution (evinced by the reduced number of shoulders to the IR spectra), and that there is a small increase in the relative population of the more delocalized conformers in this reduced population in the −78 °C sample. This is entirely consistent with the nature of the lowest energy structures determined by DFT calculations.

## Conclusion

The electronic structure and mixed‐valence characteristics of complexes [{Cp*(dppe)Ru}(μ‐C≡CC_6_H_4_C≡C){Ru(dppe)Cp*}]^+^ are sensitive to the relative conformation of the half‐sandwich fragments to each other (expressed here by the angle Ω) and the plane of the phenylene ring (denoted by angles θ_1_ and θ_2_). Molecular geometries with *cis* or *trans* arrangement of the metal fragments (Ω=0 or 180°) and the phenylene ring approximately bisecting the P−Ru‐P angles (θ_1,2_=0°) have the most delocalized electronic structures, with approximately half of the unpaired electron spin‐density supported by the bridging ligand. As the metal fragments and/or the phenylene ring rotate around the long molecular axis, the electronic structure becomes increasingly polarized, with greater stabilization of the spin density at one metal center and concomitantly less on the bridge. Consequently, the vibrational and electronic spectra of solutions of [{Cp*(dppe)Ru}(μ‐C≡CC_6_H_4_C≡C){Ru(dppe)Cp*}]^+^ display features characteristic of both Class III and Class II mixed‐valence systems.

It has now proven possible to bias these populations by introducing substituents of different steric and electronic properties to the periphery of the central ring in the 1,4‐diethynylarylene bridging ligand. The introduction of electron withdrawing groups, as is the case in 1,4‐diethynyl‐2,3,5,6‐tetrafluorobenzene, destabilizes conformers with substantial spin density on the bridging ligand, leading to more conformers with ‘Class II’ character in solutions of these complexes.

Bulky substituents, such as the methyl groups in 1,4‐diethynyl‐2,5‐di(methyl)benzene or the isopropyl groups in 1,4‐diethynyl‐2,5‐bis(diisopropyl)benzene, lead to structural distortion away from the *cis*‐ and *trans*‐ structures with phenylene ring aligned for efficient charge delocalization. However, in these cases the electron‐donating alkyl groups help to stabilize spin density on the bridge and the systems are less sensitive to the metal conformation and behave rather more as organic redox systems. In combination, the electron‐withdrawing and sterically demanding CF_3_ groups in 1,4‐diethynyl‐2,5‐bis(trifluoromethyl)benzene lead to stabilization of complexes with structures that offer significant Class II character. Thus, whilst the low barriers to rotation around the Ru−C and ethynyl‐arylene axis lead to population of conformers for all systems explored here, a combination of electronic and steric factors can be used to bias these populations towards the extremes of electronically delocalized (Class III) and localized (Class II) systems.

## Experimental Section

**Computational methods**: All calculations regarding the structure optimization and the property analyses (harmonic vibrational frequencies, electronic excitations, spin density distribution) were performed with the global hybrid functional BLYP35, using the TURBOMOLE (TBM) software package (version 6.4), modified by the Berlin group.^[18]^ The BLYP35 functional is a global hybrid version of the BLYP functional admixed with 35 % Hartree‐Fock (HF) exchange,^[19]^ and was established predominantly for organic mixed‐valence compounds.[^11a^, ^20^] It is suited well to model compounds that lie within the class II/III borderline of the Robin‐Day scheme,^[7]^ and the applicability of BLYP35 to studies of mixed‐valence transition metal complexes has been demonstrated.[^5b^, ^6b^, ^6c^, ^6e^, ^6f^, ^21^] In order to account for the strong intramolecular dispersion forces, Grimme's D3 correction^[22]^ is added to the BLYP35 functional with parameters r_s,6_=1.1225 and s_8_=0.9258.^[23]^ Additionally, the continuum solvent model COSMO was used to mimic the solvent dichloromethane (ϵ=8.93). Split‐valence basis sets def2‐SVP,^[24]^ and gridsize m5 (grid 3 for SCF iterations and grid 5 for final energy calculation) were used for all calculations. def2‐SVP includes a scalar relativistic pseudopotential for Ru.^[25]^ The SCF energy convergence criterion was set to 10^−8^ E_h_. Excitation energies were computed also at BLYP35‐D3/def2‐SVP/COSMO(CH_2_Cl_2_) level using linear‐response TDDFT.

**General reaction conditions**: All reactions were carried out under an atmosphere of dry nitrogen using standard Schlenk techniques and reaction solvents were sparged with nitrogen. Methanol was dried by distillation from iodine/magnesium turning. Diethylether and tetrahydrofuran were dried on an Inert^TM^ solvent purification system. Diisopropylamine and triethylamine were pre‐dried over potassium hydroxide and distilled from calcium hydride before use and stored under N_2_. Other solvents were standard reagent grade and used as received. No special precautions were taken to exclude air or moisture during workup except where otherwise indicated.

The precursor complexes RuCl(dppe)Cp*,^[26]^, 1,4‐bis(trimethylsilylethynyl)‐2,5‐di(trifluoromethyl)benzene,^[27]^ and complexes **1** **a** and **2** **a** were prepared by literature methods. The ligand precursor 1,4‐diethynyl‐2,5‐dimethylbenzene was prepared by a minor modification of the literature route,^[28]^ as described below. All other reagents were purchased and used as received.

Cyclic voltammetry was carried out using a PalmSens Emstat^3+^ potentiostat, with platinum working, counter and pseudo‐reference electrodes, from solutions in dichloromethane containing 0.1 M NBu_4_PF_6_ as the electrolyte (ν=100, 200, 400 and 800 mVs^−1^). The ferrocene/ferrocinium, decamethylferrocene/decamethylferrocinium (−0.55 V vs. Fc/Fc^+^) or acetylferrocene/acetylferrocinium (+0.26 V vs. Fc/Fc^+^) couples were used as internal references for potential measurements.

Spectroelectrochemistry was conducted in an OTTLE cell,^[29]^ using solutions of the analyte (ca. 3 mM) in CH_2_Cl_2_ containing 0.1 M nBu_4_NPF_6_ as the supporting electrolyte. UV‐Vis‐NIR spectra were recorded on an Agilent Technologies Cary 5000 spectrometer, FTIR spectra were measured on an Agilent Technologies Cary 660 spectrometer or a Nicolet Avatar 360 spectrometer from solutions in dichloromethane in a thin layer cell fitted with CaF_2_ windows or as a solid in Attenuated Total Reflection (ATR) mode. Elemental analyses were performed at the London Metropolitan University. ESI‐MS were recorded on a Waters LCT Premier XE mass spectrometer in positive mode from solutions in acetonitrile. NMR spectra were recorded at 25 °C on a Bruker Avance 600 (^1^H, 600.1 MHz; ^13^C, 151 MHz; ^19^F, 565 MHz; ^31^P, 243 MHz), a Bruker Avance 500 (^1^H, 500.1 MHz; ^13^C, 126 MHz; ^19^F, 471 MHz; ^31^P, 202 MHz) or a Varian Inova 400 (^1^H, 400 MHz; ^13^C, 101 MHz; ^19^F, 377 MHz; ^31^P, 162 MHz) spectrometer using CDCl_3_ or C_6_D_6_ as the solvent. Chemical shifts were determined relative to internal residual solvent signals (^1^H, ^13^C) or external C_6_F_6_ (^19^F; δ=−164.9 ppm) and 85 % H_3_PO_4_ (^31^P, δ=0.0 ppm).

*1,4‐bis((trimethylsilyl)ethynyl)‐2,5‐dimethylbenzene*: ^[28]^ A Schlenk flask was charged with a solution of 2,5‐dibromo‐*p*‐xylene (2.50 g, 9.47 mmol) in triethylamine (20 mL)/THF (10 mL), and treated with PdCl_2_(PPh_3_)_2_ (532 mg, 756 *μ*mol),^[30]^ copper(I) iodide (144 mg, 756 *μ*mol) and ethynyltrimethysilane (3.35 mL, 23.7 mmol). The reaction mixture was subsequently heated at reflux point for overnight. After this time, the solvent was removed under reduced pressure and the residue purified by flash chromatography on silica, eluting with hexanes. The product was further purified by two recrystallizations from hexanes/ethanol to afford the product as a white crystalline solid (1.77 g, 63 % yield). FTIR (ATR) *ν*/cm^−1^: 2153, 2100 (C≡C); ^1^H NMR (600 MHz, CDCl_3_) δ/ppm: 7.26 (s, 2H, C_6_ *H_2_* (CH_3_)_2_), 2.34 (s, 6H, CH_3_), 0.25 (s, 18 H, Si(CH_3_)_3_); ^13^C{^1^H} NMR (151 MHz, CDCl_3_) δ/ppm: 137.7 (C^aryl^‐H in C_6_H_2_(CH_3_)_2_), 132.9 (*C*‐CH_3_, C_6_H_2_(CH_3_)_2_), 123.1 (C^*i*^ in C_6_H_2_(CH_3_)_2_), 104.0 (*C*≡C‐Si), 99.7 (C≡*C*‐Si), 20.0 (CH_3_),0.2 (Si(*C*H_3_)_3_); ESI‐MS *m/z*: found: 298 [M^+^]. ^1^H and ^13^C NMR data were in agreement with those reported.^[28]^

[{Ru(dppe)Cp*}_2_(μ‐C≡C‐1,4‐C_6_H_4_‐C≡C)] **(1** **a)**: Compound **1** was prepared by the literature method,^[8a]^ and crystals suitable for X‐ray measurements were grown by slow diffusion of methanol into a solution of the complex in dichloromethane.

[{Ru(dppe)Cp*}_2_(μ‐C≡C‐1,4‐C_6_H_2_{2,5‐(CH_3_)_2_}‐C≡C)] **(3** **a)**: A mixture of RuCl(dppe)Cp* (200 mg, 299 *μ*mol), 1,4‐diethynyl‐2,5‐dimethylbenzene (23.0 mg, 149 *μ*mol) in methanol (10 mL) was heated at 75 °C for 1 h to give a deep red solution. Heating was discontinued and two drops of DBU were added causing the immediate formation of a yellow precipitate. The precipitate was collected by filtration and washed with methanol, diethyl ether and hexanes (178 mg, 84 %). Recrystallization from hot dichloromethane gave the crystals suitable for X‐ray characterization. FTIR (CH_2_Cl_2_) *ν*/cm^−1^: 2064, 2054 (C≡C); FTIR (ATR) *ν*/cm^−1^: 2072 (C≡C); ^1^H NMR (400 MHz, CDCl_3_) δ/ppm: 7.77 (*t*, ^3^ *J*_HH_=6.3 Hz, 8H, H^*o*^ in C_6_H_5_ ring (dppe)), 7.32‐7.23 (m, 24H, H^*m/p*^ in C_6_H_5_ ring (dppe) (partly obscured with the solvent peak), 7.15 (*t*, ^3^ *J*_HH_=7.4 Hz, 8H, H^*o*^ in C_6_H_5_ ring (dppe)), 6.04 (s, 2H, C_6_ ***H***_***2***_(CH_3_)_2_), 2.75 (m, 4H, CH_2_), 2.11 (m, 4H, CH_2_), 1.88 (s, 6H, CH_3_), 1.57 (s, 30H, C_5_(C ***H***_***3***_)_5_); ^13^C{^1^H} NMR (101 MHz, CDCl_3_ **)** δ/ppm: 139.38 (*m*, C^*i*^ in C_6_H_5_ ring (dppe)), 137.34 (*m*, C^*i*^ in C_6_H_5_ ring (dppe)), 134.04 (*m*, C^*o*^ in C_6_H_5_ ring (dppe)), 133.32 (*m*, C^*o*^ in C_6_H_5_ ring (dppe)), 131.61 ((**C^2^**‐H in C_6_H_2_(CH_3_)_2_), 128.77 (C^*p*^ in C_6_H_5_ ring (dppe)), 128.74 (C^*p*^ in C_6_H_5_ ring (dppe)), 127.44 (*m*, C^*m*^ in C_6_H_5_ ring (dppe)), 127.43 (*m*, C^*m*^ in C_6_H_5_ ring (dppe)), 92.61 (***C***_**5**_(CH_3_)_5_), 29.40 (*m*, CH_2_ (dppe)), 20.41 (C_6_H_2_(***C**H_3_*)_2_), 10.31 (C_5_(***C**H_3_*)_5_); (Some of the peaks cannot be seen due to the low solubility of the compound); ^31^P{^1^H} NMR (162 MHz, CDCl_3_) δ/ppm: 81.40 (br, dppe) (The ^31^P peak broad in CDCl_3_ probably due to the presence of different conformers. The ^31^P peak of this compound was sharp in toluene*‐d_8_*); ESI‐MS *m/z*: found: 1423.3884, calc. for [M+H]^+^: 1423.3845; Anal. Calcd (C_84_H_86_P_4_Ru_2_): C, 70.97; H, 6.10 %; Found: C, 70.86; H, 6.02.

[{Ru(dppe)Cp*}_2_(μ‐C≡C*‐1,4‐C_6_H_2_{‐2,5‐(CF_3_)_2_}‐*C≡C*)]* **(4** **a)**: A mixture of RuCl(dppe)Cp* (132 mg, 197 *μ*mol), 1,4‐bis(trimethylsilylethynyl)‐2,5‐di(trifluoromethyl)benzene (40 mg, 98 *μ*mol) and potassium fluoride (15 mg, 258 *μ*mol) in methanol (12 mL) was heated at 75 °C overnight and then allowed to cool to room temperature. The color of the solution changed from orange to yellow over time with a yellow precipitate. The precipitate was collected by filtration and washed with methanol, hexanes and diethyl ether to afford **4** **a** as a yellow powder (104 mg, 68 %). Crystals suitable for X‐ray analyses could be obtained by slow diffusion of methanol into a chloroform solution of the complex. FTIR (CH_2_Cl_2_) *ν*/cm^−1^: 2056, 2048 (C≡C); FTIR (ATR) *ν*/cm^−1^: 2055 (C≡C); ^1^H NMR (600 MHz, CDCl_3_) δ/ppm: 7.69 (*t*, ^3^ *J*_HH_=8.2 Hz, 8H, H^*o*^ in C_6_H_5_ ring (dppe)), 7.33–7.24 (*m*, 24H, H^m/p^ in C_6_H_5_ ring (dppe) (partly obscured with the solvent peak), 7.15 (*t*, ^3^ *J*_HH_=7.7 Hz, 8H, H^*o*^ in C_6_H_5_ ring (dppe)), 6.39 (*s*, 2H, C_6_ ***H***_***2***_(CF_3_)_2_), 2.70 (*m*, 4H, CH_2_), 2.17 (*m*, 4H, CH_2_), 1.56 (*s*, 30 H, C_5_(C ***H***_***3***_)_5_); ^13^C{^1^H} NMR (151 MHz, CDCl_3_ **)** δ/ppm: 139.00 (*m*, C^*i*^ in C_6_H_5_ ring (dppe)), 136.74 (*m*, C^*i*^ in C_6_H_5_ ring (dppe)), 133.71 (*m*, C^*o*^ in C_6_H_5_ ring (dppe)), 133.26 (*m*, C^*o*^ in C_6_H_5_ ring (dppe)), 131.19 (***C***‐H in C_6_H_2_(CF_3_)_2_), 129.04 (C^*p*^ in C_6_H_5_ ring (dppe)), 128.93 (C^*p*^ in C_6_H_5_ ring (dppe)), 127.57 (*m*, C^*m*^ in C_6_H_5_ ring (dppe)), 127.54 (*m*, C^*m*^ in C_6_H_5_ ring (dppe)), 108.13 (C^β^), 93.13 (***C***_**5**_(CH_3_)_5_), 29.52 (*m*, CH_2_), 10.12 (C_5_(***C**H_3_*)_5_) (Some of the peaks cannot be seen in the solution ^13^C{^1^H} NMR spectrum due to low solubility); ^31^P{^1^H} NMR (243 MHz, CDCl_3_) δ/ppm: 81.60 (s, dppe); ^19^F{^1^H} NMR (565 MHz, CDCl_3_) δ/ppm: −60.77; ESI‐MS *m/z*: found: 1531.4349, calc. for [M+H]^+^: 1531.3280; Anal. Calcd (C_84_H_80_F_6_P_4_Ru_2_): C, 65.96; H, 5.27 %, Found: C, 65.87; H, 5.38.

[{Ru(dppe)Cp*}_2_(μ‐C≡C*‐1,4‐C_6_H_2_(‐2,5‐{CH(CH_3_)_2_)_2_}‐*C≡C*)]* **(5** **a)**: A mixture of RuCl(dppe)Cp* (127.3 mg, 190 *μ*mol) and 1,4‐diethynyl‐2,5‐diisopropylbenzene (20 mg, 95 *μ*mol) in methanol (10 mL) was heated at 75 °C. A clear deep red solution was obtained over time. After 1 h, 2 drops of DBU was added. The precipitated yellow powder was collected by filtration and washed with methanol, hexanes and diethyl ether (72 mg, 51 %). FTIR (CH_2_Cl_2_) *ν*/cm^−1^: 2063, 2055 (C≡C); FTIR (ATR) *ν*/cm^−1^: 2053 (C≡C); ESI‐MS *m/z*: found: 1479.4512, calc. for [M+H]^+^: 1479.4471; **Anal. Calcd** (C_88_H_92_P_4_Ru_2_): C, 71.62; H, 6.28 %, Found: C, 71.59; H, 6.30; the extremely poor solubility of this compound prevented the acquisition of satisfactory solution NMR spectra and the growth of single crystals for X‐ray measurements.

Deposition Numbers 2141736 (for **1** **a**), 2141739 (for **3** **a**), 2141740 (for **4** **a**), 2141737 (for **1** **b**), 2141738 (for **2** **b**), 2141741 (for **4** **b**), 2141742 (for **5** **b**) contain the supplementary crystallographic data for this paper. These data are provided free of charge by the joint Cambridge Crystallographic Data Centre and Fachinformationszentrum Karlsruhe Access Structures service.

## Supporting Information

Detailed descriptions of the syntheses of complexes **1** **b**–**5** **b**, Tabulated relative energies and key structural features of conformers of [**1**–**5**]^+.^ Crystal structure and refinement details, and ORTEP Tabulated TD DFT results. Tabulated orbital compositions. Tabulated calculated and experimental vibrational frequencies. Plots of UV‐vis‐NIR and IR spectroelectrochemical data

## Conflict of interest

The authors declare no competing financial interests

1

## Supporting information

As a service to our authors and readers, this journal provides supporting information supplied by the authors. Such materials are peer reviewed and may be re‐organized for online delivery, but are not copy‐edited or typeset. Technical support issues arising from supporting information (other than missing files) should be addressed to the authors.

Supporting Information

## Data Availability

The data that support the findings of this study are available in the supplementary material of this article.
