# Supplementary material for: The Use of Bridging Ligand Substituents to Bias the Population of Localized and Delocalized Mixed‐Valence Conformers in Solution
Source: Chemistry. 2022 Jun 23;28(44):e202200926. doi: 10.1002/chem.202200926 (PMC9401031; doi:10.1002/chem.202200926)
Supplement: Supplementary file 1 — Supporting Information [file CHEM-28-0-s001.pdf]

Chemistry–A European Journal

Supporting Information

The Use of Bridging Ligand Substituents to Bias the Population of Localized and Delocalized Mixed-Valence Conformers in Solution

Parvin Safari, Simon Gückel, Josef B. G. Gluyas, Stephen A. Moggach, Martin Kaupp,* and Paul J. Low*

General Reaction Conditions 3
[{Ru(dppe)Cp}$_2$(μ-C≡C-1,4-C_6H_4-C≡C)] (**1b**) 3
[{Ru(dppe)Cp}$_2$(μ-C≡C-1,4-C_6F_4-C≡C)] (**2b**) 3
[{Ru(dppe)Cp}$_2$(μ-C≡C-1,4-C_6H_2(-2,5-CH_3)$_2$}-C≡C)] (**3b**) 4
[{Ru(dppe)Cp}$_2$(μ-C≡C-1,4-C_6H_2-{2,5-(CF_3)$_2$}-C≡C)] (**4b**) 4
[{Ru(dppe)Cp}$_2$(μ-C≡C-1,4-C_6H_2-{2,5-CH(CH_3)$_2$)$_2$}-C≡C)] (**5b**) 5
[{Ru(dppe)Cp*}$_2$(μ-C≡C-1,4-C_6H_4-C≡C)]PF_6 ([**1a**]PF_6) 6
Crystallographic structure determination 7
Table S1. Summary of the relative energies and key structural features of the conformers of [**1 – 5**]$^+$. 8
Table S2. Crystal structure and refinement details 11
Figure S1. ORTEP illustrations of molecules of: (a) **1a**; (b) **3a**; (c) **4a**; (d) **1b**; (e) **2b**; (f) **4b**; (g) **5b**. 13
Table S3. TDDFT results (BLYP35-D3/de2-SVP/COSMO(CH_2Cl_2)) for compounds [**1 – 5**]$^+$ summarizing the energy (E / cm^{-1}) and majority composition of transitions. 14
Table S4. Orbital composition (β-spin) for conformers of [**1 – 5**]$^+$. 20
Table S5. Calculated vibrational frequencies for conformers of [**1 – 5**]$^+$ † 24
Table S6. Summary of IR data from [**1a,b – 5a,b**]$^+$ 27
Figure S2. Plots of the UV-vis-NIR spectra of [**1b – 5b**]$^+$ from spectroelectrochemical data. 28
Figure S3. Plots of the spectroelectrochemically generated IR spectra of [**1b – 5b**]$^+$. 29
Figure S4: Plots of spin density distributions of each conformer of [**1**]$^+$ 30
Figure S5: Plots of spin density distributions of each conformer of [**2**]$^+$ 31
Figure S6: Plots of spin density distributions of each conformer of [**3**]$^+$ 32
Figure S7: Plots of spin density distributions of each conformer of [**4**]$^+$ 33
Figure S8: Plots of spin density distributions of each conformer of [**5**]$^+$ 34
Figure S9: Plots and energies (eV) of frontier orbitals of each conformer of [**1**]$^+$ 37
Figure S10: Plots and energies (eV) of frontier orbitals of each conformer of [**2**]$^+$ 40
Figure S11: Plots and energies (eV) of frontier orbitals of each conformer of [**3**]$^+$ 44
Figure S12: Plots and energies (eV) of frontier orbitals of each conformer of [**4**]$^+$ 47
Figure S13: Plots and energies (eV) of frontier orbitals of each conformer of [**5**]$^+$ 50
Figure S14. The IR spectra of [**1a**]PF_6: (a) in CH_2Cl_2 solution; (b) from solid samples precipitated from CH_2Cl_2 solution at room temperature (red) or -78°C (blue) by addition of Et_2O and recorded as a Nujol mull. 51
Figure S15. The ^1H NMR spectrum of 1,4-bis((trimethylsilyl)ethynyl)-2,5-dimethylbenzene 52
Figure S17. The ^1H NMR spectrum of [{Ru(dppe)Cp*}$_2$(μ-C≡C-1,4-C_6H_2{2,5-(CH_3)$_2$}-C≡C)] (**3a**) 54
Figure S18. The $^{31}P\{^1H\}$ NMR spectrum of [{Ru(dppe)Cp*}$_2$(μ-C≡C-1,4-C_6H_2{2,5-(CH_3)$_2$}-C≡C)] (**3a**) in $CDCl_3$. 55

Figure S19. The $^{31}P\{^{1}H\}$ NMR spectrum of [{Ru(dppe)Cp*}$_2$(μ-C≡C-1,4-C_6H_2\{2,5-$(CH_3)_2$\}-C≡C)] (**3a**) in toluene-d_8. 56
Figure S20. The $^{13}C\{^{1}H\}$ NMR spectrum of [{Ru(dppe)Cp*}$_2$(μ-C≡C-1,4-C_6H_2\{2,5-$(CH_3)_2$\}-C≡C)] (**3a**). 57
Figure S21. The ^{1}H NMR spectrum of [{Ru(dppe)Cp*}$_2$(μ-C≡C-1,4-C_6H_2\{2,5-$(CF_3)_2$\}-C≡C)] (**4a**). 58
Figure S22. The $^{31}P\{^{1}H\}$ NMR spectrum of [{Ru(dppe)Cp*}$_2$(μ-C≡C-1,4-C_6H_2\{2,5-$(CF_3)_2$\}-C≡C)] (**4a**). 59
Figure S23. The $^{19}F\{^{1}H\}$ NMR spectrum of [{Ru(dppe)Cp*}$_2$(μ-C≡C-1,4-C_6H_2\{2,5-$(CF_3)_2$\}-C≡C)] (**4a**). 60
Figure S24. The $^{13}C\{^{1}H\}$ NMR spectrum of [{Ru(dppe)Cp*}$_2$(μ-C≡C-1,4-C_6H_2\{2,5-$(CF_3)_2$\}-C≡C)] (**4a**). 61
Figure S25. The ^{1}H NMR spectrum of [{Ru(dppe)Cp}$_2$(μ-C≡C-1,4-C_6H_2\{2,5-$(CH_3)_2$\}-C≡C)] (**3b**). 62
Figure S26. The $^{31}P\{^{1}H\}$ NMR spectrum of [{Ru(dppe)Cp}$_2$(μ-C≡C-1,4-C_6H_2\{2,5-$(CH_3)_2$\}-C≡C)] (**3b**). 63
Figure S27. The $^{13}C\{^{1}H\}$ NMR spectrum of [{Ru(dppe)Cp}$_2$(μ-C≡C-1,4-C_6H_2\{2,5-$(CH_3)_2$\}-C≡C)] (**3b**). 64
Figure S28. The ^{1}H NMR spectrum of [{Ru(dppe)Cp}$_2$(μ-C≡C-1,4-C_6H_2\{2,5-$(CF_3)_2$\}-C≡C)] (**4b**). 65
Figure S29. The $^{31}P\{^{1}H\}$ NMR spectrum of [{Ru(dppe)Cp}$_2$(μ-C≡C-1,4-C_6H_2\{2,5-$(CF_3)_2$\}-C≡C)] (**4b**). 66
Figure S30. The $^{19}F\{^{1}H\}$ NMR spectrum of [{Ru(dppe)Cp}$_2$(μ-C≡C-1,4-C_6H_2\{2,5-$(CF_3)_2$\}-C≡C)] (**4b**). 67
Figure S31. The $^{13}C\{^{1}H\}$ NMR spectrum of [{Ru(dppe)Cp}$_2$(μ-C≡C-1,4-C_6H_2\{2,5-$(CF_3)_2$\}-C≡C)] (**4b**). 68
Figure S32. The ^{1}H NMR spectrum of [{Ru(dppe)Cp}$_2$(μ-C≡C-1,4-C_6H_2-\{2,5-$CH(CH_3)_2)_2$\}-C≡C)] (**5b**). 69
Figure S33. The $^{31}P\{^{1}H\}$ NMR spectrum of [{Ru(dppe)Cp}$_2$(μ-C≡C-1,4-C_6H_2-\{2,5-$CH(CH_3)_2)_2$\}-C≡C)] (**5b**). 70
Figure S34. The $^{13}C\{^{1}H\}$ NMR spectrum of [{Ru(dppe)Cp}$_2$(μ-C≡C-1,4-C_6H_2-\{2,5-$CH(CH_3)_2)_2$\}-C≡C)] (**5b**). 71
References 72

General Reaction Conditions

All reactions were carried out under an atmosphere of dry nitrogen using standard Schlenk techniques and reaction solvents were sparged with nitrogen. Methanol was dried by distillation from iodine/magnesium turning. Diethylether and tetrahydrofuran were dried on an Inert™ solvent purification system. Diisopropylamine and triethylamine were pre-dried over potassium hydroxide and distilled from calcium hydride before use and stored under N_2. Other solvents were standard reagent grade and used as received. No special precautions were taken to exclude air or moisture during workup except where otherwise indicated.

The precursors RuCl(dppe)Cp,[1] 1,4-bis(trimethylsilylethynyl)-2,5-di(trifluoromethyl)benzene,[2] and complexes **1b** and **2b**[3] were prepared by literature methods. The ligand precursor 1,4-diethynyl-2,5-dimethylbenzene was prepared by a minor modification of the literature route,[4] as described below. All other reagents were purchased and used as received.

[{Ru(dppe)Cp}$_2$(μ-C≡C-1,4-C_6H_4-C≡C)] ***(1b)***

Compound **1b** was prepared by the literature method,[3] and crystals suitable for X-ray measurements were grown by slow diffusion of methanol into a solution of the complex in dichloromethane.

[{Ru(dppe)Cp}$_2$(μ-C≡C-1,4-C_6F_4-C≡C)] ***(2b)***

Compound **2b** was prepared by the literature method,[3] and crystals suitable for X-ray measurements were grown by slow diffusion of methanol into a solution of the complex into a dichloromethane.

[{Ru(dppe)Cp}$_2$(μ-C≡C-1,4-C$_6$H$_2$(-2,5-CH$_3$)$_2$}-C≡C)] ***(3b)***
A 50-ml Schlenk flask was charged with RuCl(dppe)Cp (100 mg, 167 μmol), 1,4-bis(trimethylsilylethynyl)-2,5-dimethylbenzene (25.0 mg, 84 μmol), potassium fluoride (12.0 mg, 207 μmol), methanol (8 mL) and THF (3 mL). The solution was heated at 65 °C for 16 h, resulting in the precipitation of the product as a yellow power. Finally, the product was collected by filtration, washed with methanol, hexanes and diethyl ether and dried under vacuum (65 mg, 61 %). **FTIR (CH_2Cl_2)** *ν*/cm^{-1}**:** 2077, 2066 (C≡C); **FTIR (ATR)** *ν*/cm^{-1}**:** 2059 (C≡C); **^{1}H NMR (400 MHz, CDCl$_3$)** δ/ppm: 7.88 (*t*, $^3J_{HH}$ = 8.0 Hz, 8H, H^o in C_6H_5 ring (dppe)), 7.32-7.17 (*m*, 32H, H$^{o/m/p}$ in C_6H_5 ring (dppe) (partly obscured with the solvent peak), 5.72 (*s*, 2H, C$_6$***H$_2$***(CH$_3$)$_2$), 4.75 (*s*, 10H, C_5H_5), 2.66 (*m*, 4H, CH_2), 2.30 (*m*, 4H, CH_2), 1.42 (*s*, 12 H, CH_3); **^{13}C{^{1}H} NMR (101 MHz, CDCl$_3$)** δ/ppm: 134.26 (*t*, $^2J_{CP}$ = 5.7 Hz, C^o in C_6H_5 ring (dppe)), 131.52 (*t*, $^2J_{CP}$ = 5.2 Hz, C^o in C_6H_5 ring (dppe)), 129.26 (C in C_6H_5 ring (dppe)^p), 128.66 (C^p in C_6H_5 ring (dppe)), 127.87 (*t*, $^2J_{CP}$ = 5.3 Hz, C^m in C_6H_5 ring (dppe)), 127.75 (*t*, $^2J_{CP}$ = 5.5 Hz, C^m in C_6H_5 ring (dppe)), 82.55 (*t*, $^3J_{CP}$ = 2.5 Hz, C_5H_5), 28.07 (*m*, CH_2), 19.84 (CH_3) (Some of the carbon peaks didn't appear due to the low solubility of the compound); **^{31}P{^{1}H} NMR (162 MHz, CDCl$_3$)** δ/ppm**:** 87.22 (s, dppe); **ESI-MS** *m/z:* found: 1283.2316, calc. for [M+H]$^+$: 1283.2280;

[{Ru(dppe)Cp}$_2$(μ-C≡C-1,4-C$_6$H$_2$-{2,5-(CF$_3$)$_2$}-C≡C)] ***(4b)***
A Schlenk flask was charged with methanol (12 ml) / THF (4 ml) before RuCl(dppe)Cp (118 mg, 197 μmol), 1,4-bis(trimethylsilylethynyl)-2,5-di(trifluoromethyl)benzene (40 mg, 98 μmol) and potassium fluoride (20.0 mg, 345 μmol) were added. The solution was heated at 70°C for 16h, during which time

the product precipitated as a yellow solid. Finally, the product (60 mg, 44%) was collected by filtration, and washed with methanol. Crystals suitable for X-Ray analysis were obtained by slow diffusion of hexanes into a chloroform solution of the complex. **FTIR (CH_2Cl_2)** ν/cm^{-1}**:** 2061 (C≡C); **FTIR (ATR)** ν/cm^{-1}**:** 2046 (C≡C); **^{1}H NMR (500 MHz, $CDCl_3$)** δ/ppm: 7.83 (*t*, $^{3}J_{HH}$ = 7.5 Hz, , 8H, H^{o} in C_6H_5 ring (dppe)), 7.30-7.21 (*m*, 24H, $H^{m/p}$ in C_6H_5 ring (dppe) (partly obscured with the solvent peak), 7.18 (*t*, $^{3}J_{HH}$ = 8.1 Hz, 8H, H^{o} in C_6H_5 ring (dppe)), 6.38 (s, 2H, $C_6\boldsymbol{H_2}(CF_3)_2$), 4.77 (s, 10H, C_5H_5), 2.64 (m, 4H, CH_2), 2.36 (m, 4H, CH_2); **^{13}C{^{1}H} NMR (126 MHz, $CDCl_3$)** δ/ppm: 142.68 (*m*, C^{i} in C_6H_5 ring (dppe)), 136.70 (*m*, C^{i} in C_6H_5 ring (dppe)), 133.92 (*t*, $^{2}J_{CP}$ = 5.2 Hz, C^{o} in C_6H_5 ring (dppe)), 131.51 (*t*, $^{2}J_{CP}$ = 5.2 Hz, C^{o} in C_6H_5 ring (dppe)), 130.72 (br, ***C***-H in $C_6H_2(CF_3)_2$), 129.87 (q, $^{2}J_{CF}$ = 29 Hz, $\boldsymbol{C^{3}}$-CF_3 in $C_6H_2(CF_3)_2$), 129.48 (C^{p} in C_6H_5 ring (dppe)), 128.83 (C^{p} in C_6H_5 ring (dppe)), 127.96 (*t*, $^{2}J_{CP}$ = 4.3 Hz, C^{m} in C_6H_5 ring (dppe)), 127.78 (*t*, $^{2}J_{CP}$ = 4.8 Hz, C^{m} in C_6H_5 ring (dppe)), 127.60 (*t*, $^{2}J_{CP}$ =25.1 Hz, C^{α}), 123.84 (q, $^{1}J_{CF}$ = 273.8 Hz, **C**F_3), 121.21 (C^{i} in $C_6H_2(CF_3)_2$), 108.96 (C^{β}), 83.09 (*t*, $^{3}J_{CP}$ = 2.5 Hz, C_5H_5), 28.29 (m, CH_2); **^{31}P{^{1}H} NMR (202 MHz, $CDCl_3$)** δ/ppm: 86.40 (s, dppe); **^{19}F{^{1}H} NMR (471 MHz, $CDCl_3$)** δ/ppm: -61.28; **ESI-MS** *m/z:* found: 1391.1750, calc. for $[M+H]^{+}$: 1391.1715;

[{Ru(dppe)Cp}$_2$(μ-C≡C-1,4-C_6H_2-{2,5-$CH(CH_3)_2$}$_2$}-C≡C)] ***(5b)***

To a 50-ml Schlenk flask containing methanol (8 mL) and tetrahydrofuran (3 mL), RuCl(dppe)Cp (100 mg, 167 μmol), 1,4-bis((trimethylsilyl)ethynyl)-2,5-diisopropylbenzene (30 mg, 85 μmol) and potassium fluoride (12 mg, 207 μmol) were added. The solution was heated at 65 °C for 16 h and the product, which precipitated over time as a yellow solid (54 mg, 49%), was collected by filtration, washed with

methanol, hexanes and diethyl ether and dried under vacuum. Crystals suitable for X-ray measurements were obtained by slow diffusion of methanol into a dichloromethane solution of the compound. **FTIR (CH_2Cl_2)** ν/cm^{-1}**:** 2071, 2062 (C≡C); **FTIR (ATR)** ν/cm^{-1}**:** 2060 (C≡C); **^{1}H NMR (400 MHz, $CDCl_3$)** δ/ppm**:** 7.90 (*m*, 8H, H^o in C_6H_5 ring (dppe)), 7.34 (m, 12H, $H^{m/p}$ in C_6H_5 ring (dppe)), 7.22-7.15 (m, 12H, $H^{m/p}$ in C_6H_5 ring (dppe)), 7.11 (*t*, $^3J_{HH}$ = 8.4 Hz, 8H, H^o in C_6H_5 ring (dppe)), 6.07 (s, 2H, $C_6\boldsymbol{H_2}(CH(CH_3)_2)_2$), 4.75 (s, 10H, C_5H_5), 2.68 (m, 4H, CH_2), 2.47 (*septet*, $^3J_{HH}$ = 6.8 Hz, 2H, $C\boldsymbol{H}(CH_3)_2$), 2.38 (m, 4H, CH_2), 0.58 (*d*, $^3J_{HH}$ = 6.8 Hz, 12H, $CH(C\boldsymbol{H_3})_2$); **^{31}P{^{1}H} NMR (162 MHz, $CDCl_3$)** δ/ppm: 87.85 (s, dppe); **^{13}C{^{1}H} NMR (101 MHz, $CDCl_3$)** δ/ppm: 134.39 (*t*, $^2J_{CP}$ = 5.5 Hz, C^o in C_6H_5 ring (dppe)), 131.36 (*t*, $^2J_{CP}$ = 5.1 Hz, C^o in C_6H_5 ring (dppe)), 129.45 (C^p in C_6H_5 ring (dppe)), 128.53 (C^p in C_6H_5 ring (dppe)), 127.84 (*t*, $^2J_{CP}$ = 4.8 Hz, C^m), 127.78 (*t*, $^2J_{CP}$ = 4.8 Hz, C^m), 109.98, 82.66 (*t*, $^3J_{CP}$ = 1.7 Hz, C_5H_5), 28.11 (*m*, CH_2), 23.41 (*s*, $CH(\boldsymbol{C}H_3)_2$) (Some of the carbon peaks didn't appear due to the low solubility of the compound); **ESI-MS** *m/z:* found: 1339.2943, calc. for $[M+H]^+$: 1339.2906.

[{Ru(dppe)Cp}$_2$(μ-C≡C-1,4-C_6H_4-C≡C)]PF_6* ***([1a]PF$_6$)***

A solution of **1a** (50 mg, 35.8 mmol) in CH_2Cl_2 (10 ml) was treated with $[FeCp_2]PF_6$ causing an immediate change in the colour of the solution to deep red. The reaction was allowed to stir for 20 minutes and monitored by IR spectroscopy. When the reaction was adjudged complete, the solution was concentrated to 1 ml. To prepare the room temperature sample, the concentrated CH_2Cl_2 solution of the crude reaction mixture was added dropwise to hexanes (20 ml) whilst being rapidly stirred. The low temperature sample was prepared by cooling the CH_2Cl_2 solution to -78°C in a dry-ice / acetone bath. Hexanes (20 ml) were cooled to -78°C before the cold CH_2Cl_2 solution

was added dropwise with rapid stirring. In both cases, the resulting deep red precipitate was collected by filtration, washed with diethynyl ether and dried under vaccum to give [**1a**]PF_6 in essentially quantitative yield.

Crystallographic structure determination

Data were collected using a XtaLAB Synergy, Single source at home/near, HyPix diffractometer. Data were measured using Cu Kα radiation. The diffraction pattern was indexed and the total number of runs and images was based on the strategy calculation from the program CrysAlisPro 1.171.41.103a.[5] Data reduction, scaling and absorption corrections were performed using CrysAlisPro.

The crystals were kept at a steady T = 100 K during data collection. The structures were solved with the ShelXT 2018/2 solution program using dual methods,[6] and by using Olex2 1.3 as the graphical interface.[7] The models were refined with XL using full matrix least squares minimisation on F^2.[8] All crystallographic data have been deposited with the CCDC (2141736-2141742) and can be obtained free of charge via https://www.ccdc.cam.ac.uk/structures/, or from the Cambridge Crystallographic Data Centre, 12 Union Road, Cambridge CB2 1EZ, UK (fax +441223336033; email deposit@ccdc.cam.ac.uk). Crystallographic data have been tabulated and given below (Table S2).

Table S1. Summary of the relative energies and key structural features of the conformers of [**1** – **5**]$^{+}$.

	Ω	θ_1	θ_2	α	rel E	Ru1-P11	Ru2-P12	Ru1-C1	C1-C2	(C9-C10)	(C10-Ru2)	Ru2-P21	Ru2-P22
	°	°	°	°	kJ/mol	Å	Å	Å	Å	Å	Å	Å	Å
[**1**]$^{+}$	2.8	-1.3	3.9	165.3	0.0	2.3205	2.3261	1.9392	1.2429	1.3941	1.3932	1.2429	1.9394
	29.0	2.8	-31.9	163.6	1.8	2.3211	2.3296	1.9338	1.2440	1.3929	1.3962	1.2417	1.9477
	38.2	13.6	21.8	151.3	0.5	2.3143	2.3397	1.9386	1.2447	1.3952	1.3936	1.2421	1.9418
	92.3	-0.5	-87.3	178.5	13.6	2.3360	2.3437	1.9250	1.2446	1.3986	1.4071	1.2344	1.9792
	143.0	3.3	23.6	162.9	6.1	2.3171	2.3303	1.9365	1.2436	1.3930	1.3948	1.2422	1.9444
	153.8	7.7	-43.9	160.2	6.6	2.3191	2.3484	1.9293	1.2457	1.3951	1.3984	1.2384	1.9597
	155.1	9.8	-44.6	163.5	6.6	2.3208	2.3476	1.9290	1.2456	1.3952	1.3988	1.2379	1.9609
	164.3	-11.1	-12.7	166.7	3.9	2.3176	2.3288	1.9375	1.2434	1.3932	1.3947	1.2421	1.9422
	168.5	1.8	1.9	165.4	4.8	2.3157	2.3273	1.9400	1.2429	1.3933	1.3933	1.2429	1.9400
	179.3	0.6	-0.6	178.9	8.1	2.3175	2.3256	1.9404	1.2429	1.3932	1.3932	1.2428	1.9405
[**2**]$^{+}$	25.5	11.9	-52.4	154.0	5.4	2.3358	2.3610	1.9210	1.2436	1.3920	1.3932	1.2383	1.9527
	36.0	17.4	15.5	151.8	0.0	2.3353	2.3390	1.9182	1.2434	1.3860	1.3892	1.2429	1.9332
	138.2	-18.7	-38.9	148.9	1.1	2.3306	2.3698	1.9261	1.2399	1.3965	1.3969	1.2366	1.9563
	169.7	-5.8	-12.8	165.8	5.5	2.3143	2.3444	1.9204	1.2428	1.3842	1.3836	1.2418	1.9245

	170.8	3.5	-0.4	165.8	8.3	2.3225	2.3336	1.9201	1.2426	1.3831	1.3832	1.2423	1.9219
	172.1	1.1	1.0	165.7	8.1	2.3219	2.3332	1.9205	1.2425	1.3831	1.3832	1.2424	1.9215
	172.2	-3.4	-17.9	157.2	4.3	2.3146	2.3463	1.9195	1.2431	1.3848	1.3846	1.2422	1.9272
[**3**]$^{+}$	-173.9	29.6	-28.8	168.4	9.7	2.3222	2.3284	1.9313	1.2431	1.3925	1.3958	1.2408	1.9498
	-159.5	-20.0	30.6	166.9	9.8	2.3127	2.3295	1.9425	1.2429	1.3927	1.3941	1.2425	1.9434
	-158.6	6.4	9.1	164.9	3.9	2.3132	2.3277	1.9409	1.2428	1.3927	1.3929	1.2429	1.9413
	-115.1	9.9	38.8	156.5	9.0	2.3138	2.3416	1.9364	1.2449	1.3935	1.3953	1.2406	1.9493
	-96.5	38.0	37.9	171.7	10.4	2.3165	2.3291	1.9420	1.2425	1.3939	1.3939	1.2424	1.9423
	-32.8	13.6	-44.6	165.3	4.1	2.3216	2.3299	1.9322	1.2447	1.3923	1.3967	1.2398	1.9535
	41.7	13.0	24.6	152.3	0.0	2.3132	2.3398	1.9374	1.2447	1.3939	1.3943	1.2417	1.9475
	62.6	17.7	41.7	175.4	7.2	2.3213	2.3286	1.9338	1.2445	1.3926	1.3968	1.2402	1.9531
	119.4	12.9	-81.2	153.1	13.9	2.3255	2.3466	1.9242	1.2461	1.3953	1.4081	1.2366	1.9835
	125.9	-24.0	-41.2	148.3	14.9	2.3099	2.3459	1.9264	1.2446	1.3942	1.4034	1.2408	1.9640
	146.2	-24.5	-17.7	163.9	2.1	2.3181	2.3195	1.9442	1.2424	1.3934	1.3923	1.2424	1.9387
	155.2	12.0	-47.8	157.9	8.3	2.3173	2.3448	1.9307	1.2458	1.3934	1.3975	1.2386	1.9582
	160.2	2.1	-28.7	166.6	5.7	2.3218	2.3365	1.9310	1.2446	1.3937	1.3978	1.2396	1.9559
[**4**]$^{+}$	-169.7	1.6	1.8	165.7	6.2	2.3214	2.3340	1.9259	1.2439	1.3890	1.3890	1.2437	1.9273
	-119.2	10.8	34.5	157.6	0.0	2.3248	2.3536	1.9232	1.2446	1.3933	1.3909	1.2398	1.9404
	-64.0	-17.3	-47.8	147.3	5.9	2.3217	2.3556	1.9235	1.2423	1.3990	1.3944	1.2381	1.9489

	-35.6	1.0	-35.0	162.9	1.2	2.3288	2.3402	1.9206	1.2443	1.3913	1.3893	1.2407	1.9375
	27.1	-3.5	29.4	162.8	1.9	2.3268	2.3398	1.9203	1.2440	1.3892	1.3893	1.2419	1.9328
	33.2	4.8	32.5	150.2	1.1	2.3245	2.3527	1.9220	1.2457	1.3932	1.3893	1.2397	1.9370
	99.2	-34.1	-34.3	159.6	4.2	2.3297	2.3418	1.9256	1.2428	1.3880	1.3880	1.2428	1.9260
	122.7	-22.6	-42.0	149.2	7.1	2.3279	2.3644	1.9270	1.2403	1.2390	1.9648	2.3015	2.3186
	147.7	-4.0	-37.3	162.9	10.7	2.3323	2.3511	1.9221	1.2434	1.2391	1.9534	2.3148	2.3201
	164.6	-5.4	-19.3	166.1	9.1	2.3218	2.3549	1.9225	1.2439	1.2418	1.9450	2.3115	2.3260
[**5**]$^{+}$	-165.4	5.4	5.5	166.2	0.2	2.3126	2.3334	1.9387	1.2420	1.3925	1.3925	1.2420	1.9387
	-148.9	11.5	26.0	169.3	0.0	2.3202	2.3347	1.9329	1.2432	1.3919	1.3969	1.2411	1.9514
	-132.6	15.0	41.1	159.8	1.8	2.3337	2.3347	1.9279	1.2447	1.3930	1.4002	1.2397	1.9599
	-68.9	-17.2	-46.9	175.7	6.0	2.3228	2.3442	1.9291	1.2442	1.3920	1.3974	1.2386	1.9550
	-62.0	13.4	-76.8	176.8	16.5	2.3301	2.3464	1.9221	1.2450	1.3951	1.4096	1.2365	1.9827
	-14.9	15.7	-27.3	164.0	6.0	2.3192	2.3338	1.9349	1.2428	1.3920	1.3961	1.2412	1.9484
	28.4	-15.0	41.5	168.8	6.1	2.3238	2.3375	1.9323	1.2441	1.3921	1.3956	1.2394	1.9507
	34.7	19.1	13.6	167.4	1.9	2.3144	2.3284	1.9408	1.2424	1.3933	1.3933	1.2419	1.9401
	104.6	11.2	-89.9	174.3	12.4	2.3347	2.3366	1.9231	1.2448	1.3971	1.4107	1.2357	1.9851
	165.5	10.7	-41.2	161.8	11.1	2.3205	2.3379	1.9350	1.2447	1.3946	1.3970	1.2382	1.9546

Table S2. Crystal structure and refinement details

	**1a**	**3a**	**4a**	**1b**	**2b**	**4b**	**5b**
CCDC Number	2141736	2141739	2141740	2141737	2141738	2141741	2141742
Empirical formula	$C_{82}H_{82}P_4Ru_2$	$C_{84}H_{86}P_4Ru_2$	$C_{84}H_{80}F_6P_4Ru_2$	$C_{73.5}Cl_3H_{65}P_4Ru_2$	$C_{73}Cl_2F_4H_{60}P_4Ru_2$	$C_{77}H_{63}Cl_9F_6P_4Ru_2$	$C_{80}Cl_4H_{78}P_4Ru_2$
Formula weight	1393.49	1421.54	1529.5	1380.62	1410.13	1747.34	1507.24
Temperature/K	99.9(6)	150.01(10)	150.00(10)	99.9(5)	100(1)	100.0(3)	100.0(3)
Crystal system	orthorhombic	monoclinic	monoclinic	monoclinic	monoclinic	monoclinic	triclinic
Space group	*Pbca*	$P2_1/n$	$P2_1/n$	*I*2/*a*	*I*2/*a*	$P2_1/c$	*P*-1
a/Å	17.2210(4)	12.4638(3)	12.2181(2)	17.1392(3)	17.16840(10)	9.46100(10)	10.6508(3)
b/Å	19.3518(5)	22.0351(9)	22.5799(4)	15.8481(3)	15.99560(10)	15.0192(2)	12.1467(4)
c/Å	20.3313(5)	13.0981(3)	13.0733(2)	22.4607(4)	22.86910(10)	26.2213(2)	15.1697(3)
α/°	90	90	90	90	90	90	77.198(2)
β/°	90	98.017(2)	98.267(2)	104.322(2)	103.9260(10)	97.7490(10)	75.264(2)
γ/°	90	90	90	90	90	90	68.159(3)
V/Å^3	6775.6(3)	3562.12(19)	3569.23(10)	5911.25(19)	6095.69(6)	3691.93(7)	1744.04(9)
Z	4	2	2	4	4	2	1
ϱ_{calc} g/cm^3	1.366	1.325	1.423	1.551	1.537	1.572	1.435
μ/mm^{-1}	4.838	4.611	4.769	6.759	6.273	7.618	6.119
F(000)	2888	1476	1572	2820	2864	1760	774
Crystal size/mm^3	0.166×0.063×0.056	0.071×0.058×0.035	0.098×0.074×0.063	0.156×0.067×0.063	0.278×0.209×0.135	0.22×0.067×0.055	0.08×0.07×0.063
Radiation	CuKα (λ = 1.54178)	CuKα (λ = 1.54184)	CuKα (λ = 1.54184)	CuKα (λ = 1.54184)	CuKα (λ = 1.54184)	CuKα (λ = 1.54184)	CuKα (λ = 1.54184)
2Θ range for data collection/°	8.132 to 152.336	7.91 to 155.03	7.83 to 155.246	6.9 to 152.284	6.812 to 151.208	6.798 to 151.3	6.086 to 151.224
Index ranges	$-21 \le h \le 21$, $-23 \le k \le 24$, $-22 \le l \le 25$	$-15 \le h \le 14$, $-25 \le k \le 24$, $-16 \le l \le 16$	$-15 \le h \le 15$, $-28 \le k \le 27$, $-16 \le l \le 15$	$-21 \le h \le 21$, $-19 \le k \le 19$, $-28 \le l \le 27$	$-21 \le h \le 21$, $-19 \le k \le 19$, $-28 \le l \le 28$	$-10 \le h \le 11$, $-18 \le k \le 18$, $-32 \le l \le 32$	$-13 \le h \le 13$, $-15 \le k \le 14$, $-18 \le l \le 18$
Reflections collected	113835	72118	62271	54311	57624	108863	63630
Independent reflections	6781 [R_{int} = 0.1441, R_{sigma} = 0.0574]	7119 [R_{int} = 0.1105, R_{sigma} = 0.0496]	7308 [R_{int} = 0.1593, R_{sigma} = 0.0582]	6042 [R_{int} = 0.1363, R_{sigma} = 0.0530]	6260 [R_{int} = 0.0625, R_{sigma} = 0.0258]	7568 [R_{int} = 0.1328, R_{sigma} = 0.0389]	6949 [R_{int} = 0.0745, R_{sigma} = 0.0322]

Data/restraints /parameters	6781/0/378	7119/0/412	7308/0/438	6042/0/326	6260/0/350	7568/18/431	6949/0/381
Goodness-of-fit on F^2	1.127	1.176	1.184	1.068	1.076	1.038	1.046
Final R indexes [$I \geq 2\sigma(I)$]	R_1 = 0.0728, wR_2 = 0.1686	R_1 = 0.0553, wR_2 = 0.1481	R_1 = 0.0653, wR_2 = 0.1966	R_1 = 0.0677, wR_2 = 0.1840	R_1 = 0.0315, wR_2 = 0.0817	R_1 = 0.0692, wR_2 = 0.1701	R_1 = 0.0452, wR_2 = 0.1174
Final R indexes [all data]	R_1 = 0.0885, wR_2 = 0.1829	R_1 = 0.0749, wR_2 = 0.1869	R_1 = 0.0740, wR_2 = 0.2288	R_1 = 0.0719, wR_2 = 0.1883	R_1 = 0.0324, wR_2 = 0.0824	R_1 = 0.0753, wR_2 = 0.1755	R_1 = 0.0486, wR_2 = 0.1196
Largest diff. peak/hole / e Å^{-3}	1.24/-1.41	1.07/-1.88	1.01/-2.18	2.72/-0.97	0.61/-0.85	2.15/-1.68	2.31/-1.25

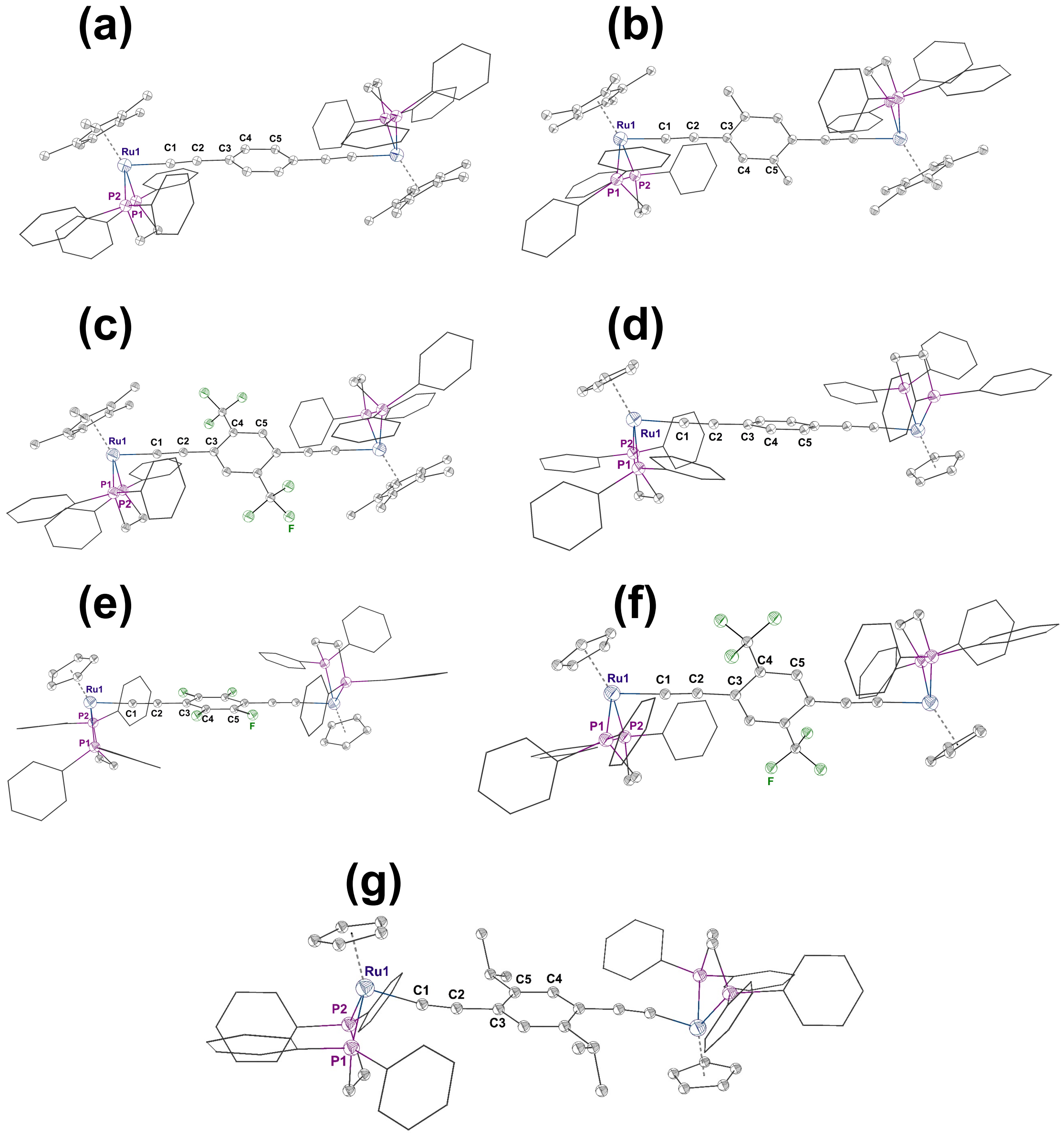

Figure S1. ORTEP illustrations of molecules of: (a) **1a**; (b) **3a**; (c) **4a**; (d) **1b**; (e) **2b**; (f) **4b**; (g) **5b**.

Table S3. TDDFT results (BLYP35-D3/de2-SVP/COSMO(CH_2Cl_2)) for compounds [**1** – **5**]$^+$ summarizing the energy (E / cm^{-1}) and majority composition of transitions.

	Ω		E/cm^{-1}	$H^a \rightarrow S^b$	H − 1 → S	H − 2 → S	H − 3 → S	H − 4 → S	H − 5 → S
[**1**]$^+$	2.8	E1	**6406**	96.1					
		E2	**9145**		73.9	8.1	9.5		
		E3	9478		8.9	70.8		9	
		E4	13630		8.1		66.9	13.1	
		E5	13742			10.5	11.7	67	
		E6	17536						82.7
	29.0	E1	**6603**	95.7					
		E2	8735			75		16.1	
		E3	**9842**		76.9		13.2		
		E4	13099			14.4		73.6	
		E5	**14060**		13.7		77.4		
		E6	17925						79.6
	38.2	E1	**6319**	95.4					
		E2	**9081**		37	42.2		8.6	
		E3	**9473**		42.1	36.1		8.2	
		E4	13393			12.2	16.6	61.3	
		E5	**13614**		11.6		61.3	15.7	
		E6	**17728**						80.4
	92.3	E1	6828				76.7	17.3	
		E2	**8973**	81.9	8.2				
		E3	**9249**	11.7	72.3	9			
		E4	10919				12	69.8	
		E5	**14208**		15	78.8			
		E6	19436						4.5
	143.0	E1	**6607**	95.9					
		E2	**9024**		5	72.9		16	
		E3	**9775**		74.5	4.8	13.9		
		E4	13346			15.1		74.3	
		E5	14002		14.3		77.2		
		E6	17758						81.6
	153.8	E1	**6767**	94.8					
		E2	7814				73.4	17.6	
		E3	**9961**		79.4	12.3			
		E4	12015				14.8	72.7	
		E5	**13991**		13.8	79.2			
		E6	18337						66
	155.1	E1	**6859**	94.4					
		E2	**7787**				75	18.3	
		E3	**10019**		80.2	13.2			
		E4	11928				14.8	72.8	
		E5	**14044**		13.9	80			
		E6	18375						65.3
	164.3	E1	**6189**	95					
		E2	8769		26	54.2	8.5		
		E3	**9381**		56.2	24.2		10.1	
		E4	13113			11.2	45.6	30	
		E5	**13655**		9		32.3	48.2	
		E6	17576				4.3		76.5
	168.5	E1	**6641**	96.5					
		E2	9318		79.2		15.7		
		E3	9664			78.4		16.4	
		E4	13733		14.6		76.8		

		E5	13859			15		77.2	
		E6	17755						83.4
	179.3	E1	**6636**	96.5					
		E2	9320		78.5		16.1		
		E3	9645			77.6		16.5	
		E4	13713		14.6		77.3		
		E5	13817			15.1		76.3	
		E6	17740						83.8
[**2**]$^+$	25.5	E1	**6607**	8.6			65.8		16
		E2	**6973**	85.4			6.5		
		E3	10047				13.3	5.8	60.9
		E4	**10347**		83.4	9.6			
		E5	**13929**		10.6	82.4			
		E6	**18605**					31.8	
	36.0	E1	**6001**	88.8		4.1			
		E2	**7656**	6.5		71.2		12.7	
		E3	10192		83		11.3		
		E4	**11232**			13.8		64.4	7.7
		E5	14104		11		83		
		E6	**17911**						50.8
	138.2	E1	**5093**	17.8			54.2	7.3	
		E2	**7689**	52.7			27.6	6.8	
		E3	**9405**	22.9				23.3	18.2
		E4	**11280**		87	4.4			
		E5	**15004**		7.3	76.8			
		E6	18242					28.7	
	169.7	E1	**5545**	94.3					
		E2	**8204**			78.2	5.9	6.8	
		E3	**8939**		79.3		7.5	6.1	
		E4	**12092**			12.2	36.1	37.3	
		E5	12924		12.5		36.9	41.2	
		E6	17417				6.4		72.3
	170.8	E1	**5851**	95.9					
		E2	8617		5.3	73		13.1	
		E3	8904		73.6	5.3	12.4		
		E4	12578			12.6	6.9	70.4	
		E5	12849		12.4		70.8	6.6	
		E6	17959						74.7
	172.1	E1	**5850**	95.8					
		E2	**8668**		14.8	63.6		9.4	
		E3	8842		64	14.9	9.2		
		E4	12639			11.7	19.5	57.7	
		E5	12794		11.5		58.4	19	
		E6	17958						74.5
	172.2	E1	**5387**	94.4					
		E2	**8112**			77.9	3.4	9.8	
		E3	**9011**		78		11.8	3.3	
		E4	**11953**			12.6	21.3	51.5	
		E5	**12735**		13.8		53	24.8	
		E6	17467				3.8		71
[**3**]$^+$	-173.9	E1	**5898**	86		7.8			
		E2	**8252**	6.9	10.3	70.9			
		E3	**9443**		71.2	4.9	12.4		
		E4	12474				43.3	38.3	5
		E5	**13574**		9		36.6	38.7	
		E6	17422					6.5	70.2
	-159.5	E1	**6658**	92.4					
		E2	**9235**		23.6	54.3	13.7		

		E3	**9665**		54.4	22.3		14.4	
		E4	13418			11.2	71.6	4.1	
		E5	**13895**		11		4.4	74	
		E6	**17506**						79.7
	-158.6	E1	**7251**	96.4					
		E2	9767		62.6	16.3	16.2		
		E3	9868		16.4	62.8		15.7	
		E4	14291		11.2	3.9	77.6		
		E5	14369		3.8	11		78	
		E6	17386						85.5
	-115.1	E1	**6875**	94.6					
		E2	9071			74.8		19.1	
		E3	**9786**		77.2		14.9		
		E4	13293			17.6		72.9	
		E5	**13984**		14.9		77.9		
		E6	**17590**						81.2
	-96.5	E1	**6308**	90.9					
		E2	8504		81.4		11.5		
		E3	**9514**	5.5		75.9		13.5	
		E4	12999		12		79.9		
		E5	**13573**			14.1		79.2	
		E6	17703						77.1
	-32.8	E1	**7103**	92.9					
		E2	**8627**			74.7		15.9	
		E3	**10115**		77.3		15.7		
		E4	13130			11.2	5.3	73	
		E5	**14059**		15.7	5.6	70.8		
		E6	18112						77.6
	41.7	E1	**6868**	95.1					
		E2	9370			73		17.8	
		E3	**9918**		74.7	3.6	13.5		
		E4	13631			16.7		75.1	
		E5	**14211**		14.2		78		
		E6	**17494**						82
	62.6	E1	**6873**	92.3					
		E2	**8631**		5.7	73		13.1	
		E3	**9903**		73.8	3.4	14.8		
		E4	13113			10.8	10.3	66.8	
		E5	**14079**		13.5		69.1	8.2	
		E6	17829						75.3
	119.4	E1	7384				78.2	16.7	
		E2	**8925**	79.1	12.4				
		E3	**9038**	15.3	69	9.6			
		E4	11813				12.6	73	
		E5	**14247**		14.5	80			
		E6	**18770**						68.2
	125.9	E1	**5766**	58.8		25.4	5		
		E2	**7701**	35.2		31.3	18.3		
		E3	**9886**		72.4		21.7		
		E4	**11720**		8.9		25.3	44.5	
		E5	**14392**			35.4	22.1	24.3	
		E6	**18010**					17.4	44.1
	146.2	E1	**6885**	92.4					
		E2	**9095**		62.7	15.6	13.9		
		E3	**9784**		14.5	62.9	13.9		
		E4	13393		13.6		73.7	2.8	
		E5	**14122**			12.3	2.8	76.6	
		E6	17338						80.1

	155.2	E1	**7134**	94					
		E2	8467				75.2	19.6	
		E3	**9895**		79.4	14.3			
		E4	12685				16.7	72.8	
		E5	**14009**		14.1	79.6			
		E6	**18185**						77.3
	160.2	E1	**6773**	94.1					
		E2	**8384**				79.5	14.1	
		E3	**10055**		80.4	13.4			
		E4	12595				11.1	77.3	
		E5	**13948**		13.4	79.8			
		E6	17621						78.7
[**4**]$^+$	-169.7	E1	**5881**	95.6					
		E2	8540		10.9	66.3	8		
		E3	**8818**		66.8	10.8		7.8	
		E4	12575			12.8	28.9	46.4	
		E5	12823		12.7		46.7	29.7	
		E6	17831						74.2
	-119.2	E1	**6250**	94.9					
		E2	7355			6.9	64.4	22	
		E3	**9765**		78.2	12.7			
		E4	10926				15.6	67.3	
		E5	**13421**		15.3	71.6	6.6		
		E6	**17829**						58.3
	-64.0	E1	**5230**	35.7		7.9	40.1		
		E2	**7373**	53.8			36		
		E3	**9830**		16.4			29.4	11.1
		E4	**10778**		64.6	3.1		15.7	
		E5	**13960**		13	71		4.4	
		E6	**18377**			4.9		25.6	
	-35.6	E1	**6299**	93.1					
		E2	**7530**			68.3	6.7	15.9	
		E3	**9695**		75.8		16		
		E4	11521			9.4	4	71.1	
		E5	**13131**		16.5	10.3	65.6		
		E6	**18159**						60.9
	27.1	E1	**6075**	95					
		E2	7823			76.3		16.7	
		E3	**9329**		76.3		16.3		
		E4	11866			13.7		73.7	
		E5	**13027**		15.9		76.2		
		E6	**17797**						69.5
	33.2	E1	**6029**	87.8		4.6			
		E2	**7472**			58.8	12.1	14	
		E3	**9494**		79.1		11.9		
		E4	11190			7.9	5.9	68	
		E5	**13389**		14.1	16.8	61.4		
		E6	**18080**						52.2
	99.2	E1	**5465**	92.3					
		E2	8034		70.7		21.1		
		E3	**8604**	3.5		75.9		13.6	
		E4	11515		21		68.8		
		E5	**12266**			13.4		77.9	
		E6	**17675**				3.2		72.2
	122.7	E1	**4915**	17.4		10.2	52.7		
		E2	**7808**	51.5			27.3	8.3	
		E3	**9420**	24.4				23.4	
		E4	**11191**		79.2			5.1	

		E5	**15001**		8.6	72.7	2.7		
		E6	**18257**					26.6	
	147.7	E1	**6293**	26.5			50.5	12.7	
		E2	**7169**	67.3			21.5		2.8
		E3	**10113**		36.1		11.4	27.3	
		E4	**10384**		43.7	4.7		31.9	
		E5	**13935**		14.1	77.3			
		E6	**18370**					12.8	26.5
	164.6	E1	**5737**	89		4.1			
		E2	**7317**	5.9		70		13.3	
		E3	**9889**		80		11.4		
		E4	**10847**			13.3		58.1	7.3
		E5	**13697**		10.5		74.9	6.5	
		E6	**17176**					10.3	54.6
[**5**]$^{+}$	-165.4	E1	**7457**	96.5					
		E2	9758			77.2	14.8		
		E3	9777		78			13.9	
		E4	14284			13.6	77.4		
		E5	14358		12.8			78.5	
		E6	17331						86.7
	-148.9	E1	**7036**	93					
		E2	**8838**		4.7	74.5		13.3	
		E3	**9691**		74.4	3.8	12.2		
		E4	13427			12.6	14.3	63	
		E5	**13876**		12.9		65.3	14.2	
		E6	17505						80.3
	-132.6	E1	**6627**	81		11.8			
		E2	**8071**	13.3		64.9		8.5	
		E3	**9879**		74.9		15.5		
		E4	**12472**			7.1	14.4	63.6	
		E5	**14098**		12.4		64.3	10.2	
		E6	17790					7.1	70.8
	-68.9	E1	**7070**	90.1					
		E2	**8339**			68.2	6.3	13.3	
		E3	**9716**		75.6	5.1	11.9		
		E4	12745			17.4	9.9	59.1	
		E5	**13875**		12.1		66.4	15.2	
		E6	18105						72.8
	-62.0	E1	**6927**	5.9			63.4	17.9	
		E2	**8701**	84.9			6.9		
		E3	**9241**		81	6.7	5.3		
		E4	**11327**				14.8	70.6	
		E5	**14226**	3.5	10.9	78.6			
		E6	18581						64.9
	-14.9	E1	**6874**	92.6					
		E2	**9055**		7.8	70.3		13.7	
		E3	**9646**		69.7	6.4	12.5		
		E4	13404			11.4	27.8	49.3	
		E5	**13780**		11.9		51.2	26.9	
		E6	17720						80.5
	28.4	E1	**7028**	93.2					
		E2	8718			76.7		15.9	
		E3	**9699**		77		14.5		
		E4	13106			15.1	14.3	59.6	
		E5	**13820**		13		64.4	16.1	
		E6	17953						79.8
	34.7	E1	**7037**	94.8					
		E2	9484		62.2	17	14.5		

		E3	**9792**		17.2	59.3		17	
		E4	13873			14.6	48.1	27.2	
		E5	14065		12.5		28.2	49.9	
		E6	17433						81.9
	104.6	E1	**6946**		3.2		73.5	14.9	
		E2	**8954**	81.4	4.8	4.6			
		E3	**9280**	10.2	74.2	6.1			
		E4	**11484**				10.4	69.6	
		E5	**14379**		12.3	78.7			
		E6	19071						9.3
	165.5	E1	**7028**	94.4					
		E2	**8622**				72.1	20.3	
		E3	**9909**		79.6	14.2			
		E4	12697				17.6	71.8	
		E5	**14114**		14.9	77.8			
		E6	17962						77.3
a) H = HOMO; b) S = SOMO									

Table S4. Orbital composition (β-spin) for conformers of [**1** – **5**]$^{+}$.

[**1**]$^{+}$	Ω	2.8			29.0			38.2			92.3			143.0			153.8		
	Orb	Ru	bridge	Ru	Ru	bridge	Ru	Ru	bridge	Ru	Ru	bridge	Ru	Ru	bridge	Ru	Ru	bridge	Ru
	H-6	10.4	8.8	10.4	9.1	8.9	12.1	9.7	7.9	9.6	5.8	16.6	50.1	9.0	8.1	11.0	6.9	11.8	17.9
	H-5	12.2	27.9	14.5	10.2	27.4	18.7	18.5	28.2	11.2	0.4	9.0	22.4	12.8	28.6	16.3	7.8	23.6	24.9
	H-4	7.8	29.6	34.3	40.6	29.6	0.7	1.5	29.9	40.3	38.3	28.8	0.3	40.6	29.7	0.5	39.5	29.8	0.6
	H-3	34.2	29.5	7.3	2.9	28.9	38.4	36.3	29.0	4.7	53.9	16.6	0.0	1.8	29.6	40.0	54.6	14.8	0.4
	H-2	53.4	13.7	2.4	55.1	14.7	0.2	1.4	13.8	54.5	6.0	26.5	33.5	55.2	14.3	0.3	5.5	27.6	33.5
	H-1	2.5	14.9	54.4	0.4	15.1	55.2	53.8	15.3	1.5	6.8	27.7	45.2	0.3	14.4	55.5	0.7	18.2	55.3
	H	22.3	38.7	23.7	19.2	39.2	26.4	24.1	38.7	21.8	1.0	30.2	48.6	20.7	39.3	24.7	14.3	37.5	31.7
	S	15.3	58.6	15.4	18.5	59.2	11.6	14.2	59.8	15.5	31.5	53.8	3.8	16.9	59.5	12.9	23.1	58.3	8.2
	Ω	155.1			164.3			168.5			179.3								
	Orb	Ru	bridge	Ru	Ru	bridge	Ru	Ru	bridge	Ru	Ru	bridge	Ru						
	H-6	6.5	12.0	19.2	9.9	10.4	10.8	10.0	7.1	10.0	9.9	7.1	10.1						
	H-5	7.3	23.3	25.2	18.2	26.0	11.6	14.6	29.5	14.6	14.0	29.6	14.3						
	H-4	40.0	29.7	0.2	11.7	30.1	30.3	21.9	30.1	20.4	20.4	29.9	21.5						
	H-3	54.4	15.2	0.1	26.0	28.6	11.0	19.4	29.3	20.8	21.2	29.4	19.8						
	H-2	5.1	27.7	33.7	0.5	14.5	55.1	29.6	12.8	26.4	26.6	12.9	29.3						
	H-1	0.7	18.1	55.5	56.0	14.1	0.4	26.4	15.3	29.7	29.3	15.3	26.7						
	H	13.7	37.5	32.3	25.1	37.8	20.9	22.7	39.4	22.7	22.8	39.3	22.7						
	S	23.8	57.9	7.8	13.9	58.5	16.9	14.9	59.4	14.9	15.0	59.0	15.0						
[**2**]$^{+}$	Ω	25.5			36.0			138.2			169.7			170.8			172.1		
	Orb	Ru	bridge	Ru	Ru	bridge	Ru	Ru	bridge	Ru	Ru	bridge	Ru	Ru	bridge	Ru	Ru	bridge	Ru
	H-6	46.3	10.5	2.6	11.8	9.9	11.3	6.5	10.9	40.1	13.5	7.7	6.3	10.6	5.4	10.5	10.4	5.4	10.6
	H-5	2.0	30.5	39.1	11.8	22.5	12.1	11.6	15.7	18.6	9.1	22.6	18.1	12.3	23.8	10.4	11.7	23.8	11.3
	H-4	21.2	16.7	4.7	3.1	31.4	37.1	16.8	25.2	18.9	20.5	31.4	22.6	1.4	31.2	41.8	4.7	31.1	38.2
	H-3	0.0	10.3	52.0	45.7	30.4	0.1	51.3	12.0	0.9	22.5	30.8	16.3	41.5	31.0	1.7	38.5	31.1	4.8
	H-2	37.6	31.3	3.6	0.1	10.7	52.7	4.2	32.3	36.7	0.2	10.3	54.5	0.2	9.7	54.4	0.9	9.7	53.8
	H-1	55.3	12.6	0.3	56.0	9.9	0.0	0.2	12.1	55.1	55.1	10.0	0.2	54.5	9.8	0.2	53.8	10.1	1.0
	H	37.9	34.6	9.7	28.6	38.5	17.1	7.7	34.7	39.1	24.1	38.1	22.2	23.5	38.9	22.2	23.2	39.0	22.5

	S	6.4	49.0	32.7	10.5	52.9	25.0	38.9	44.4	4.5	16.0	54.1	18.5	16.5	53.9	17.9	16.8	53.9	17.6
	Ω	172.2																	
	Orb	Ru	bridge	Ru															
	H-6	10.8	8.1	7.8															
	H-5	9.4	22.1	16.7															
	H-4	11.4	31.6	30.8															
	H-3	33.4	31.2	8.3															
	H-2	0.1	10.1	54.8															
	H-1	54.3	10.1	0.2															
	H	24.9	37.9	21.6															
	S	15.6	54.1	19.0															
[**3**]$^+$	Ω	-173.9			-159.5			-158.6			-115.1			-96.5			-32.8		
	Orb	Ru	bridge	Ru	Ru	bridge	Ru	Ru	bridge	Ru	Ru	bridge	Ru	Ru	bridge	Ru	Ru	bridge	Ru
	H-6	10.7	10.9	10.2	9.6	9.0	9.8	9.7	8.1	9.5	7.8	10.1	11.5	9.6	8.6	9.6	16.8	10.3	5.3
	H-5	16.8	25.2	14.7	18.2	28.6	15.3	15.6	30.3	15.0	10.6	28.0	22.6	16.3	25.3	16.2	24.0	27.1	9.4
	H-4	12.2	28.7	28.7	18.4	30.0	23.1	20.7	29.7	21.0	40.3	29.6	0.4	20.7	30.9	21.1	0.9	29.2	39.8
	H-3	23.7	21.7	15.2	19.3	26.7	18.9	20.5	29.6	20.5	5.0	28.6	35.1	20.3	24.2	19.9	31.2	27.3	9.0
	H-2	4.0	21.0	50.2	55.7	16.0	0.4	18.3	14.6	37.3	55.7	15.0	0.1	24.7	13.6	30.2	1.9	16.8	51.9
	H-1	56.3	17.0	0.5	0.4	15.4	54.9	37.7	15.0	18.5	0.5	15.9	54.6	29.9	19.7	24.3	55.7	16.3	0.4
	H	26.8	36.3	19.1	22.4	38.6	23.1	22.4	39.4	22.4	19.0	38.6	26.5	22.9	38.4	22.8	29.0	38.1	16.5
	S	11.3	59.2	18.6	14.6	61.1	13.3	13.9	60.7	13.9	16.8	61.2	11.0	13.8	61.4	14.0	9.1	60.4	19.4
	Ω	41.7			62.6			119.4			125.9			146.2			155.2		
	Orb	Ru	bridge	Ru	Ru	bridge	Ru	Ru	bridge	Ru	Ru	bridge	Ru	Ru	bridge	Ru	Ru	bridge	Ru
	H-6	9.9	8.5	9.5	14.4	11.1	6.6	0.2	9.7	22.6	19.2	10.7	9.3	7.1	12.0	10.8	17.6	10.0	4.9
	H-5	21.9	29.1	10.8	20.5	26.6	11.5	8.7	26.8	37.2	17.9	20.9	13.5	21.5	24.5	11.9	26.5	27.3	9.0
	H-4	0.0	29.7	40.9	3.2	29.2	37.7	38.9	28.7	0.0	18.7	26.9	23.2	15.2	30.9	26.7	0.1	29.6	40.0
	H-3	35.6	28.5	4.8	34.0	26.6	7.0	54.0	17.4	0.0	11.2	12.5	36.6	22.4	27.9	15.8	0.2	15.8	55.0
	H-2	0.3	14.7	56.0	1.2	16.5	53.1	6.6	22.6	33.9	17.6	26.4	28.5	1.3	15.7	53.9	32.2	26.1	6.6
	H-1	54.9	15.7	0.5	54.4	16.7	0.8	9.9	30.5	40.6	52.1	16.9	1.6	55.1	15.1	0.6	54.6	19.4	0.9
	H	24.9	38.6	20.5	28.2	38.3	17.4	0.2	29.6	50.0	32.1	31.1	14.7	22.1	39.0	23.1	30.7	37.3	15.1
	S	12.5	61.1	15.4	10.0	60.2	18.7	26.7	58.0	4.4	7.7	52.8	23.1	13.0	62.3	13.6	8.4	60.8	20.2
	Ω	160.2																	
	Orb	Ru	bridge	Ru															
	H-6	6.6	12.6	14.3															

	H-5	6.5	25.5	24.2															
	H-4	41.3	27.1	0.0															
	H-3	55.4	15.7	0.2															
	H-2	6.0	26.1	31.5															
	H-1	0.4	15.8	56.4															
	H	16.0	31.3	30.1															
	S	21.6	51.6	9.1															
**[4]**$^{+}$	Ω	-169.7			-119.2			-64.0			-35.6			27.1			33.2		
	Orb	Ru	bridge	Ru	Ru	bridge	Ru	Ru	bridge	Ru	Ru	bridge	Ru	Ru	bridge	Ru	Ru	bridge	Ru
	H-6	10.4	5.6	10.3	12.1	11.9	11.1	12.8	15.5	16.3	13.5	10.0	11.0	10.2	8.2	10.7	12.9	10.7	10.9
	H-5	11.5	25.8	11.1	18.4	21.0	5.2	16.6	13.2	9.3	18.6	20.4	5.4	15.5	22.9	7.9	7.0	19.3	18.1
	H-4	10.1	30.4	32.8	0.1	31.5	40.4	14.2	27.5	23.3	0.7	30.2	40.3	0.2	30.6	41.4	38.1	30.1	2.2
	H-3	32.4	30.1	9.4	3.4	14.4	49.9	1.3	12.9	51.8	33.2	29.4	9.6	40.7	31.3	1.6	13.9	26.0	30.0
	H-2	0.4	12.1	54.9	35.9	30.3	6.4	33.6	33.1	5.1	5.2	16.8	47.2	0.1	12.5	54.4	43.0	18.5	8.5
	H-1	54.7	12.3	0.4	55.6	11.0	0.2	56.2	12.5	0.1	55.8	10.9	0.0	54.9	11.0	0.2	0.2	12.2	55.2
	H	23.5	38.6	22.5	30.6	38.3	15.3	34.2	37.1	11.0	29.6	38.5	16.2	27.3	38.3	18.5	15.9	38.2	30.0
	S	16.6	53.9	17.8	9.8	53.0	25.5	6.4	48.0	33.1	10.4	53.8	24.2	12.8	54.1	21.2	24.9	53.5	10.3
	Ω	99.2			122.7			147.7			164.6								
	Orb	Ru	bridge	Ru	Ru	bridge	Ru	Ru	bridge	Ru	Ru	bridge	Ru						
	H-6	9.1	7.6	9.1	11.4	15.4	32.2	29.0	12.8	7.6	12.1	11.5	9.2						
	H-5	12.9	19.8	12.8	5.1	12.0	25.3	17.9	17.3	8.4	13.0	24.0	13.2						
	H-4	21.2	32.4	22.1	18.5	25.3	20.0	4.6	28.9	33.9	6.8	31.5	32.0						
	H-3	20.1	31.8	19.3	50.7	14.4	2.3	0.6	13.1	52.7	37.5	29.1	3.7						
	H-2	14.6	10.4	40.3	5.4	32.4	34.0	37.9	31.2	3.7	0.3	13.0	53.9						
	H-1	40.3	11.4	14.7	0.8	15.1	54.1	55.2	13.2	0.5	55.9	11.7	0.2						
	H	23.2	36.0	23.0	6.9	34.6	39.6	36.8	35.9	10.3	30.5	36.3	16.3						
	S	16.2	51.7	16.3	38.6	44.7	4.4	7.1	50.0	31.2	11.2	51.8	25.3						
**[5]**$^{+}$	Ω	-165.4			-148.9			-132.6			-68.9			-62.0			-14.9		
	Orb	Ru	bridge	Ru	Ru	bridge	Ru	Ru	bridge	Ru	Ru	bridge	Ru	Ru	bridge	Ru	Ru	bridge	Ru
	H-6	8.9	10.0	8.8	7.6	12.5	10.4	13.7	10.1	6.9	15.9	10.7	5.5	21.8	11.0	1.1	8.4	13.1	9.9
	H-5	16.4	28.6	16.2	11.8	25.7	23.7	19.5	24.4	15.4	21.3	24.5	13.9	35.7	22.8	10.3	8.9	24.4	25.5
	H-4	17.1	31.0	23.8	37.4	31.3	2.4	6.7	30.8	32.6	5.0	31.3	34.9	1.4	31.0	36.9	38.4	30.6	2.4
	H-3	23.8	31.0	17.0	5.8	28.6	33.1	30.1	23.5	11.1	30.9	27.5	8.0	0.6	15.3	53.9	7.2	29.0	31.6
	H-2	8.8	14.3	47.0	55.2	15.0	0.3	3.2	17.8	51.1	0.5	14.8	54.1	32.5	23.9	7.2	54.7	15.4	0.4

	H-1	47.0	14.3	8.7	0.6	15.1	56.0	53.8	18.5	1.1	54.8	18.7	0.8	47.1	27.8	5.3	0.2	16.1	55.7
	H	22.1	40.9	22.0	18.4	40.3	26.5	30.4	38.4	15.5	30.0	38.4	15.6	44.1	32.1	4.9	19.3	39.6	25.8
	S	13.1	62.8	13.2	16.0	63.4	10.5	8.9	62.1	19.1	8.9	62.5	18.5	4.3	59.1	26.2	16.0	63.2	10.7
	Ω	28.4			34.7			104.6			165.5								
	Orb	Ru	bridge	Ru	Ru	bridge	Ru	Ru	bridge	Ru	Ru	bridge	Ru						
	H-6	5.0	9.7	15.1	8.6	9.6	9.9	42.3	21.5	8.3	6.5	11.4	15.3						
	H-5	13.1	26.4	21.8	19.8	28.2	13.0	22.4	11.0	1.1	9.2	27.2	23.9						
	H-4	37.7	31.2	2.7	12.7	31.4	28.0	2.6	28.0	36.9	39.0	28.1	0.0						
	H-3	6.6	27.4	31.9	25.8	29.7	13.4	0.2	18.3	53.1	54.2	18.5	1.0						
	H-2	55.2	14.7	0.0	0.3	14.2	55.6	32.8	25.0	6.5	5.5	27.5	34.7						
	H-1	0.6	17.6	55.1	55.9	14.6	0.3	44.1	29.6	6.9	0.7	18.0	54.5						
	H	17.0	38.9	28.3	22.0	40.2	22.5	48.6	29.7	2.1	16.5	39.2	28.8						
	S	17.1	62.7	10.1	12.8	62.2	14.3	3.9	56.6	29.0	20.1	60.9	8.7						

Table S5. Calculated vibrational frequencies for conformers of **[1 – 5]**$^+$ †

Compound	Angles			Stretches and their relative intensities											
	Ω (Cp)	θ_1	θ_2	s	Rel. Int.[1]	a	Rel. Int.	sca	Rel. Int.	CHb	Rel. Int.	CHb	Rel. Int.	Cp*	Rel. Int.
	°	°	°	cm^{-1}	%	cm^{-1}	%	cm^{-1}	%	cm^{-1}	%	cm^{-1}	%	cm^{-1}	%
**[1]**$^+$	2.8	-1.3	3.9	2013	0	1978	100			1463	3			1436	1
	29.0	2.8	-31.9	2012	0	1975	100	1585	2	1463	2			1425	1
	38.2	13.6	21.8	2011	5	1966	100			1477	1	1438	1		
	92.3	-0.5	-87.3	1966	59	2040	100	1556	75	1465	1				
	143.0	3.3	23.6	2014	0	1974	100	1587	1	1464	2			1438	1
	153.8	7.7	-43.9	1989	100	1993	60	1570	33	1465	2				
	155.1	9.8	-44.6	1989	39	1998	100	1568	35	1466	1			1438	1
	164.3	-11.1	-12.7	2013	0	1976	100	1586	1	1464	2			1437	1
	168.5	1.8	1.9	2016	0	1977	100			1463	2			1438	1
	179.3	0.6	-0.6	2016	0	1980	100			1463	3			1437	1
	Ω (Cp)	θ_1	θ_2	s	Rel. Int.	a	Rel. Int.[1]	sca	Rel. Int.	sca	Rel. Int.	aca	Rel. Int.	aca (tw)	Rel. Int.
	°	°	°	cm^{-1}	%	cm^{-1}	%	cm^{-1}	%	cm^{-1}	%	cm^{-1}	%	cm^{-1}	%
**[2]**$^+$	25.5	11.9	-52.4	1938	100	2024	88	1600	11	1594	47			1504	2
	36.0	17.4	15.5	1969	100	2007	25	1614	29			1520	1	1510	1
	138.2	-18.7	-38.9	1934	100	2039	54			1591	54			1499	3
	169.7	-5.8	-12.8	2007	7	2013	100	1626	1					1522	1
	170.8	3.5	-0.4	2011	10	2015	100	1625	0					1521	1
	172.1	1.1	1.0	2011	0	2016	100	1625	0					1521	1
	172.2	-3.4	-17.9	2003	3	2007	100	1626	2					1522	1
	Ω (Cp)	θ_1	θ_2	s	Rel. Int.[1]	a	Rel. Int.	sca	Rel. Int.	sca	Rel. Int.	aca	Rel. Int.	aca (tw)	Rel. Int.
	°	°	°	cm^{-1}	%	cm^{-1}	%	cm^{-1}	%	cm^{-1}	%	cm^{-1}	%	cm^{-1}	%
**[3]**$^+$	-173.9	29.6	-28.8	2016	0	1974	100								
	-159.5	-20.0	30.6	2021	0	1967	100								
	-158.6	6.4	9.1	2021	0	1973	100								
	-115.1	9.9	38.8	2013	7	1970	100	1595	3						
	-96.5	38.0	37.9	2022	0	1964	100								

	-32.8	13.6	-44.6	2013	2	1981	100	1590	7						
	41.7	13.0	24.6	2014	4	1964	100	1596	1						
	62.6	17.7	41.7	2015	2	1978	100	1590	5						
	119.4	12.9	-81.2	1981	23	2014	100	1574	35						
	125.9	-24.0	-41.2	1996	2	1969	100	1582	1	1574	20				
	146.2	-24.5	-17.7	2025	0	1964	100								
	155.2	12.0	-47.8	2004	26	1982	100	1588	10						
	160.2	2.1	-28.7	2003	0	1986	100	1586	9	1581	2				
	Ω (Cp)	θ_1	θ_2	s	Rel. Int.	a	Rel. Int. [1]	sca	Rel. Int.	sca	Rel. Int.	aca (tw)	Rel. Int.	aca (tw)	Rel. Int.
	°	°	°	cm^{-1}	%	cm^{-1}	%	cm^{-1}	%	cm^{-1}	%	cm^{-1}	%	cm^{-1}	%
**[4]**$^+$	-169.7	1.6	1.8	1997	3	2011	100					1489	9		
	-119.2	10.8	34.5	1963	74	2016	100	1591	28			1483	12		
	-64.0	-17.3	-47.8	1929	100	2026	53	1574	41			1493	4	1475	14
	-35.6	1.0	-35.0	1974	78	2014	100	1594	24			1480	11		
	27.1	-3.5	29.4	1989	55	2011	100	1600	10			1489	4	1481	7
	33.2	4.8	32.5	1959	50	2014	100	1592	20			1479	11		
	99.2	-34.1	-34.3	2004	5	2008	100					1487	7		
	122.7	-22.6	-42.0	1922	100	2019	39	1575	39			1500	9		
	147.7	-4.0	-37.3	1939	100	2022	63	1577	35			1477	16		
	164.6	-5.4	-19.3	1962	100	2012	69	1592	33			1481	12		
	Ω (Cp)	θ_1	θ_2	s	Rel. Int. [1]	a	Rel. Int.	sca	Rel. Int.	sca	Rel. Int.	aca	Rel. Int.	aca (tw)	Rel. Int.
	°	°	°	cm^{-1}	%	cm^{-1}	%	cm^{-1}	%	cm^{-1}	%	cm^{-1}	%	cm^{-1}	%
**[5]**$^+$	-165.4	5.4	5.5	2028	0	1971	100								
	-148.9	11.5	26.0	2024	0	1963	100	1590	3						
	-132.6	15.0	41.1	2011	2	1973	100	1583	3	1579	4				
	-68.9	-17.2	-46.9	2019	4	1982	100	1586	6						
	-62.0	13.4	-76.8	1989	37	2014	100	1567	67						
	-14.9	15.7	-27.3	2025	0	1963	100	1591	2						
	28.4	-15.0	41.5	2022	3	1975	100	1589	4	1581	1				
	34.7	19.1	13.6	2025	0	1968	100	1566	49						
	104.6	11.2	-89.9	1979	44	2024	100								
	165.5	10.7	-41.2	2010	14	1982	100	1584	5	1580	3				

† a: asymmetric, s: symmetric, sca: symmetric arene, aca: asymmetric arene, aca (tw): asymmetric arene twist; CHb: C-H bending (bridge, ca), Cp*: Cp*-breathing mode; [1]: Relative to highest intensity of conformer; IR-frequencies are scaled by a factor of 0.95.

Table S6. Summary of IR data from **[1a**,**b – 5a**,**b]**$^{+}$

	n	ν(C≡C) / cm^{-1}	ν(Aryl) /cm^{-1}
[**1a**]$^{n+}$	0	2069, 2043	1592
	+1	2065, 2002, 1976, 1929, 1891	1587, 1565
	+2	2065, 2002, 1976, 1928	-
[**2a**]$^{n+}$	0	2071, 2042	-
	+1	2113, 2061, 2046, 1992, 1936, 1897	1619, 1604, 1587, 1574
	+2	2035, 1991, 1924, 1896	1528
[**3a**]$^{n+}$	0	2123, 2086, 2064, 2054	-
	+1	2098, 2053, 1972, 1939, 1911	1580, 1573
	+2	1932, 1922, 1911	-
[**4a**]$^{n+}$	0	2056, 2048, 2019	-
	+1	2055, 2039, 1984, 1920	1584, 1573
	+2	1985, 1917	-
[**5a**]$^{n+}$	0	2055, 2015	-
	+1	2056, 2038, 1971, 1965, 1926, 1900	1587, 1573
	+2	1976, 1961, 1920	-
[**1b**]$^{n+}$	0	2073, 2045	-
	+1	2072, 2007, 1975, 1919	1569, 1566
	+2	1935	-
[**2b**]$^{n+}$	0	2077, 2047, 2021	-
	+1	2076, 2058, 2015, 1994, 1947	1629, 1606, 1587, 1573
	+2	2058, 1994	1631, 1620
[**3b**]$^{n+}$	0	2077, 2066	-
	+1	2106, 2059, 1986, 1970, 1944, 1909	1585, 1573
	+2	1929	1585, 1573
[**4b**]$^{n+}$	0	2061, 2030	-
	+1	2057, 1992, 1948	1585, 1573
	+2	2057, 1992, 1948	-
[**5b**]$^{n+}$	0	2071, 2038	-
	+1	2089, 2059, 2039, 1974, 1965, 1931, 1898, 1816	1587, 1576, 1572
	+2	1976, 1964, 1927	-

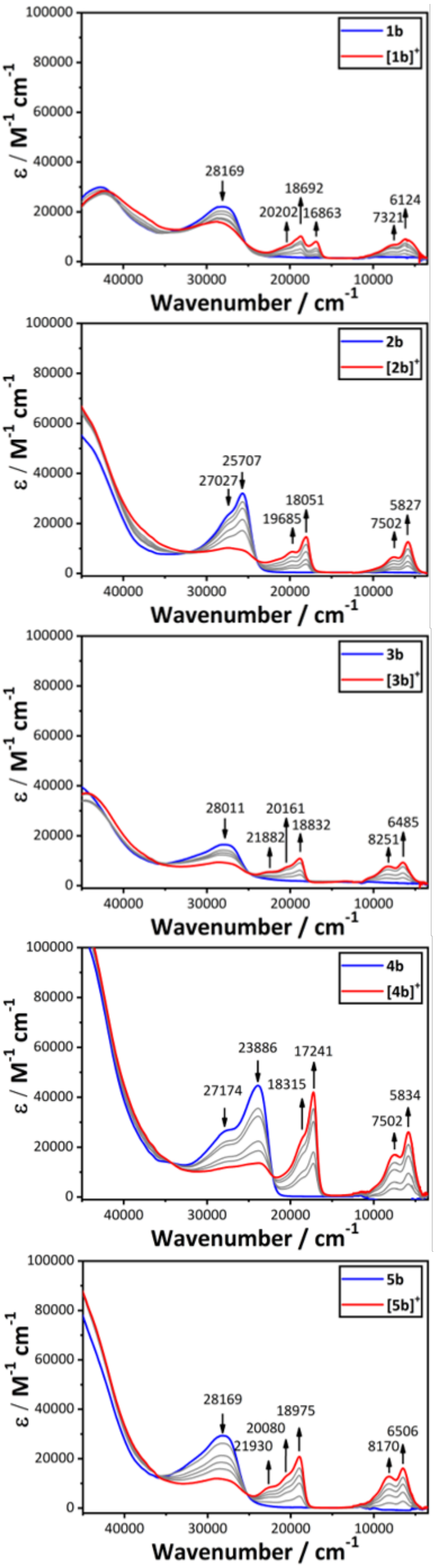

Figure S2. Plots of the UV-vis-NIR spectra of [**1b** – **5b**]$^{+}$ from spectroelectrochemical data.

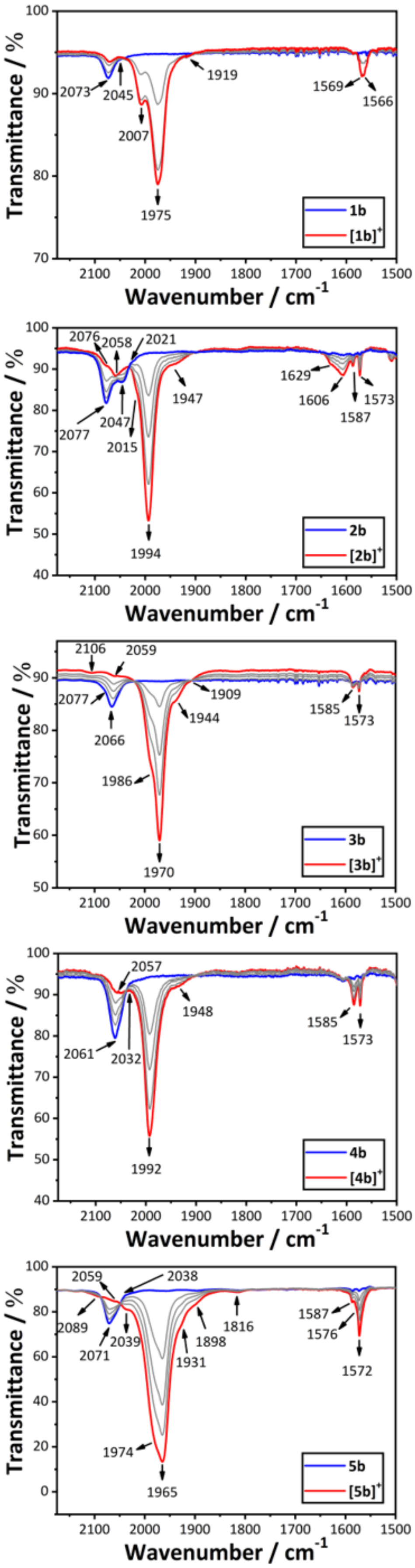

Figure S3. Plots of the spectroelectrochemically generated IR spectra of [**1b** – **5b**]$^+$

[**1**]$^{+}$		
2.8°	29.0°	38.2°
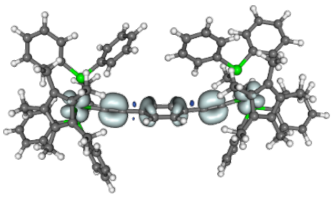	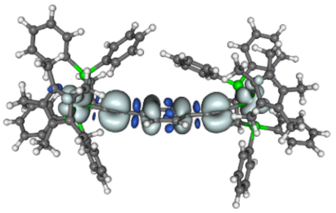	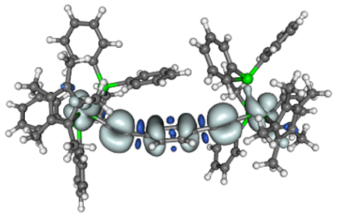
92.3°	143.0°	153.8°
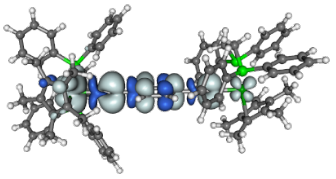	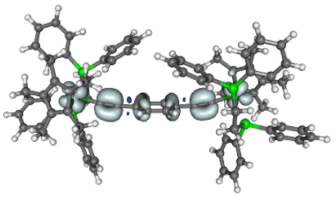	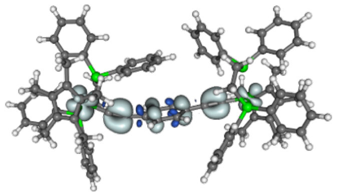
155.1°	164.3°	168.5°
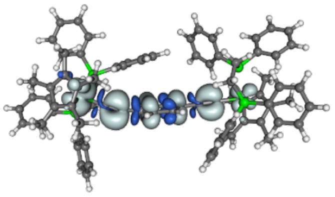	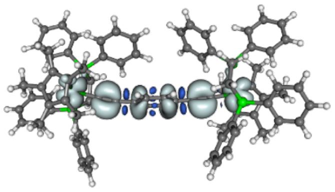	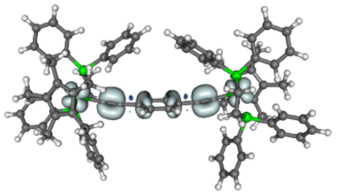
179.3°		
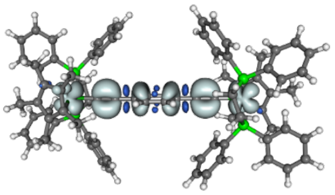		

Figure S4: Plots of spin density distributions of each conformer of [**1**]$^{+}$

$[\mathbf{2}]^+$		
25.5°	36.0°	138.2°
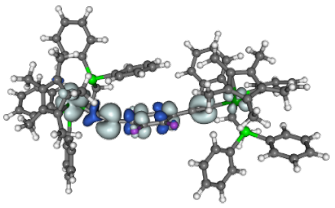	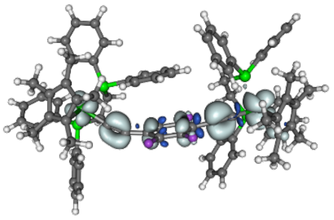	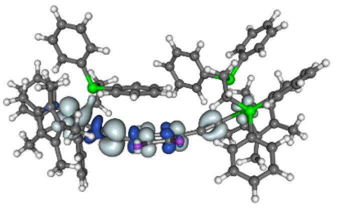
169.7°	170.8	172.1
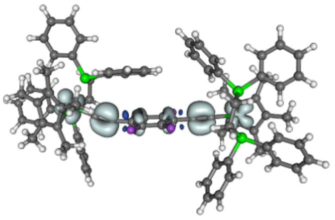	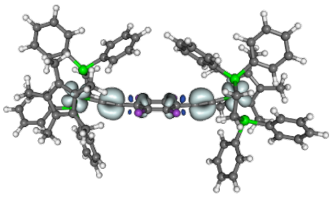	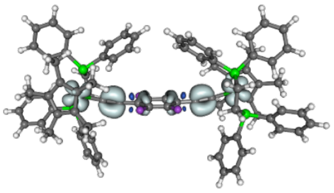
172.2°		
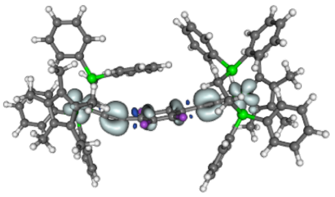		

Figure S5: Plots of spin density distributions of each conformer of $[\mathbf{2}]^+$

[**3**]$^{+}$		
-173.9°	-159.5°	-158.6°
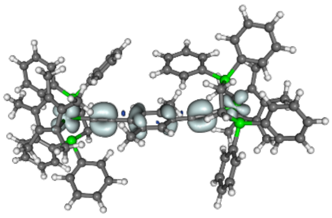	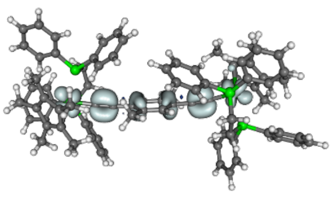	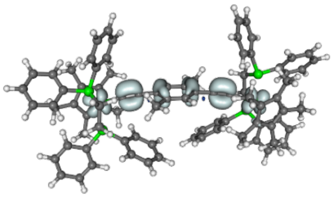
-115.1°	-96.5°	-32.8°
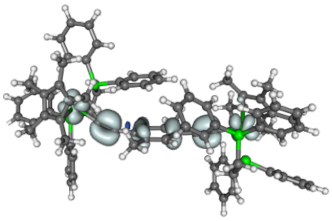	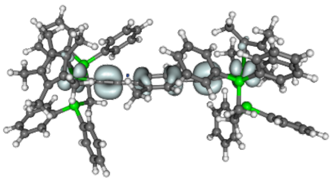	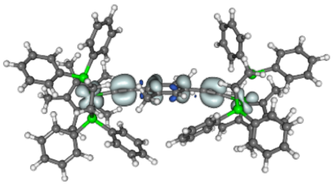
41.7°	62.6°	119.4°
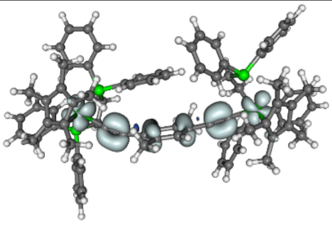	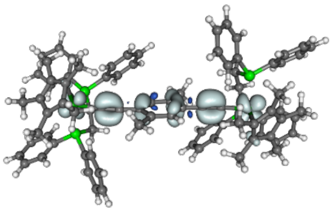	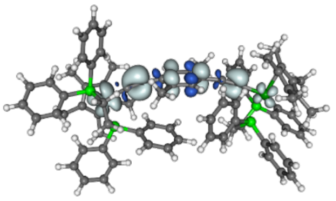
125.9°	146.2°	155.2°
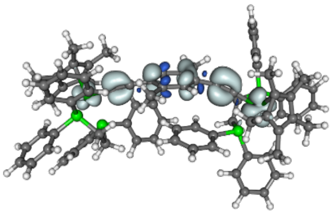	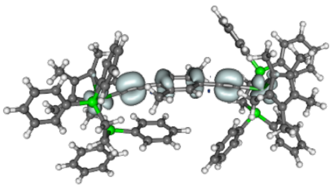	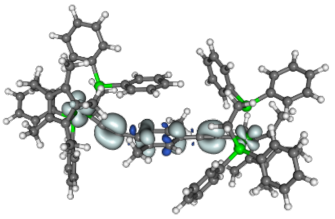
160.2°		
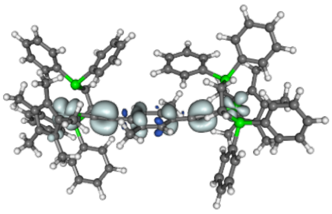		

Figure S6: Plots of spin density distributions of each conformer of [**3**]$^{+}$

[**4**]$^+$		
-169.7°	-119.2°	-64.0°
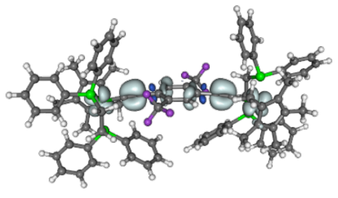	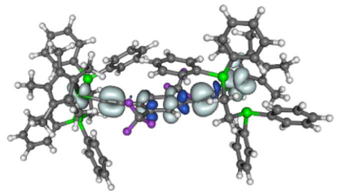	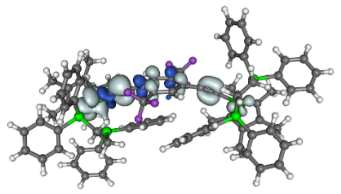
-35.6°	27.1°	33.2°
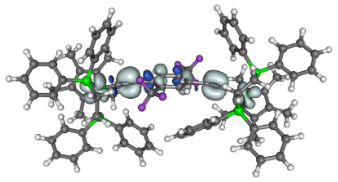	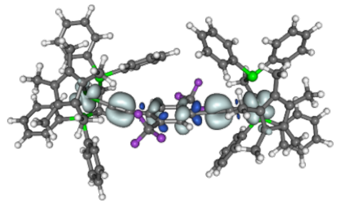	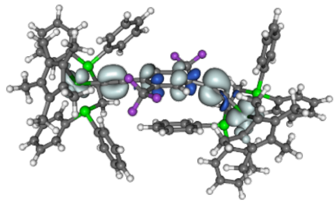
99.2°	122.7°	147.7°
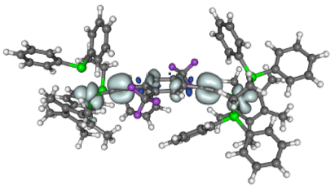	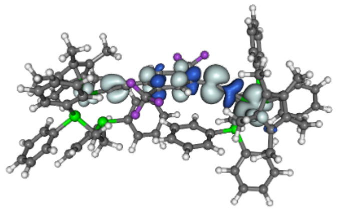	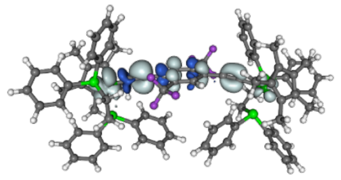
164.6°		
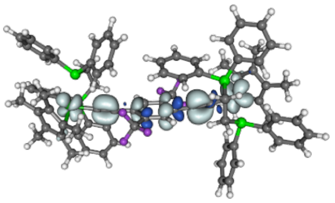		

Figure S7: Plots of spin density distributions of each conformer of [**4**]$^+$

[**5**]$^{+}$		
-165.4°	-148.9°	-132.6°
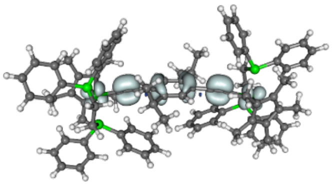	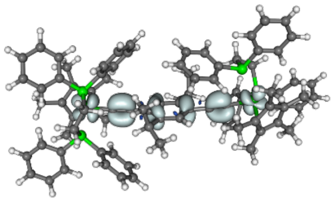	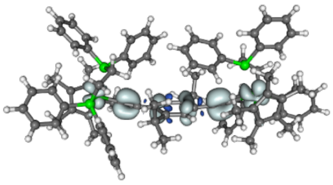
-68.9°	-62.0°	-14.9°
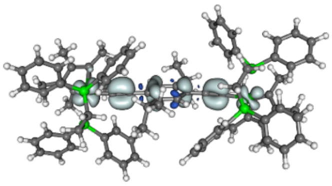	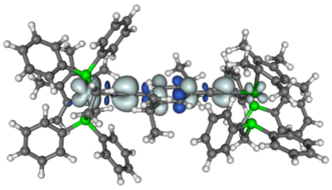	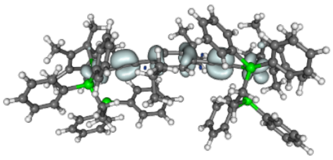
28.4°	34.7°	104.6°
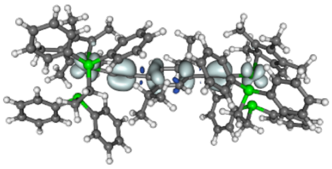	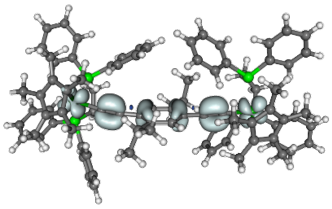	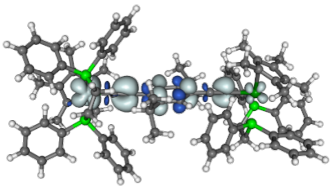
165.5°		
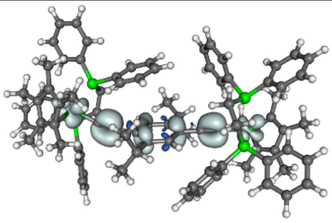		

Figure S8: Plots of spin density distributions of each conformer of [**5**]$^{+}$

	$[\mathbf{1}]^{+}$		
	2.8°	29.0°	38.2°
SOMO	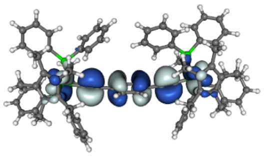	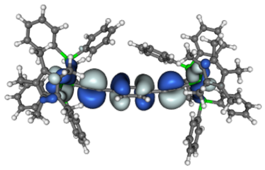	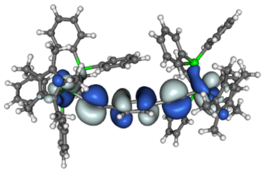
ε / eV	–3.38	–3.37	–3.39
HOMO	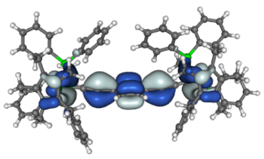	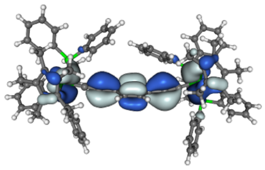	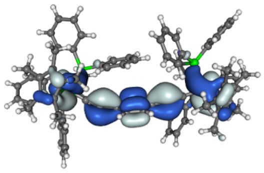
ε / eV	–5.42	–5.43	–5.44
HOMO-1	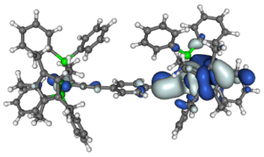	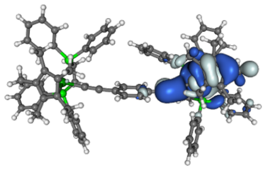	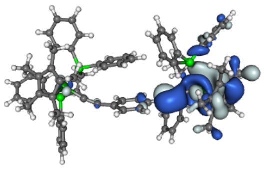
ε / eV	–6.13	–6.02	–6.12
HOMO-2	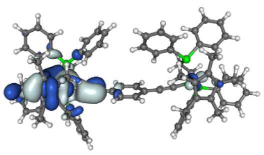	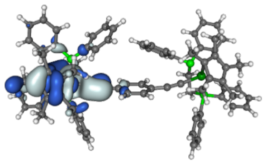	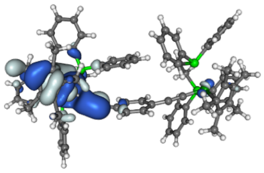
ε / eV	–6.16	–6.24	–6.18
HOMO-3	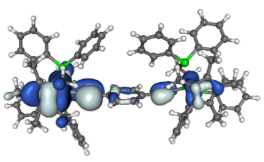	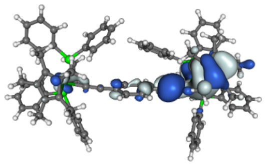	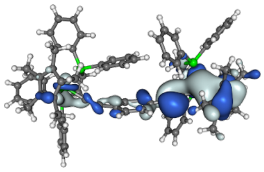
ε / eV	–6.59	–6.45	–6.52
	92.3°	143.0°	153.8°
SOMO	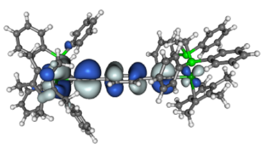	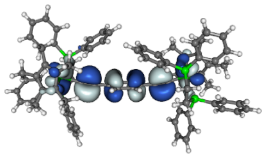	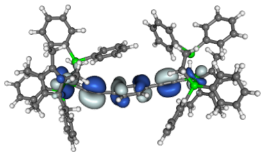
ε / eV	–3.39	–3.36	–3.39
HOMO	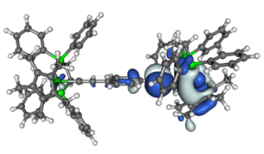	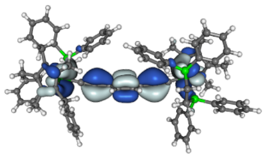	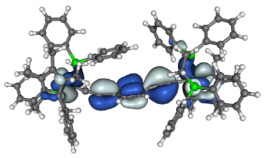

ε / eV	–5.57	–5.43	–5.45
HOMO-1	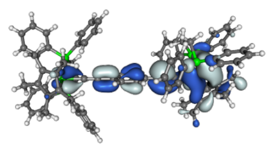	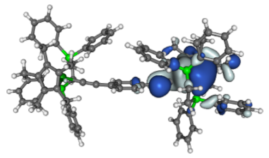	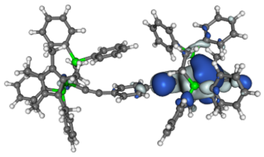
ε / eV	–5.62	–6.08	–5.90
HOMO-2	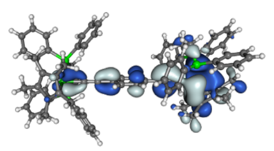	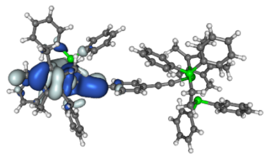	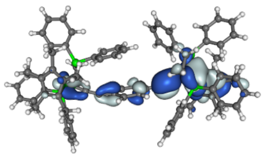
ε / eV	–6.08	–6.20	–6.27
HOMO-3	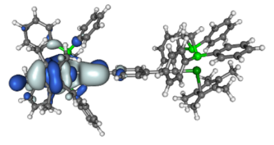	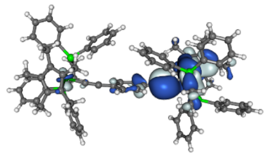	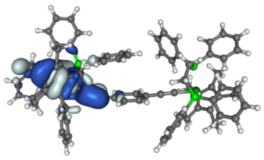
ε / eV	–6.56	–6.51	–6.36
	155.1°	164.3°	168.5°
SOMO	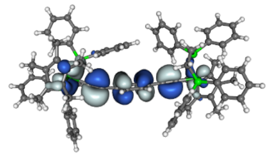	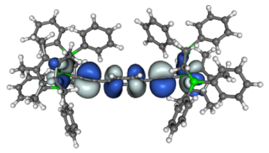	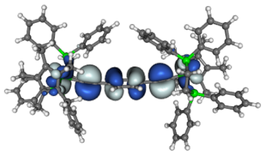
ε / eV	–3.39	–3.40	–3.36
HOMO	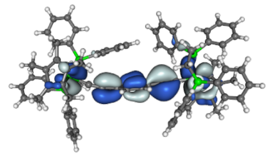	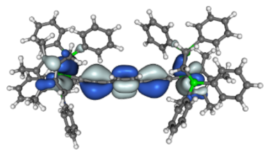	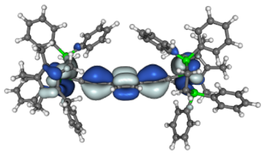
ε / eV	–5.44	–5.42	–5.43
HOMO-1	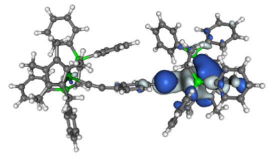	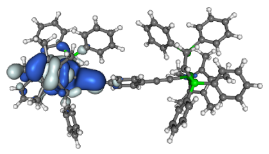	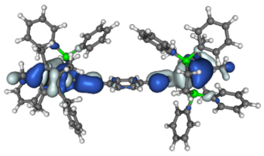
ε / eV	–5.88	–6.07	–6.14
HOMO-2	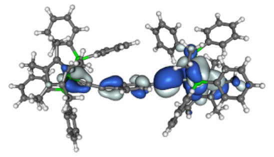	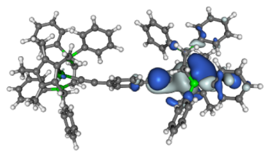	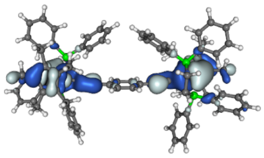

ε / eV	–6.25	–6.20	–6.15
HOMO-3	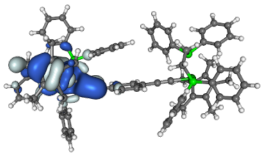	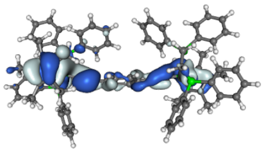	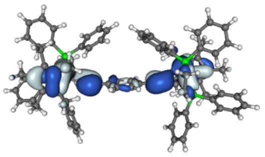
ε / eV	–6.38	–6.49	–6.56
	179.3°		
SOMO	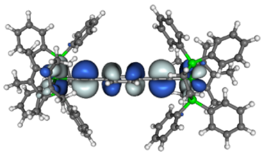		
ε / eV	–3.36		
HOMO	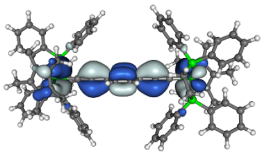		
ε / eV	–5.42		
HOMO-1	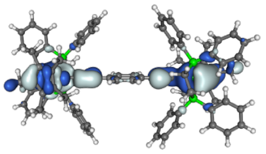		
ε / eV	–6.14		
HOMO-2	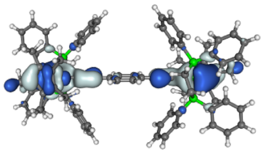		
ε / eV	–6.15		
HOMO-3	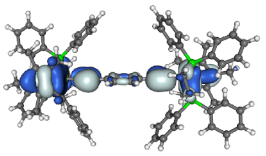		
ε / eV	–6.56		

Figure S9: Plots and energies (eV) of frontier orbitals of each conformer of [**1**]$^{+}$

	$[\mathbf{2}]^{+}$		
	25.5°	36.0°	138.2°
SOMO	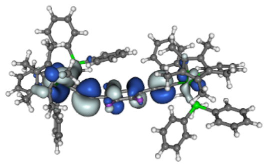	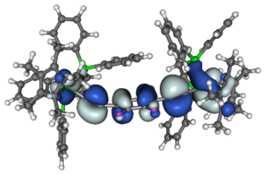	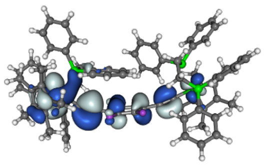
ε / eV	–3.59	–3.58	–3.58
HOMO	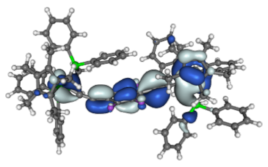	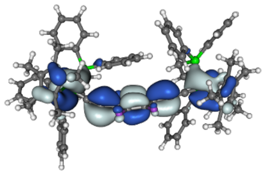	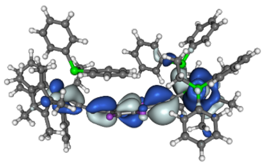
ε / eV	–5.58	–5.59	–5.58
HOMO-1	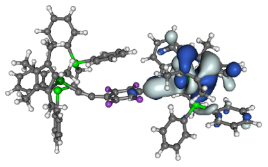	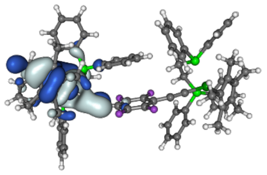	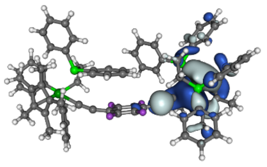
ε / eV	–5.94	–6.14	–5.88
HOMO-2	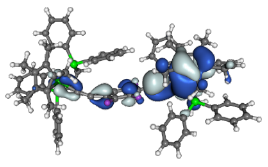	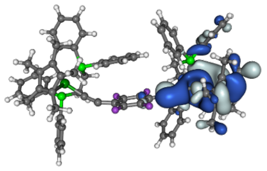	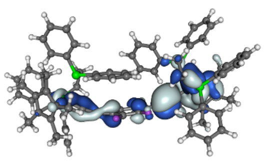
ε / eV	–6.42	–6.49	–6.42
HOMO-3	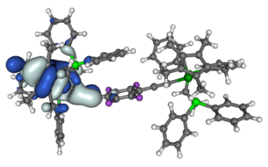	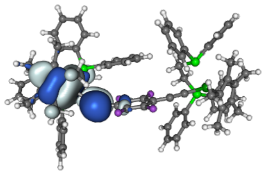	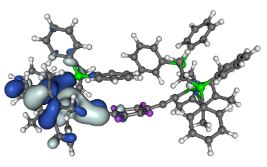
ε / eV	–6.69	–6.65	–6.76
	169.7°	170.8	172.1
SOMO	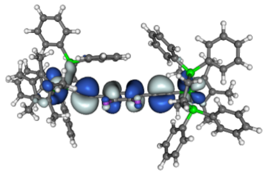	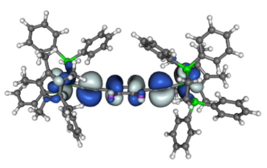	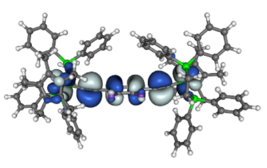
ε / eV		–3.58	–3.59
HOMO	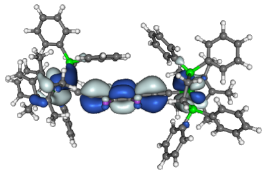	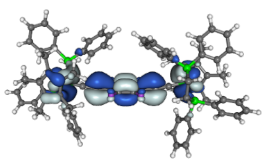	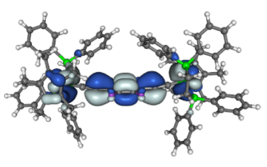

ε / eV		–5.58	–5.56
HOMO-1	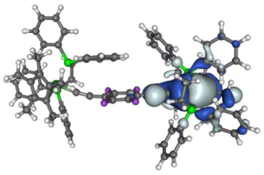	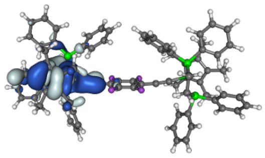	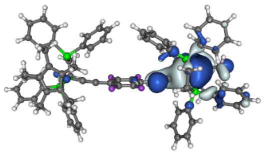
ε / eV		–6.27	–6.25
HOMO-2	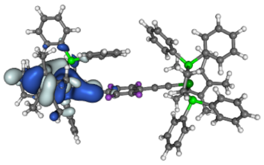	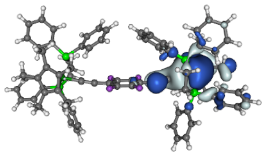	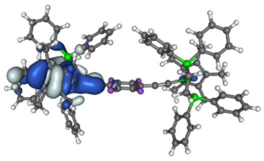
ε / eV		–6.31	–6.31
HOMO-3	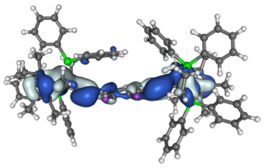	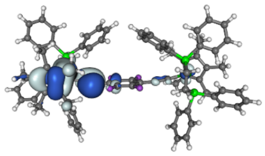	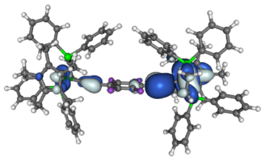
ε / eV		–6.75	–6.68
	172.2°		
SOMO	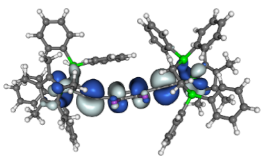		
ε / eV	–3.59		
HOMO	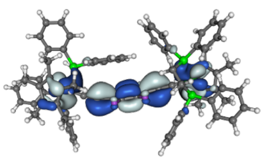		
ε / eV	–5.56		
HOMO-1	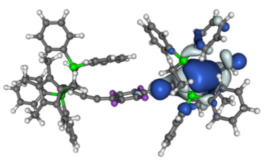		
ε / eV	–6.25		
HOMO-2	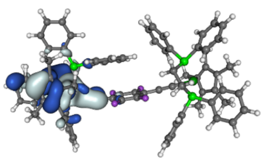		

ε / eV	–6.31		
HOMO-3	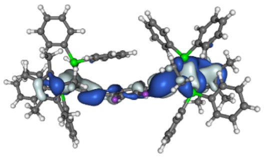		
ε / eV	–6.68		

Figure S10: Plots and energies (eV) of frontier orbitals of each conformer of [**2**]$^{+}$

	$[\mathbf{3}]^{+}$		
	-173.9°	-159.5°	-158.6°
SOMO	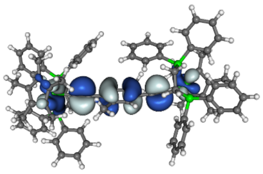	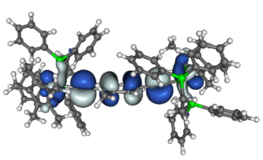	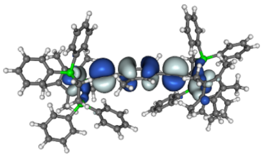
ε / eV	–3.40	–3.34	–3.30
HOMO	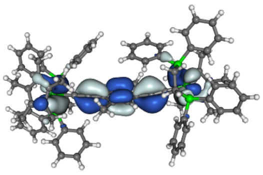	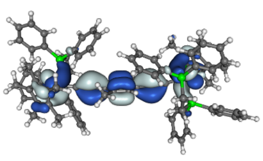	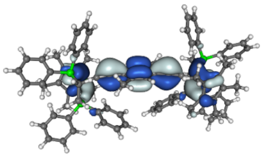
ε / eV	–5.42	–5.45	–5.45
HOMO-1	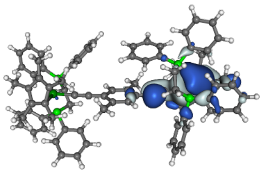	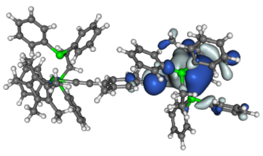	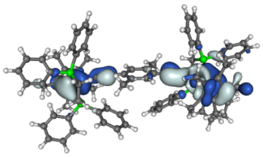
ε / eV	–5.99	–6.08	–6.11
HOMO-2	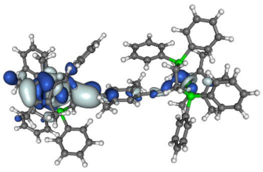	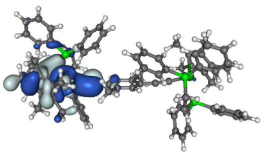	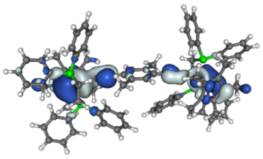
ε / eV	–6.19	–6.13	–6.11
HOMO-3	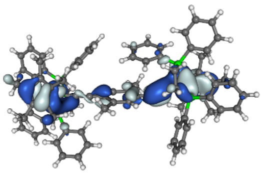	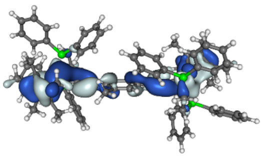	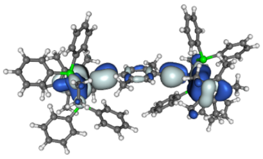
ε / eV	–6.32	–6.41	–6.56
	-115.1°	-96.5°	-32.8°
SOMO	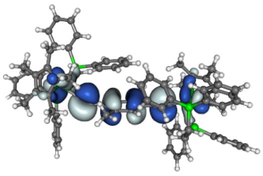	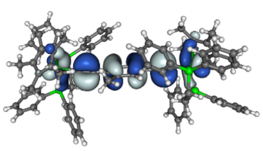	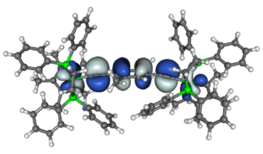
ε / eV	–3.34	–3.38	–3.34
HOMO	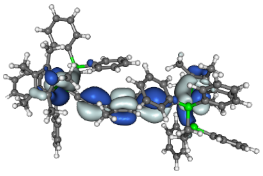	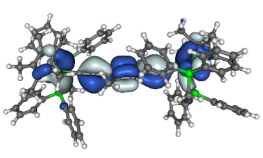	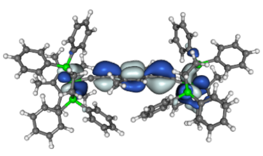

ε / eV	–5.46	–5.46	–5.47
HOMO-1	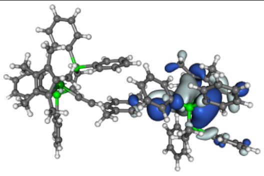	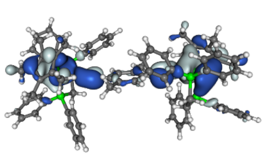	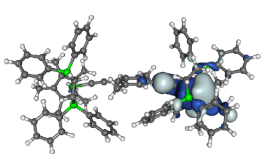
ε / eV	–6.00	–6.06	–5.95
HOMO-2	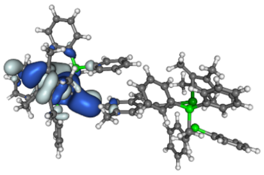	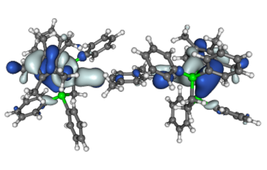	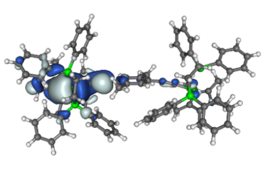
ε / eV	–6.22	–6.09	–6.26
HOMO-3	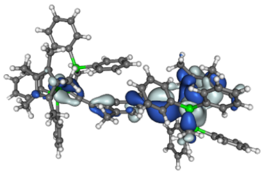	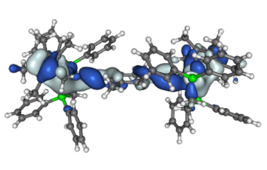	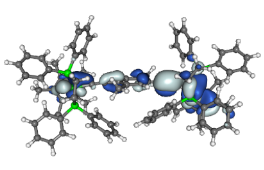
ε / eV	–6.39	–6.37	–6.30
	41.7°	62.6°	119.4°
SOMO	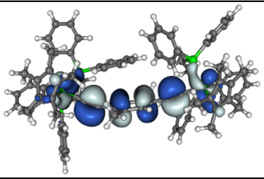	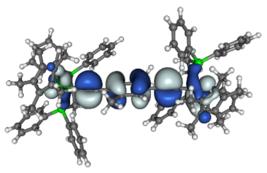	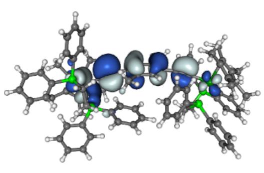
ε / eV	–3.33	–3.35	–3.36
HOMO	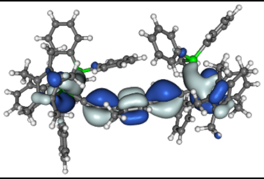	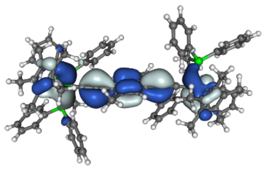	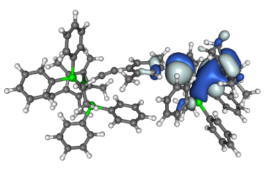
ε / eV	–5.46	–5.46	–5.61
HOMO-1	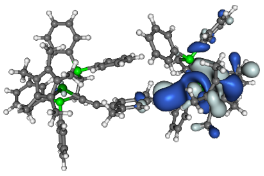	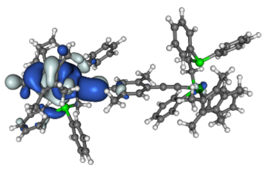	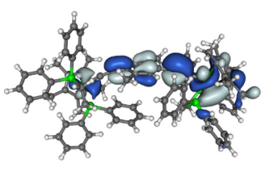
ε / eV	–6.07	–5.95	–5.65
HOMO-2	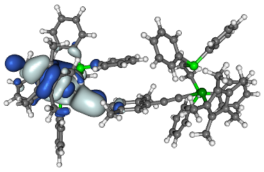	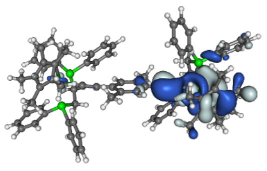	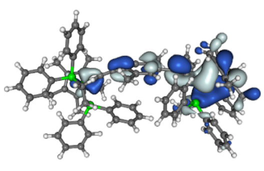

ε / eV	–6.18	–6.23	–6.09
HOMO-3	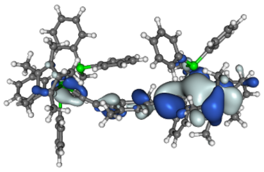	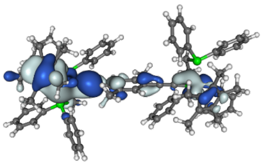	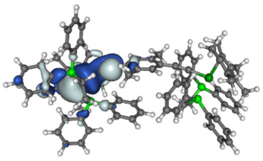
ε / eV	–6.47	–6.34	–6.45
	125.9°	146.2°	155.2°
SOMO	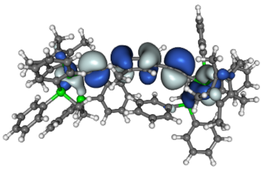	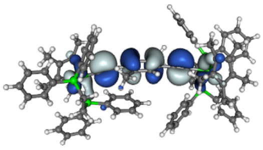	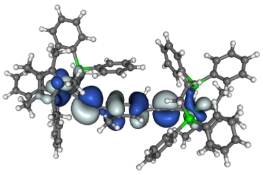
ε / eV	–3.42	–3.32	–3.34
HOMO	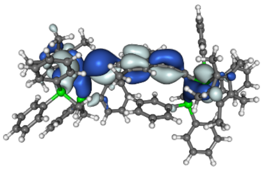	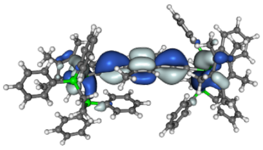	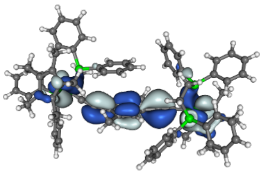
ε / eV	–5.47	–5.46	–5.48
HOMO-1	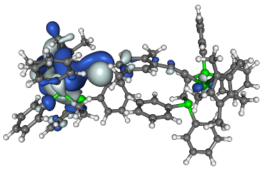	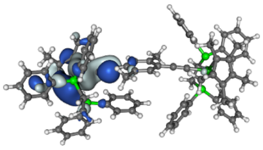	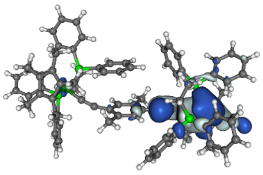
ε / eV	–5.88	–6.04	–5.90
HOMO-2	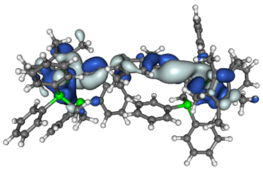	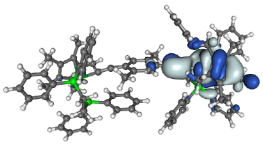	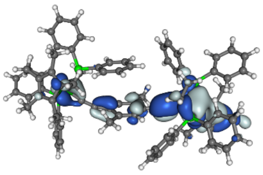
ε / eV	–6.25	–6.09	–6.24
HOMO-3	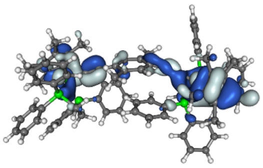	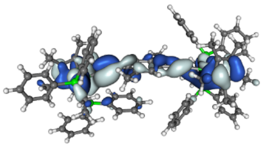	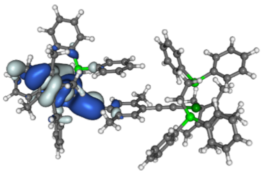
ε / eV	–6.34	–6.38	–6.30
	160.2°		
SOMO	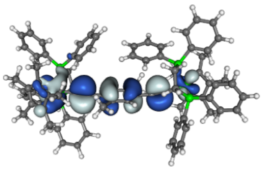		

ε / eV	–3.36		
HOMO	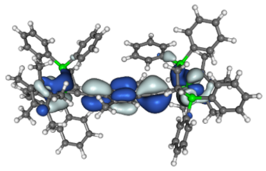		
ε / eV	–5.43		
HOMO-1	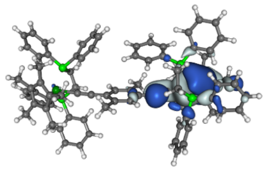		
ε / eV	–5.93		
HOMO-2	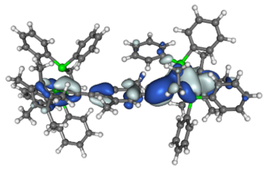		
ε / eV	–6.29		
HOMO-3	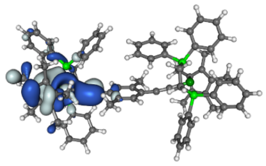		
ε / eV	–6.33		

Figure S11: Plots and energies (eV) of frontier orbitals of each conformer of [**3**]$^+$

	[**4**]$^+$		
	-169.7°	-119.2°	-64.0°
SOMO	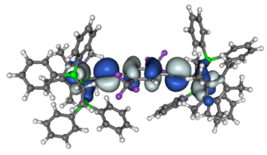	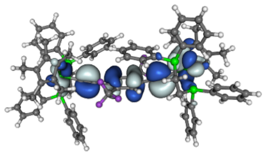	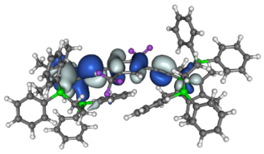
ε / eV	–3.55	–3.55	–3.58
HOMO	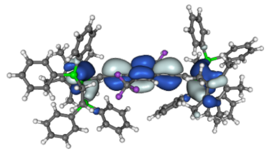	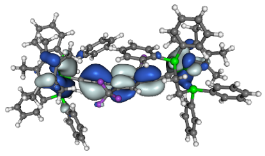	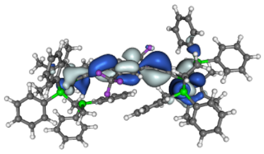
ε / eV	–5.58	–5.58	–5.58
HOMO-1	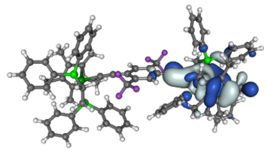	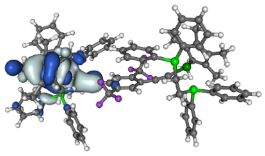	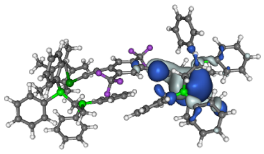
ε / eV	–6.28	–6.04	–5.97
HOMO-2	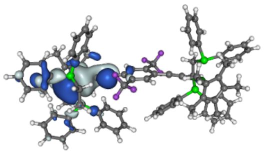	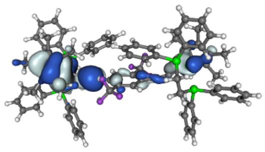	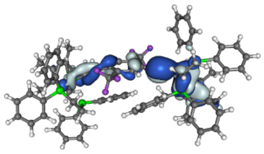
ε / eV	–6.31	–6.49	–6.37
HOMO-3	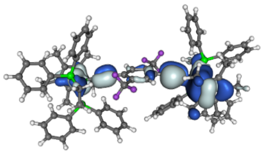	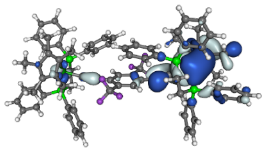	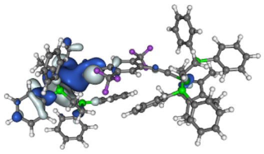
ε / eV	–6.70	–6.53	–6.64
	-35.6°	27.1°	33.2°
SOMO	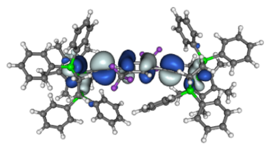	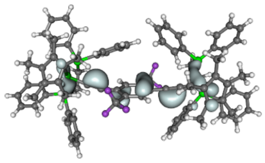	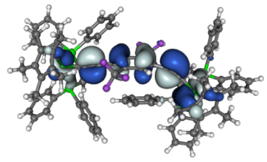
ε / eV	–3.55	–3.55	–3.56
HOMO	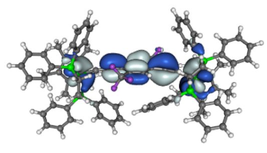	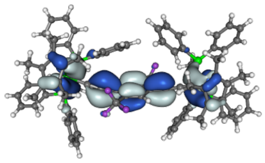	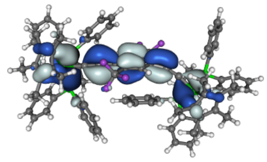

ε / eV	−5.59	−5.59	−5.59
HOMO-1	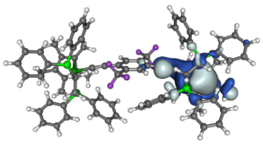	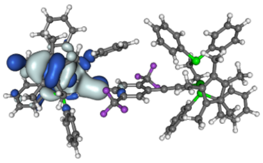	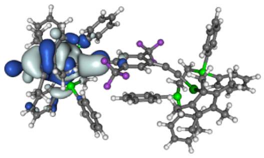
ε / eV	−6.07	−6.14	−6.05
HOMO-2	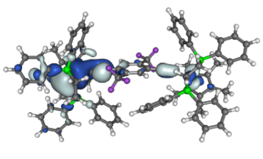	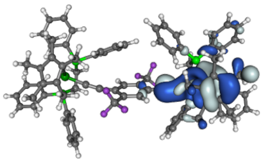	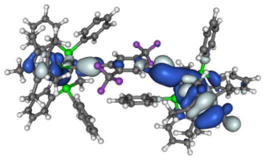
ε / eV	−6.46	−6.38	−6.49
HOMO-3	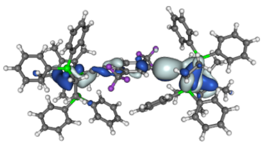	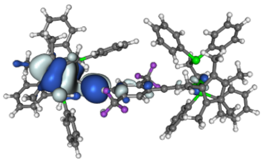	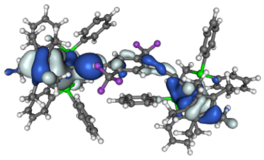
ε / eV	−6.49	−6.57	−6.50
	99.2°	122.7°	147.7°
SOMO	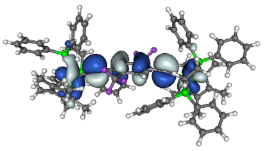	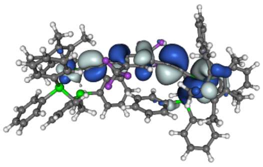	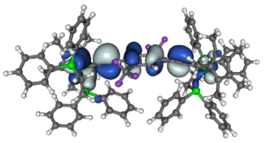
ε / eV	−3.59	−3.55	−3.56
HOMO	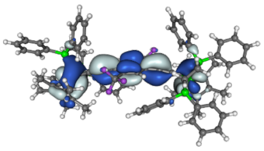	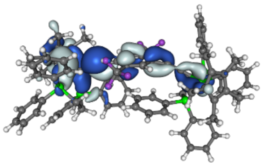	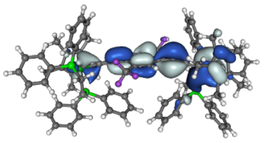
ε / eV	−5.59	−5.57	−5.58
HOMO-1	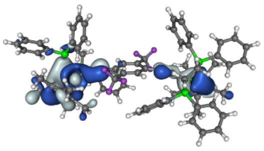	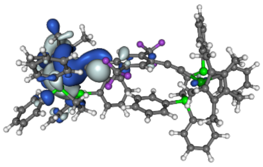	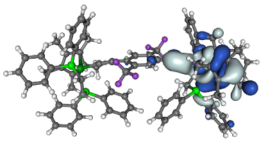
ε / eV	−6.24	−5.86	−5.96
HOMO-2	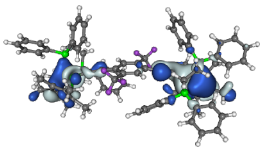	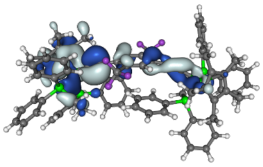	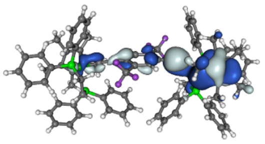

ε / eV	–6.25	–6.37	–6.40
HOMO-3	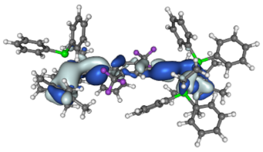	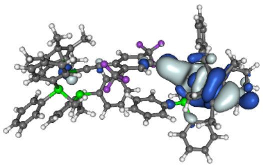	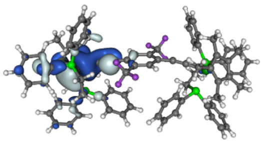
ε / eV	–6.53	–6.74	–6.63
	164.6°		
SOMO	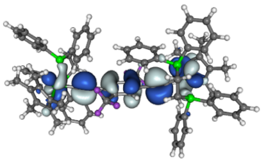		
ε / eV	–3.56		
HOMO	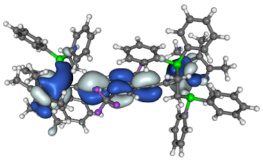		
ε / eV	–5.54		
HOMO-1	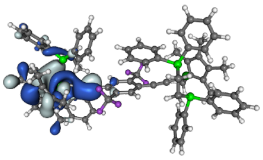		
ε / eV	–5.86		
HOMO-2	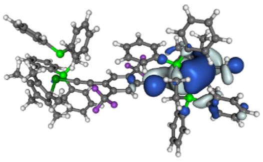		
ε / eV	–6.37		
HOMO-3	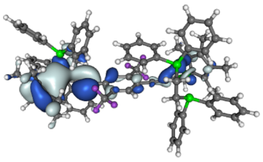		
ε / eV	–6.74		

Figure S12: Plots and energies (eV) of frontier orbitals of each conformer of [**4**]$^+$

	[**5**]$^{+}$		
	-165.4°	-148.9°	-132.6°
SOMO	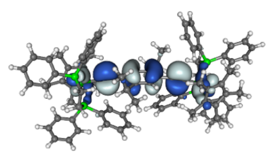	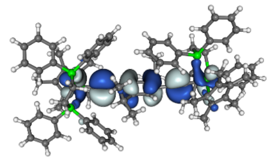	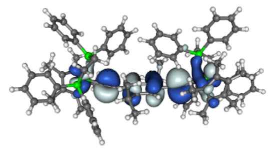
ε / eV	–3.29	–3.33	–132.6
HOMO	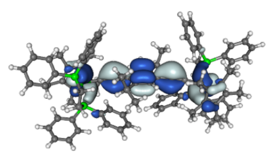	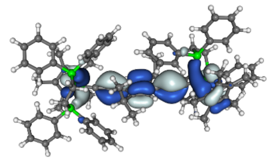	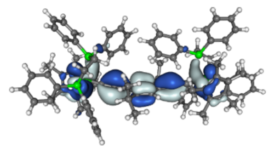
ε / eV	–5.47	–5.48	–5.47
HOMO-1	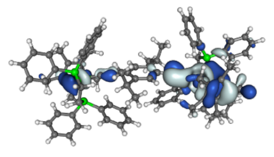	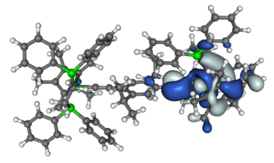	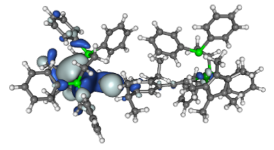
ε / eV	–6.06	–5.95	–5.92
HOMO-2	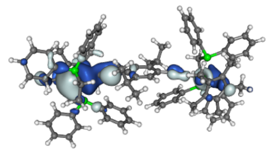	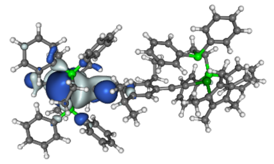	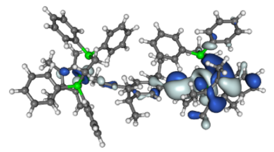
ε / eV	–6.07	–6.13	–6.19
HOMO-3	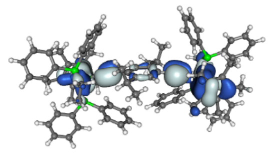	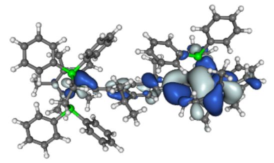	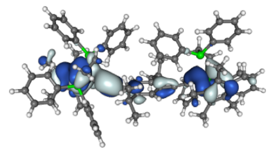
ε / eV	–6.54	–6.37	–6.30
	-68.9°	-62.0°	-14.9°
SOMO	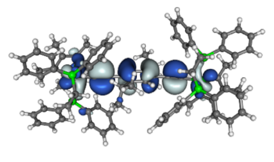	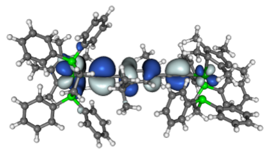	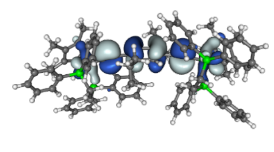
ε / eV	–3.35	–3.36	–3.32
HOMO	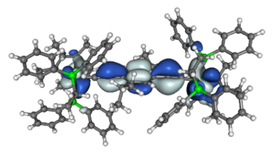	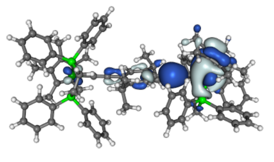	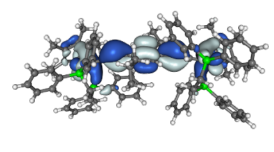

ε / eV	–5.47	–5.57	–5.45
HOMO-1	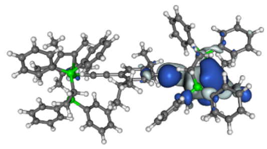	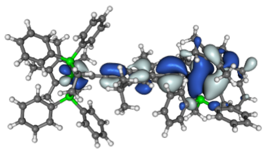	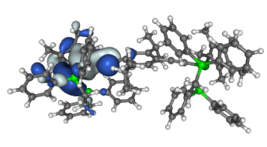
ε / eV	–5.90	–5.64	–5.96
HOMO-2	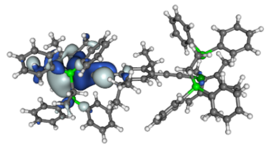	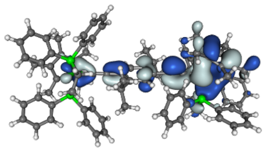	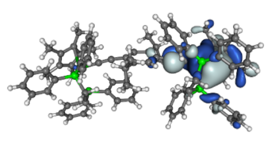
ε / eV	–6.21	–6.09	–6.15
HOMO-3	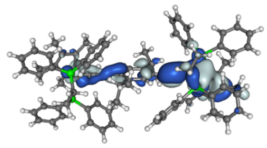	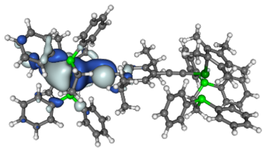	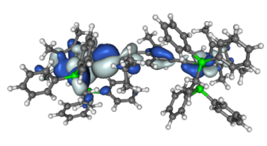
ε / eV	–6.23	–6.41	–6.33
	28.4°	34.7°	104.6°
SOMO	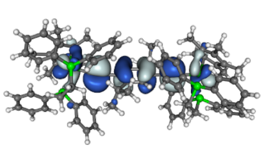	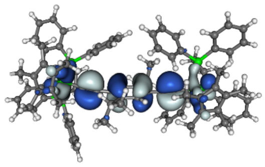	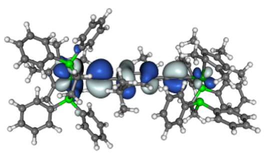
ε / eV	–3.34	–3.31	–3.36
HOMO	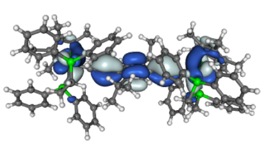	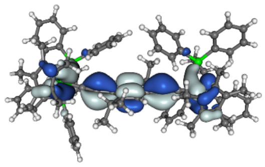	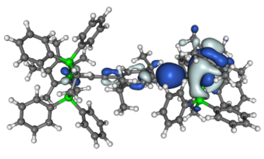
ε / eV	–5.47	–5.45	–5.57
HOMO-1	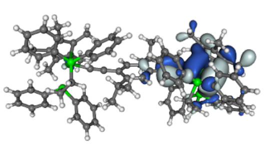	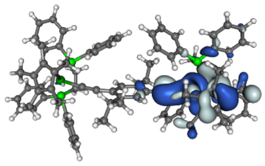	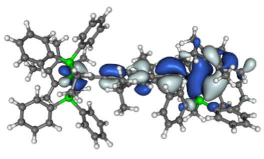
ε / eV	–5.95	–6.04	–5.62
HOMO-2	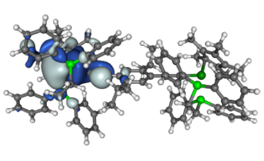	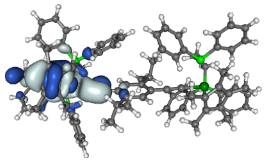	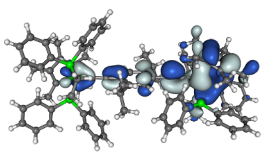

ε / eV	–6.18	–6.13	–6.07
HOMO-3	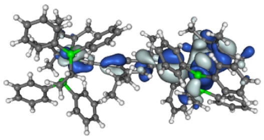	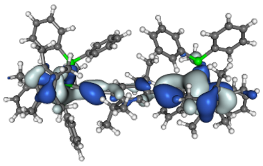	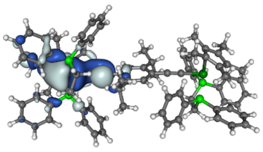
ε / eV	–6.28	–6.49	–6.48
	165.5°		
SOMO	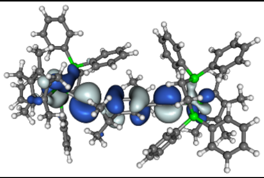		
ε / eV	–3.32		
HOMO	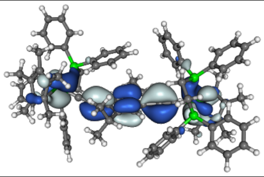		
ε / eV	–5.44		
HOMO-1	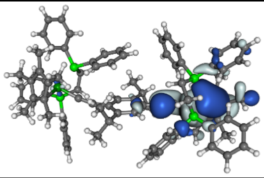		
ε / eV	–5.89		
HOMO-2	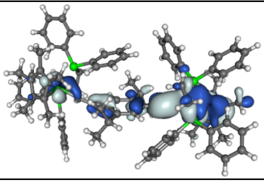		
ε / eV	–6.27		
HOMO-3	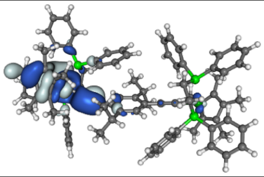		
ε / eV	–6.32		

Figure S13: Plots and energies (eV) of frontier orbitals of each conformer of [**5**]$^{+}$

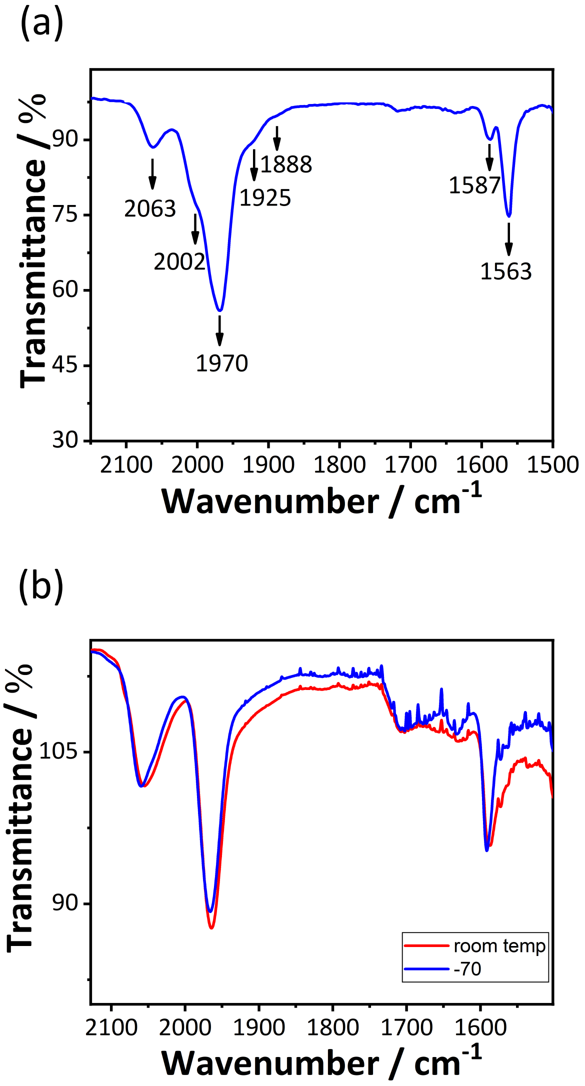

Figure S14. The IR spectra of [**1a**]PF_6: (a) in CH_2Cl_2 solution; (b) from solid samples precipitated from CH_2Cl_2 solution at room temperature (red) or -78°C (blue) by addition of Et_2O and recorded as a Nujol mull.

600 MHz, CDCl3

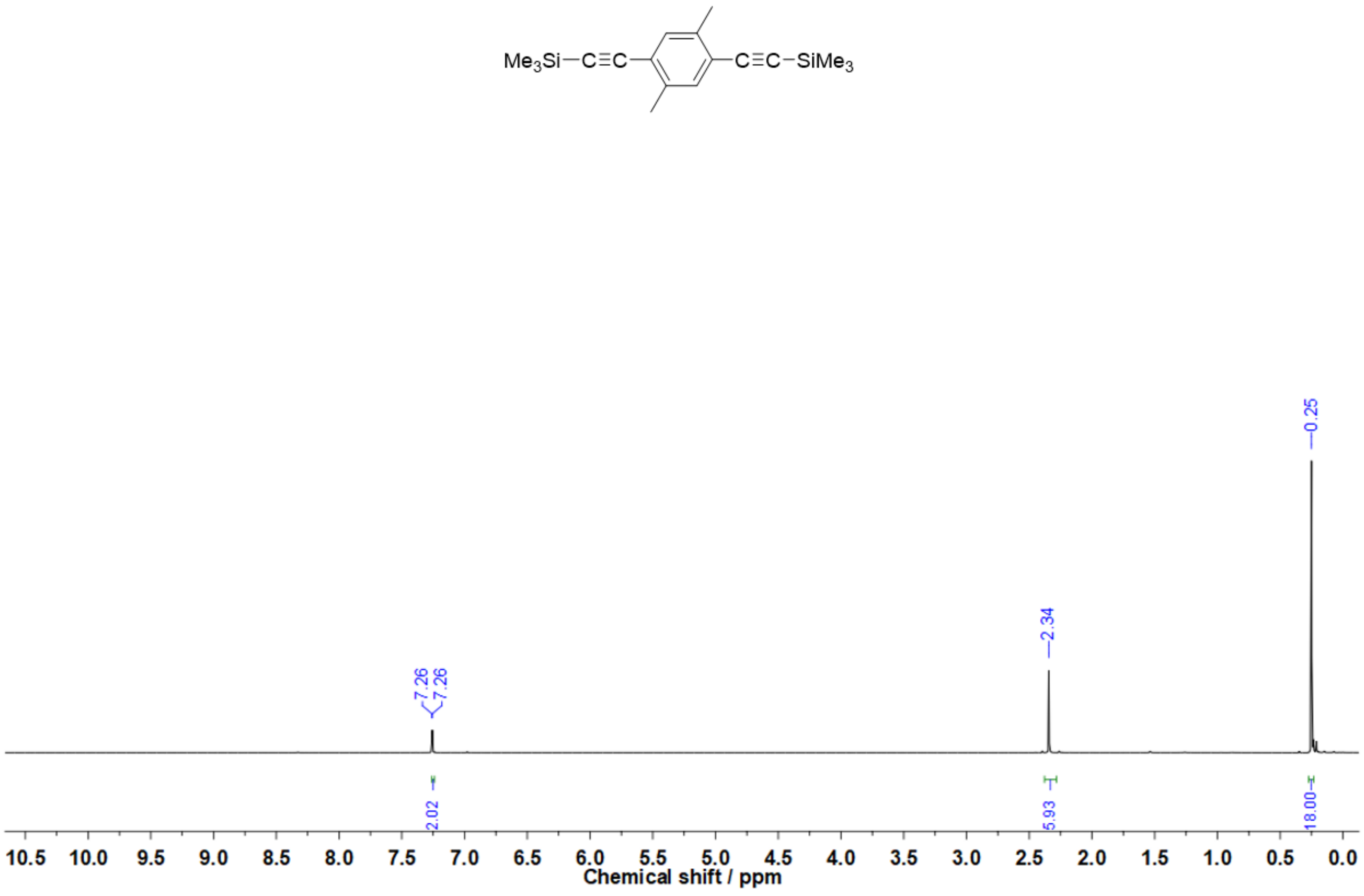

Figure S15. The ^{1}H NMR spectrum of 1,4-bis((trimethylsilyl)ethynyl)-2,5-dimethylbenzene.

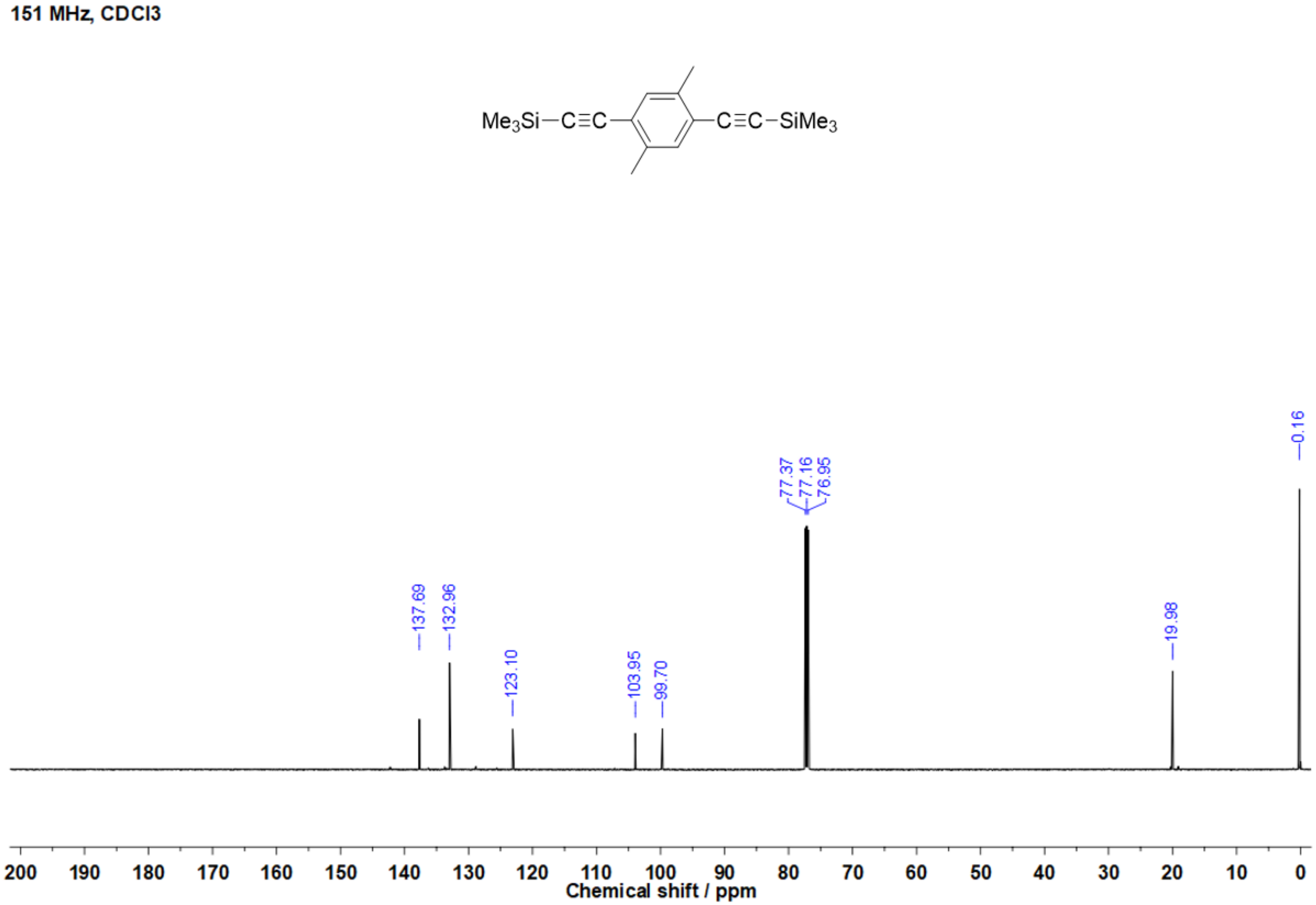

Figure S16. The ${}^{13}C\{{}^{1}H\}$ NMR spectrum of 1,4-bis((trimethylsilyl)ethynyl)-2,5-dimethylbenzene**.**

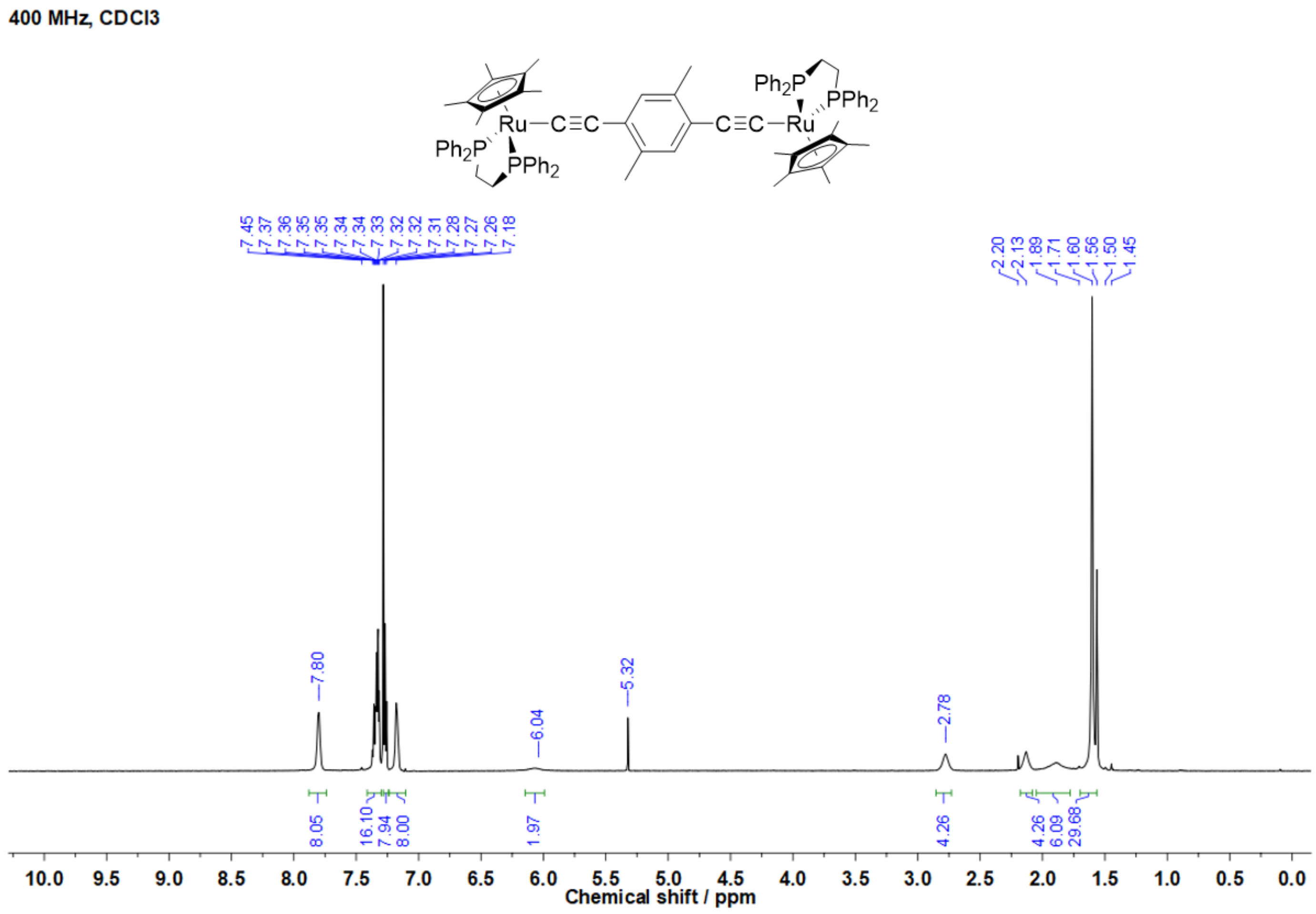

Figure S17. The ^{1}H NMR spectrum of [{Ru(dppe)Cp*}$_2$(μ-C≡C-1,4-C_6H_2{2,5-(CH_3)$_2$}-C≡C)] **(3a).**

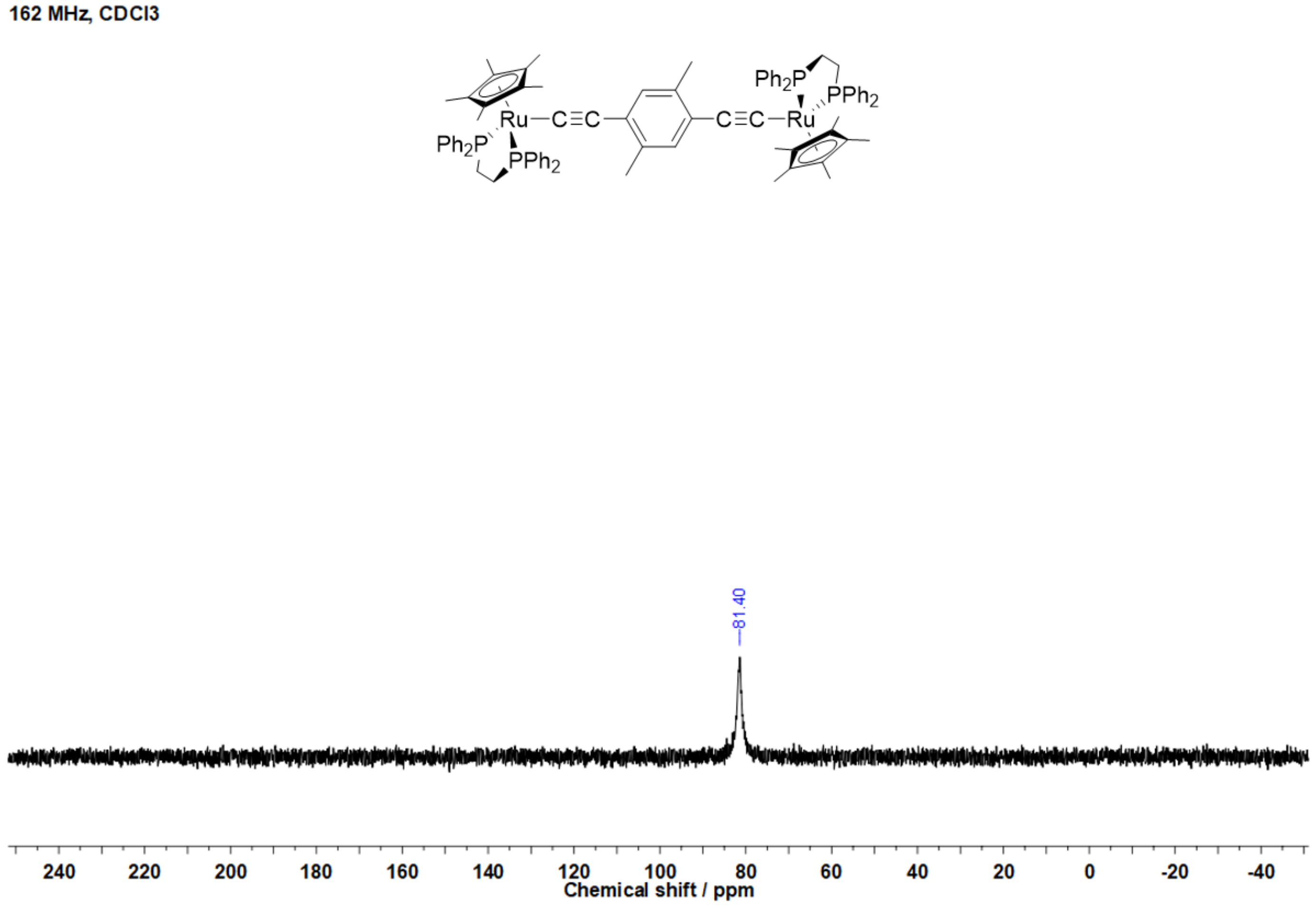

Figure S18. The ^{31}P{^{1}H} NMR spectrum of [{Ru(dppe)Cp*}$_2$(μ-C≡C-1,4-C_6H_2{2,5-(CH_3)$_2$}-C≡C)] (**3a**) in $CDCl_3$.

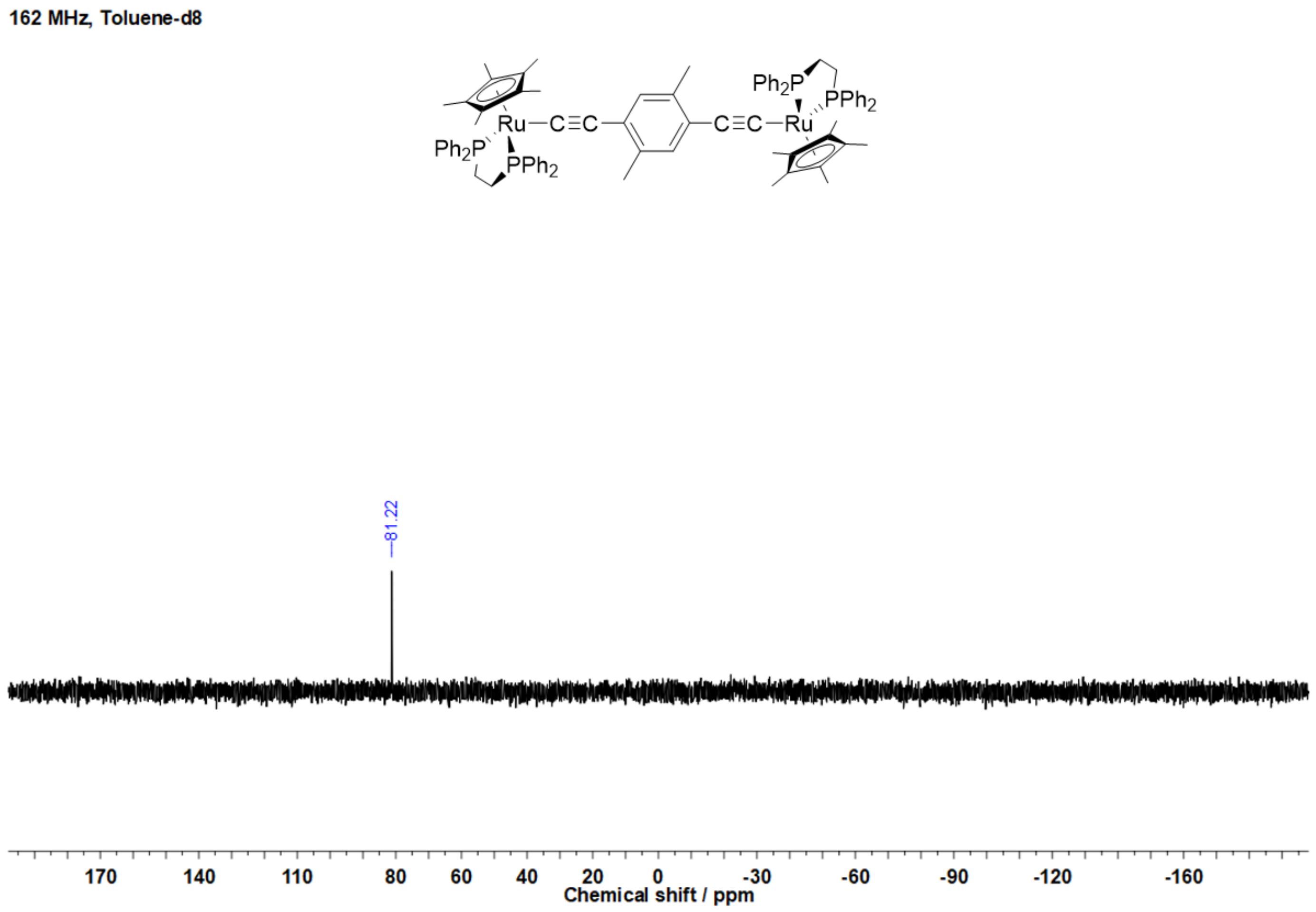

Figure S19. The $^{31}P\{^1H\}$ NMR spectrum of [{Ru(dppe)Cp*}$_2$(μ-C≡C-1,4-C_6H_2\{2,5-$(CH_3)_2$\}-C≡C)] (**3a**) in toluene-d_8.

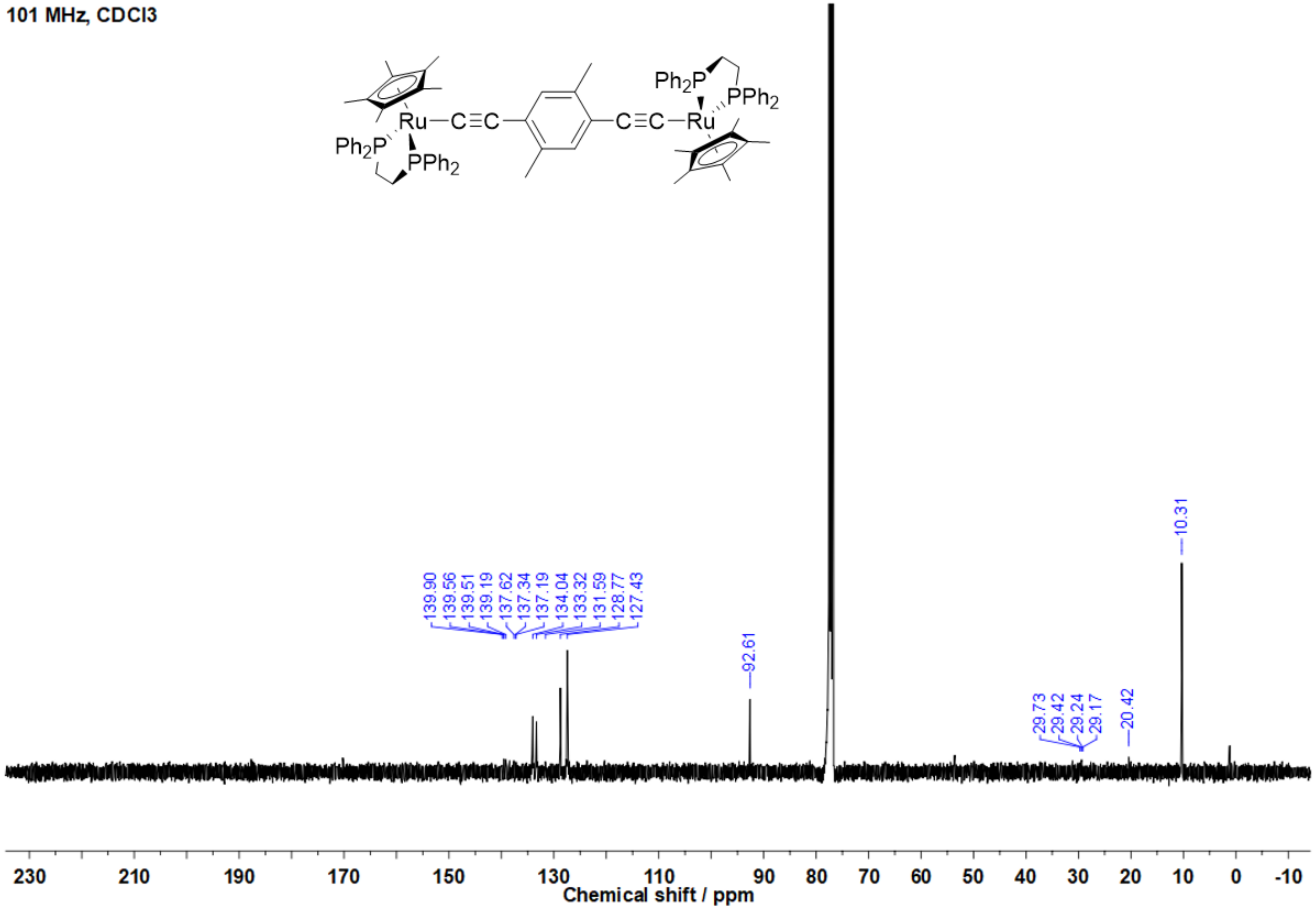

Figure S20. The $^{13}C\{^{1}H\}$ NMR spectrum of [{Ru(dppe)Cp*}$_2$(μ-C≡C-1,4-C_6H_2\{2,5-$(CH_3)_2$\}-C≡C)] **(3a).**

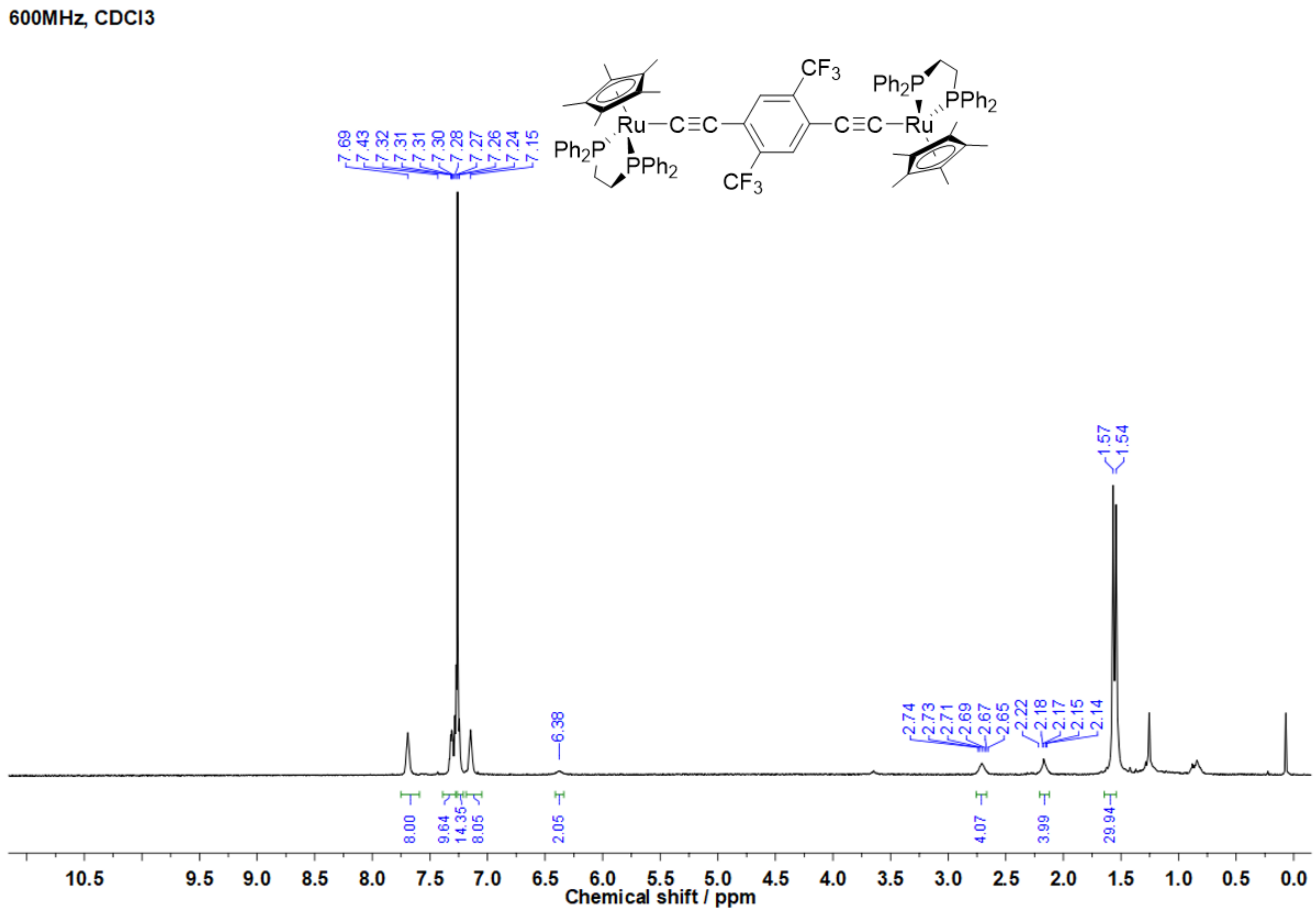

Figure S21. The ^{1}H NMR spectrum of [{Ru(dppe)Cp*}$_2$(μ-C≡C-1,4-C_6H_2{2,5-(CF_3)$_2$}-C≡C)] **(4a).**

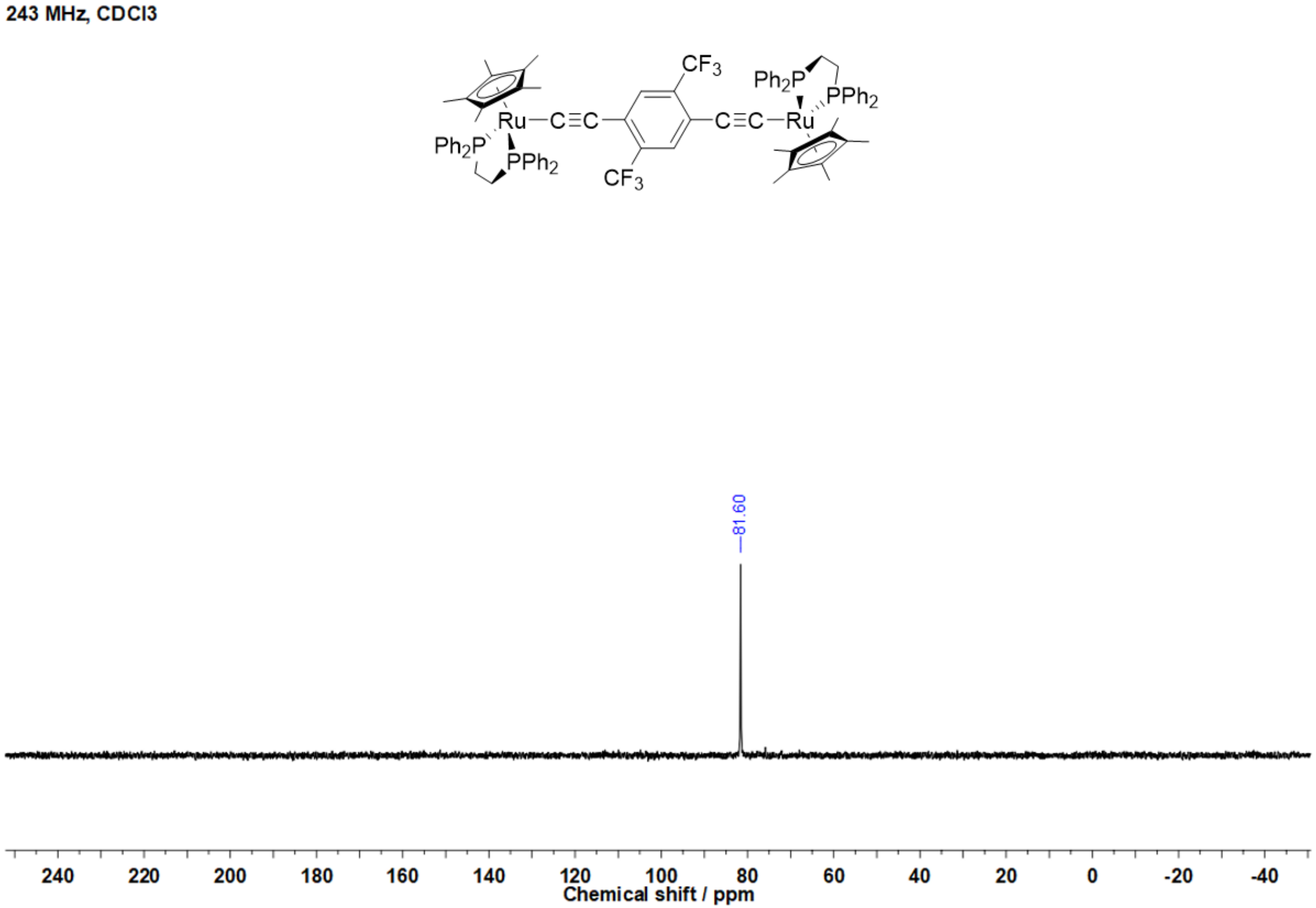

Figure S22. The $^{31}P\{^{1}H\}$ NMR spectrum of $[\{Ru(dppe)Cp^*\}_2(\mu\text{-}C{\equiv}C\text{-}1,4\text{-}C_6H_2\{2,5\text{-}(CF_3)_2\}\text{-}C{\equiv}C)]$ **(4a).**

565 MHz, CDCl3

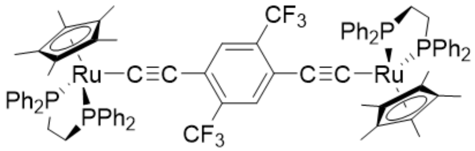

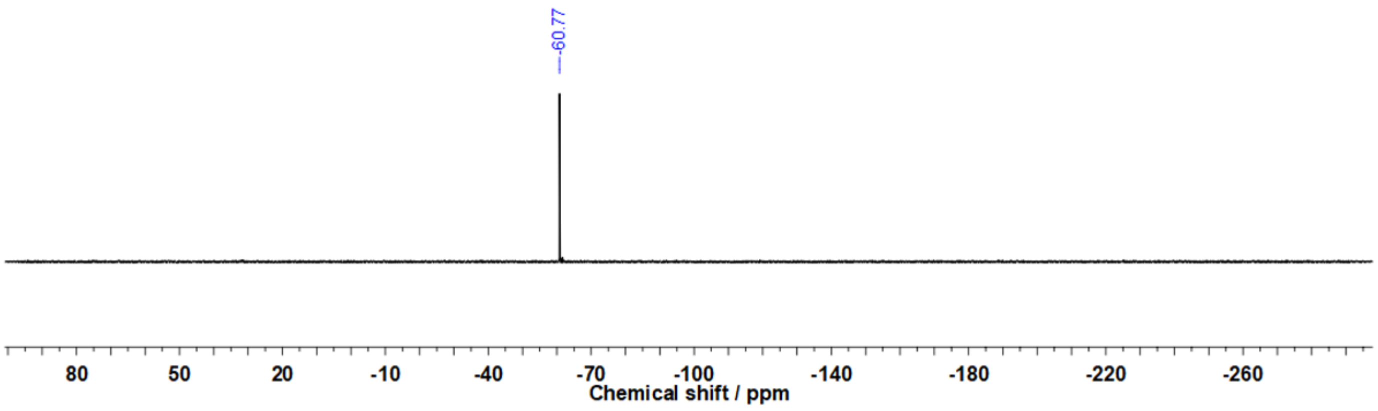

Figure S23. The $^{19}F\{^{1}H\}$ NMR spectrum of [{Ru(dppe)Cp*}$_2$(μ-C≡C-1,4-C_6H_2\{2,5-(CF_3)$_2$\}-C≡C)] **(4a).**

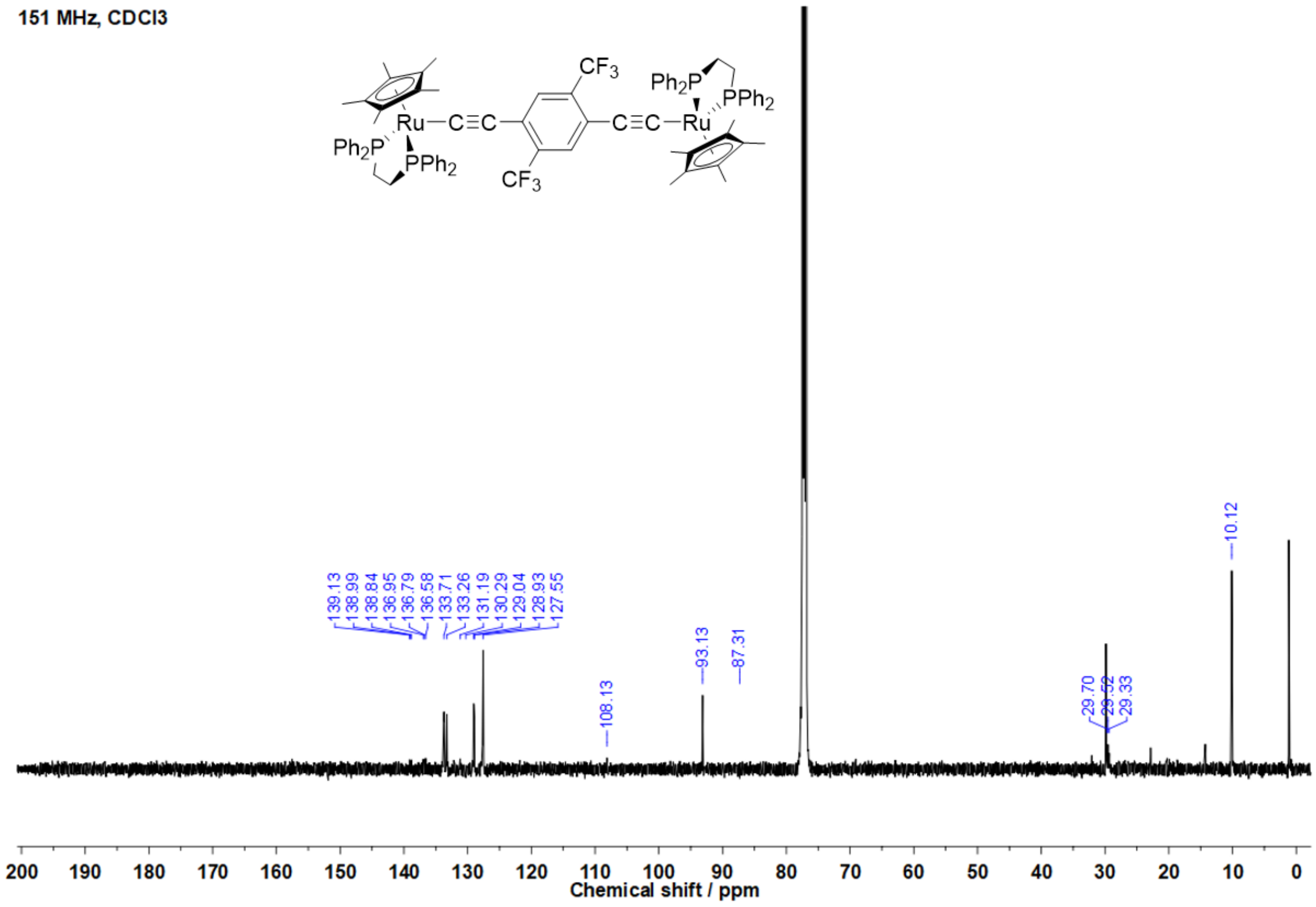

Figure S24. The $^{13}C\{^{1}H\}$ NMR spectrum of [{Ru(dppe)Cp*}$_2$(μ-C≡C-1,4-C_6H_2{2,5-$(CF_3)_2$}-C≡C)] **(4a).**

400 MHz, CDCl3

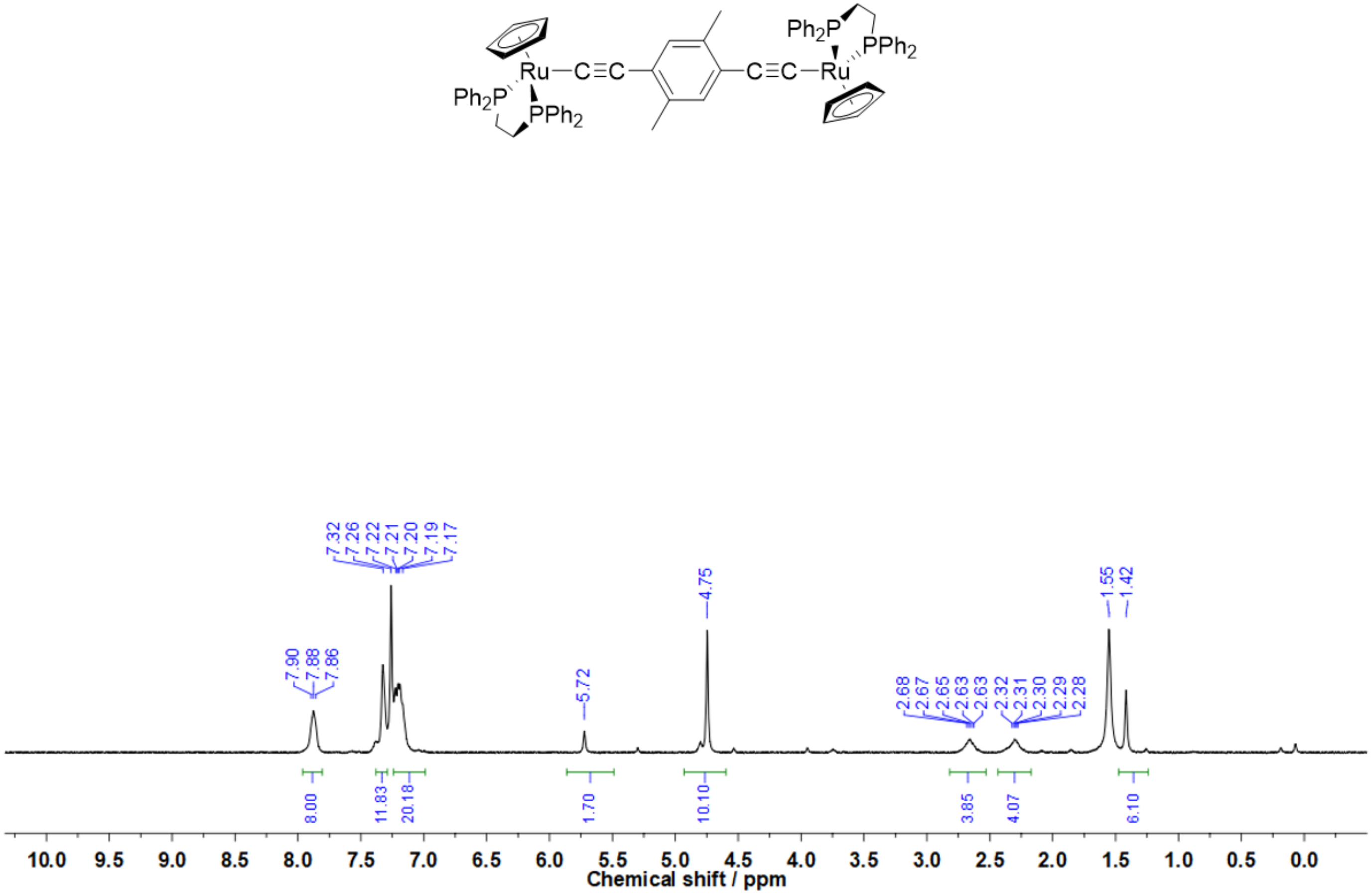

Figure S25. The ^{1}H NMR spectrum of [{Ru(dppe)Cp}$_2$(μ-C≡C-1,4-C_6H_2{2,5-(CH_3)$_2$}-C≡C)] **(3b).**

162 MHz, CDCl3

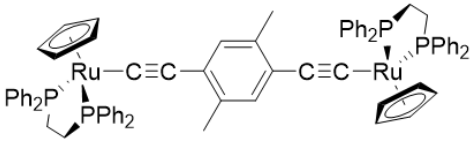

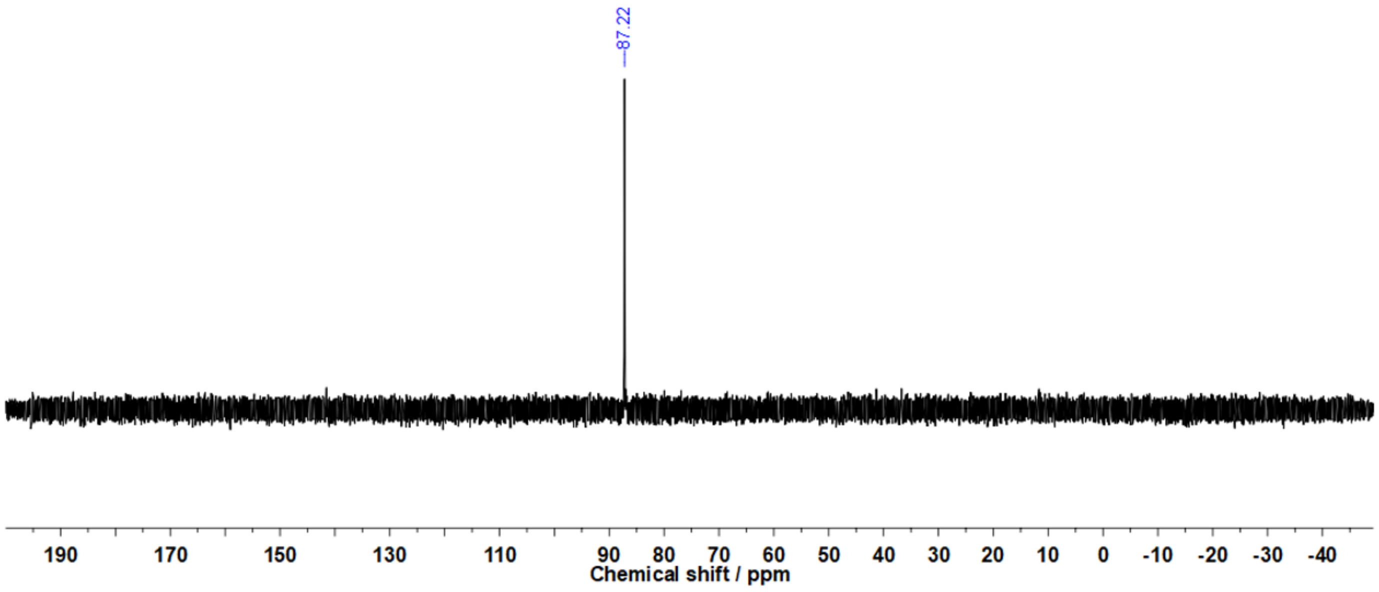

Figure S26. The $^{31}P\{^1H\}$ NMR spectrum of [{Ru(dppe)Cp}$_2$(μ-C≡C-1,4-C_6H_2{2,5-$(CH_3)_2$}-C≡C)] **(3b).**

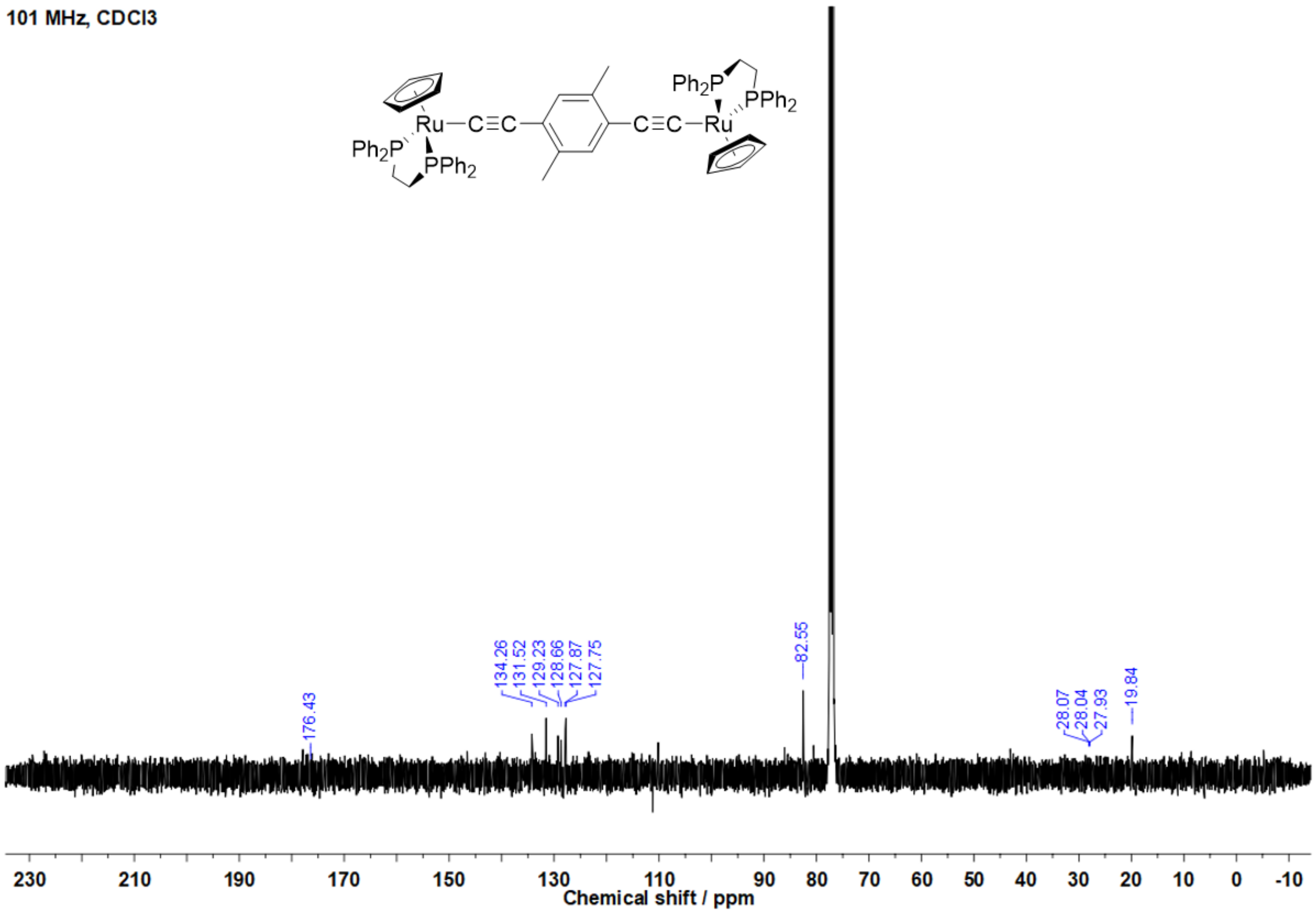

Figure S27. The $^{13}C\{^1H\}$ NMR spectrum of [{Ru(dppe)Cp}$_2$(μ-C≡C-1,4-C_6H_2\{2,5-$(CH_3)_2$\}-C≡C)] **(3b).**

500 MHz, CDCl3

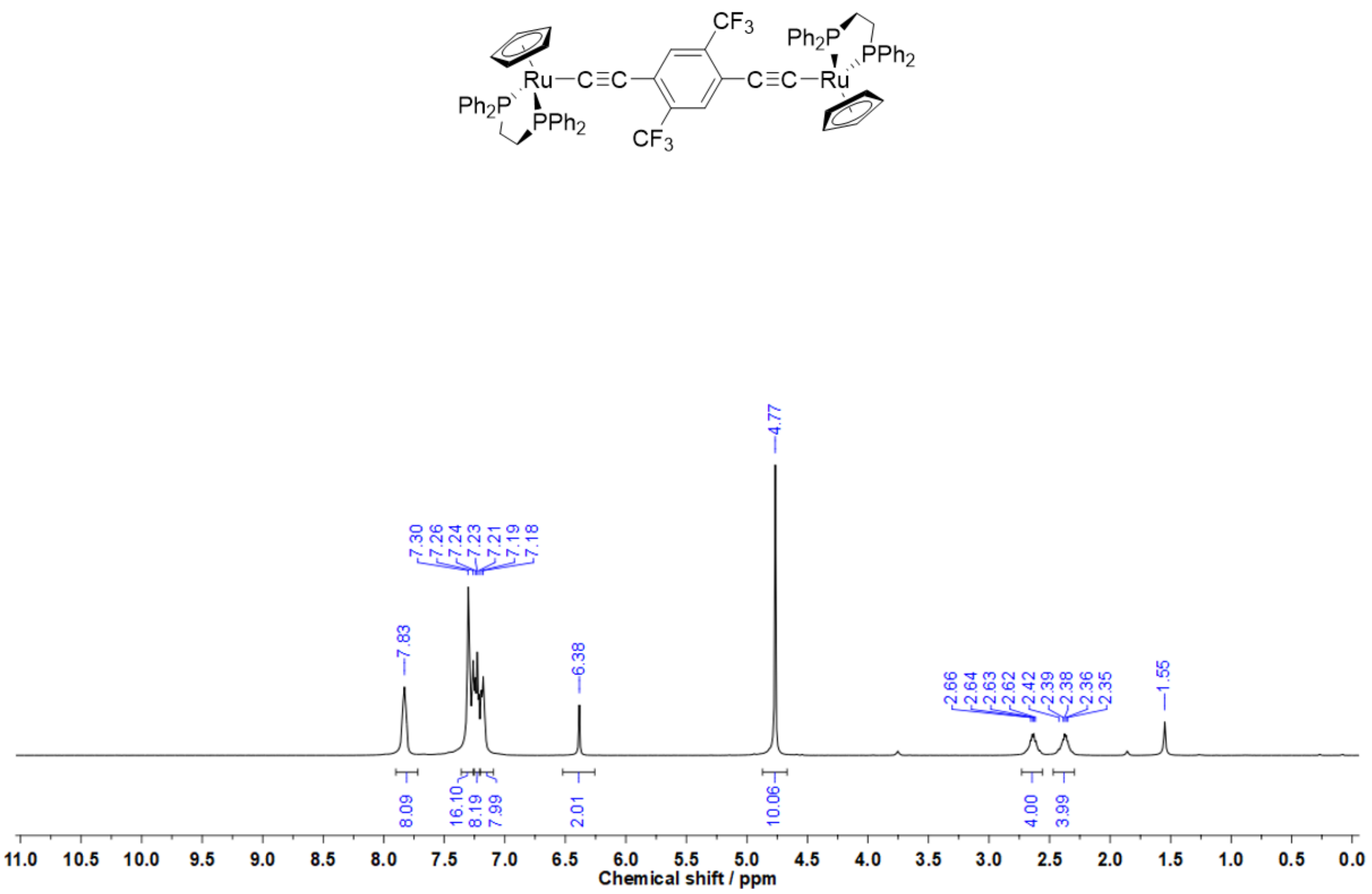

Figure S28. The ^{1}H NMR spectrum of [{Ru(dppe)Cp}$_2$(μ-C≡C-1,4-C_6H_2{2,5-(CF_3)$_2$}-C≡C)] **(4b).**

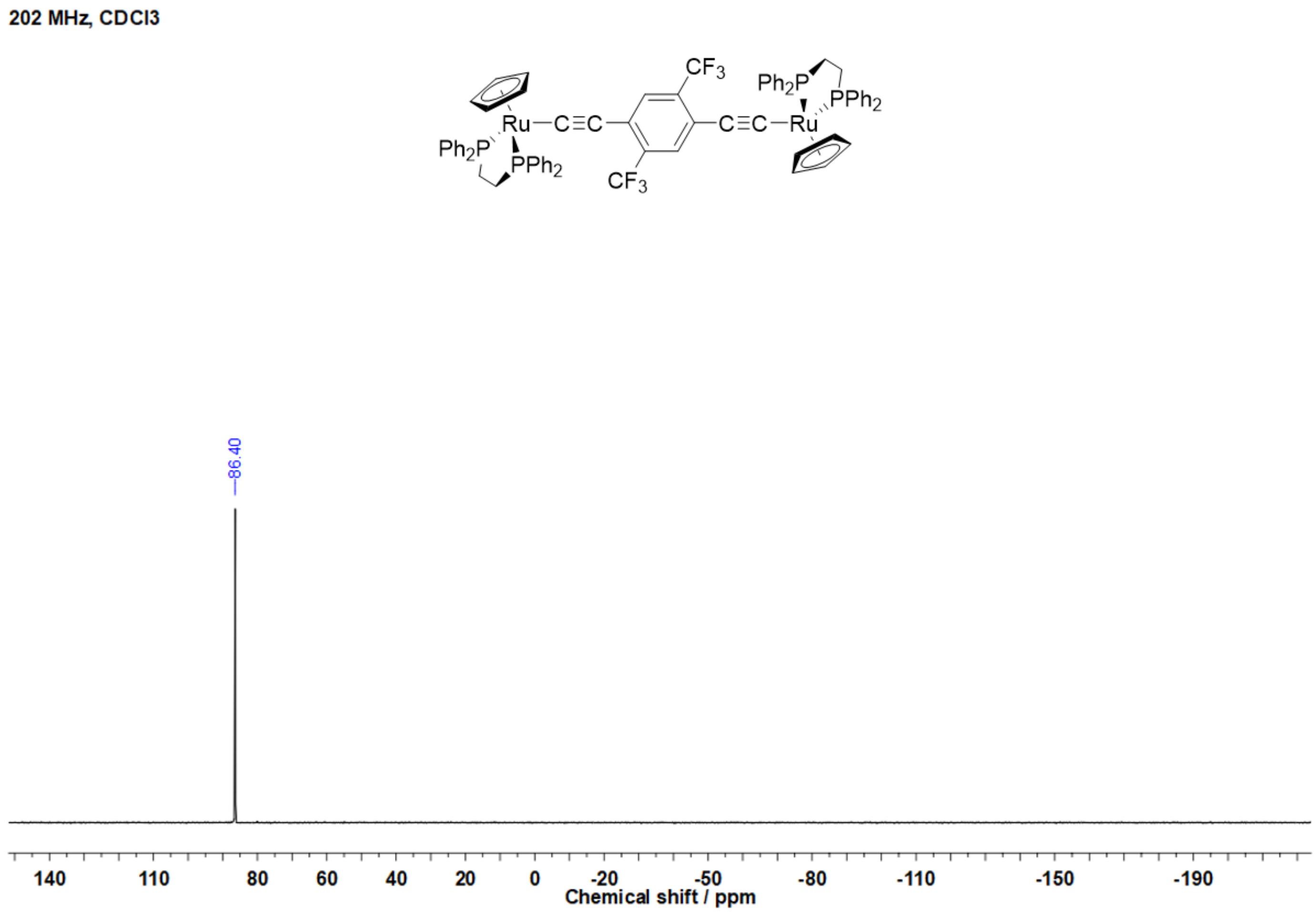

Figure S29. The $^{31}P\{^{1}H\}$ NMR spectrum of [{Ru(dppe)Cp}$_2$(μ-C≡C-1,4-C_6H_2\{2,5-$(CF_3)_2$\}-C≡C)] **(4b).**

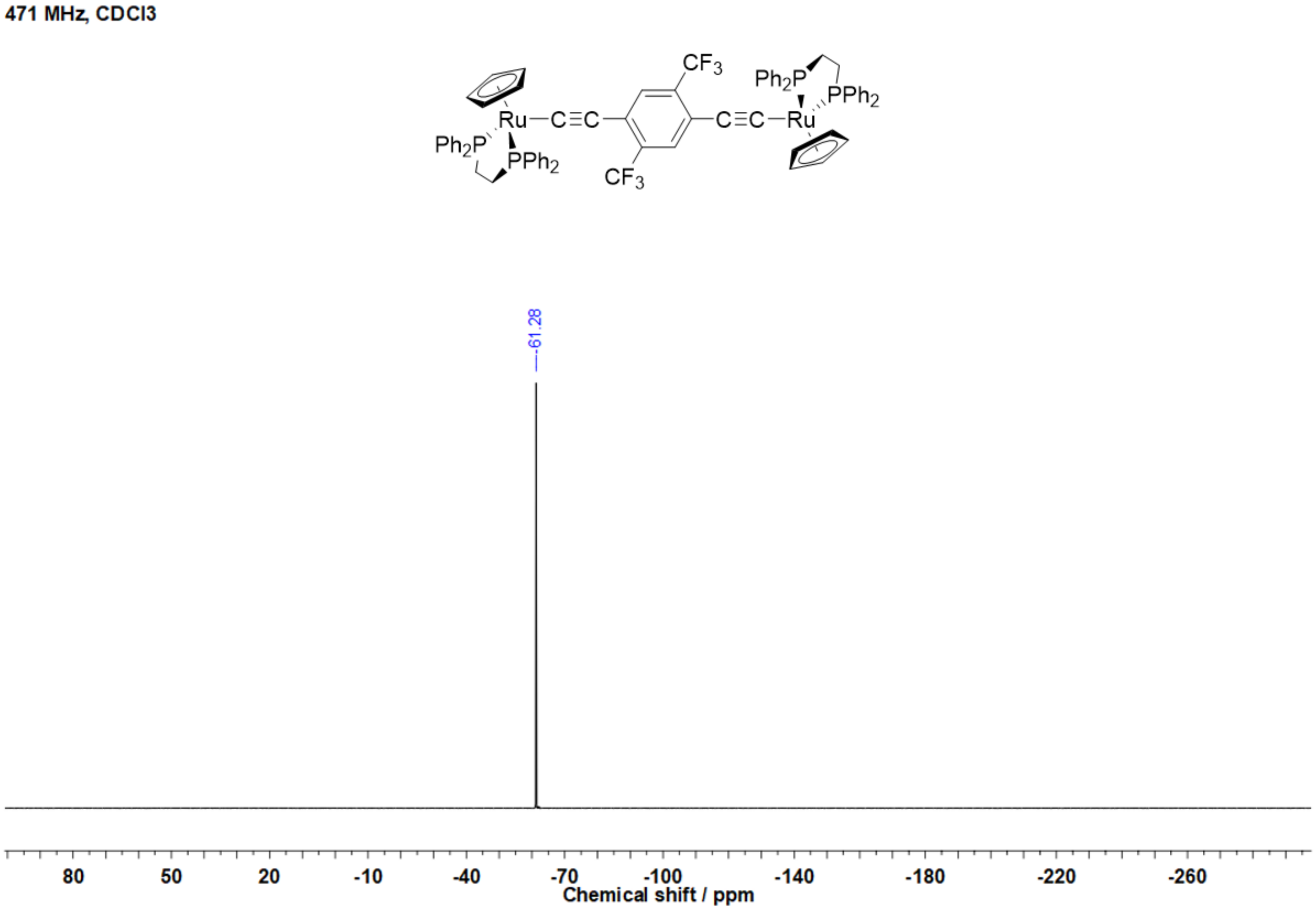

Figure S30. The $^{19}F\{^{1}H\}$ NMR spectrum of [{Ru(dppe)Cp}$_2$(μ-C≡C-1,4-C_6H_2\{2,5-(CF_3)$_2$\}-C≡C)] **(4b).**

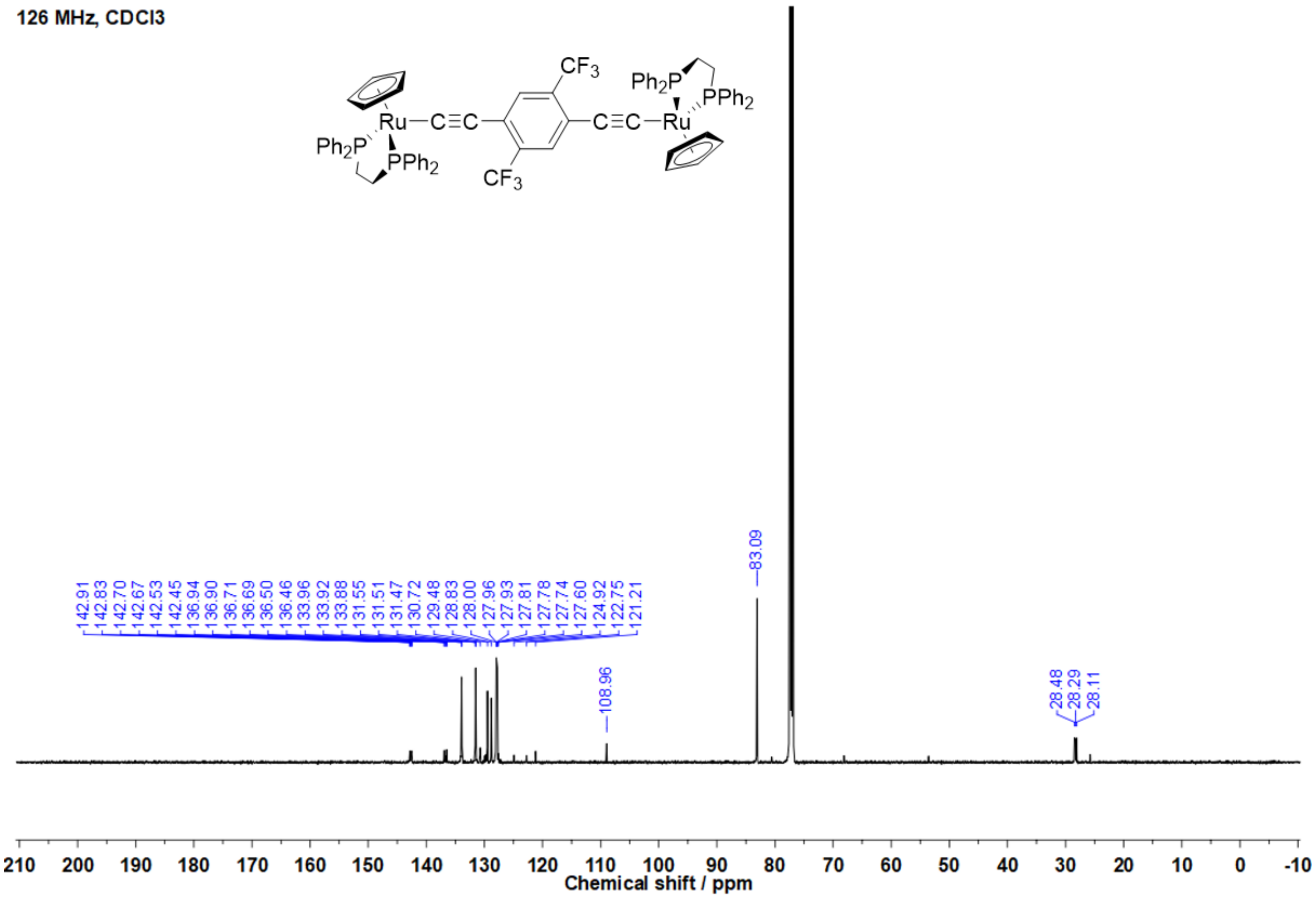

Figure S31. The $^{13}C\{^{1}H\}$ NMR spectrum of [{Ru(dppe)Cp}$_2$(μ-C≡C-1,4-C_6H_2\{2,5-(CF_3)$_2$\}-C≡C)] **(4b).**

400 MHz, CDCl3

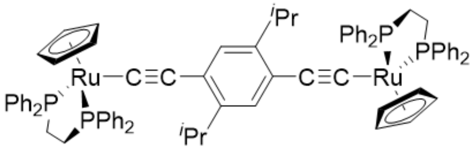

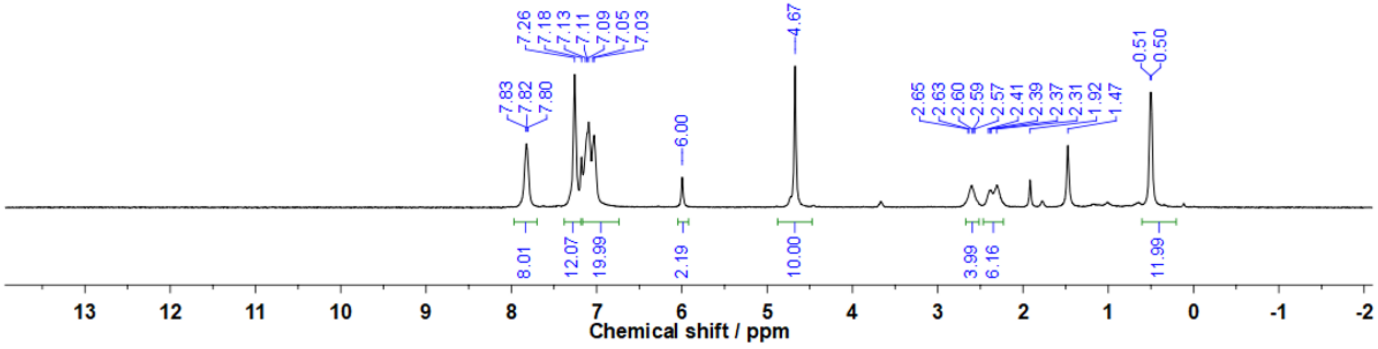

Figure S32. The ^{1}H NMR spectrum of [{Ru(dppe)Cp}$_2$(μ-C≡C-1,4-C_6H_2-{2,5-CH(CH_3)$_2$}$_2$}-C≡C)] **(5b).**

162 MHz, CDCl3

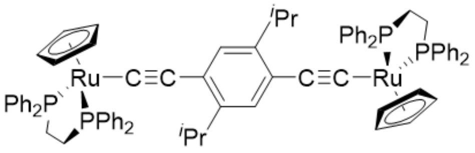

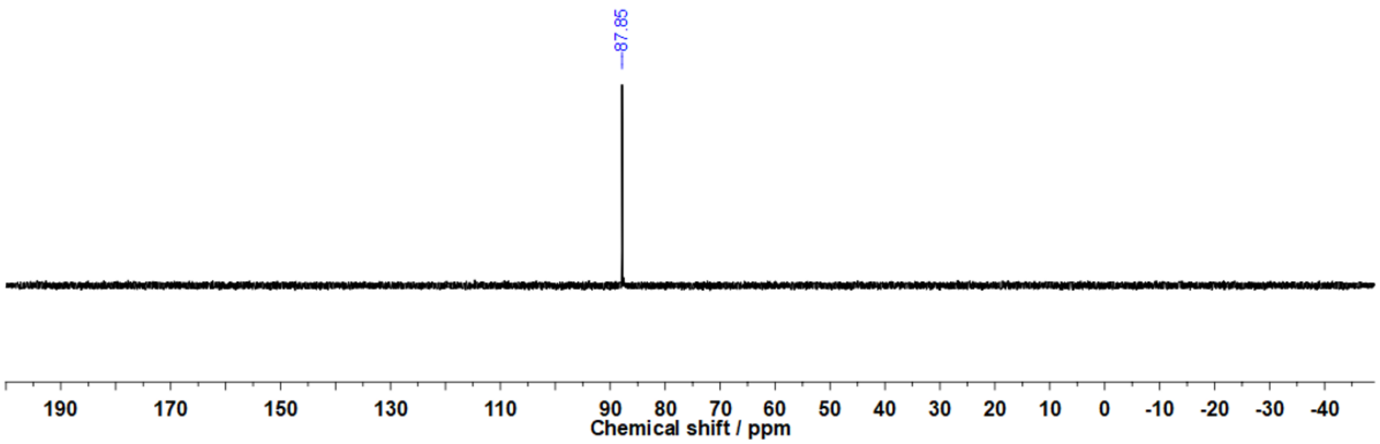

Figure S33. The $^{31}P\{^1H\}$ NMR spectrum of [{Ru(dppe)Cp}$_2$(μ-C≡C-1,4-C_6H_2-{2,5-$CH(CH_3)_2$}$_2$-C≡C)] **(5b).**

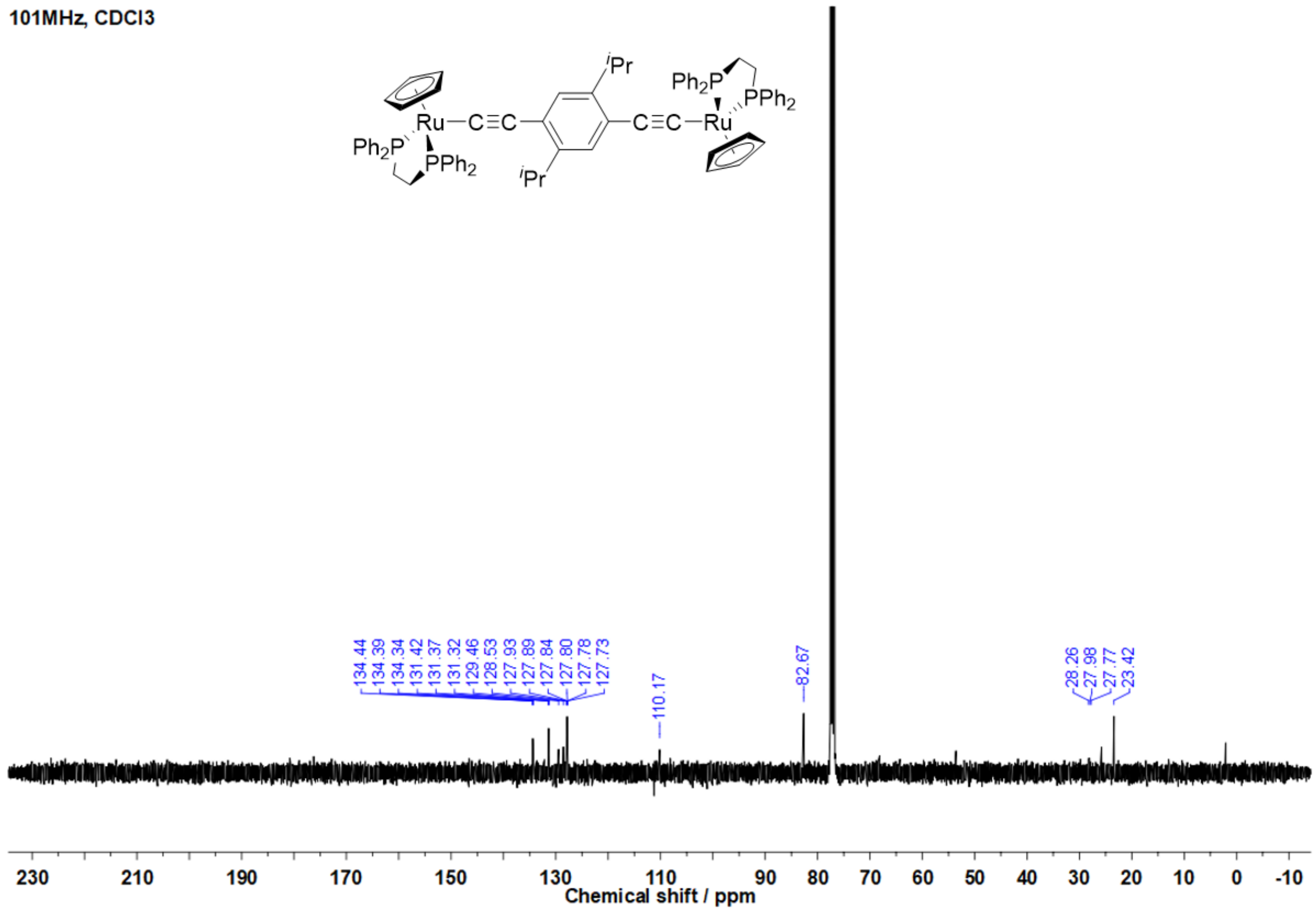

Figure S34. The ^{13}C{^{1}H} NMR spectrum of [{Ru(dppe)Cp}$_2$(μ-C≡C-1,4-C_6H_2-{2,5-$CH(CH_3)_2$}$_2$-C≡C)] **(5b).**

References

[1] J. B. G. Gluyas, N. J. Brown, J. D. Farmer, P. J. Low, *Aust. J. Chem.* **2017**, *70*, 113-119.

[2] A. Nagai, J. Miyake, K. Kokado, Y. Nagata, Y. Chujo, *J. Am. Chem. Soc.* **2008**, *130*, 15276-15278.

[3] D. J. Armitt, M. I. Bruce, M. Gaudio, N. N. Zaitseva, B. W. Skelton, A. H. White, B. Le Guennic, J. F. Halet, M. A. Fox, R. L. Roberts, F. Hartl, P. J. Low, *Dalton Trans.* **2008**, 6763-6775.

[4] M. Auffray, F. Charra, L. S. Vargas, F. Mathevet, A. J. Attias, D. Kreher, *New J. Chem.* **2020**, *44*, 7665-7674.

[5] Rigaku Oxford Diffraction, *CrysAlisPro* 1.171.141.103a **2021**. Available online: https://www.rigaku.com/products/crystallography/crysalis.

[6] I. Uson, G. M. Sheldrick, *Acta Crystallogr. D* **2018**, *74*, 106-116.

[7] O. V. Dolomanov, L. J. Bourhis, R. J. Gildea, J. A. K. Howard, H. Puschmann, *J Appl. Crystallogr.* **2009**, *42*, 339-341.

[8] A. Thorn, G. M. Sheldrick, *Acta Crystallogr. A* **2008**, *64*, C221-C221.
